# The potential of *Valeriana* as a traditional Chinese medicine: traditional clinical applications, bioactivities, and phytochemistry

**DOI:** 10.3389/fphar.2022.973138

**Published:** 2022-09-21

**Authors:** Jianchun Li, Xiaoliang Li, Changfu Wang, Manli Zhang, Minhui Ye, Qiuhong Wang

**Affiliations:** ^1^ Engineering Technology Research Center for Standardized Processing of Chinese Materia Medica, College of TCM, Guangdong Pharmaceutical University, Guangzhou, China; ^2^ Key Laboratory of Tropical Translational Medicine of Ministry of Education, Hainan Provincial Key Laboratory for Research and Development of Tropical Herbs, Haikou Key Laboratory of Li Nationality Medicine, School of Pharmacy, Hainan Medical University, Haikou, China

**Keywords:** Valeriana plants, clinical application, phytochemistry, active constituent, pharmacological effect

## Abstract

*Valeriana* plants are members of the *Caprifoliaceae* family, which include more than 200 species worldwide. We summarized previous reports on traditional clinical applications, bioactivities, and phytochemistry of *Valeriana* by searching electronic databases of Science Direct, Web of Science, PubMed, and some books. Some *Valeriana* species have been used as traditional medicines, demonstrating calming fright and tranquilizing mind, promoting Qi and blood, activating blood circulation and regulating menstruation, dispelling wind and eliminating dampness, regulating Qi-flowing to relieve pain, and promoting digestion and checking diarrhea, and treating diseases of the nervous, cardiovascular, and digestive systems, inflammation, gynecology, and others. Pharmacology studies revealed the effects of *Valeriana*, including sedative, hypnotic, antispasmodic, analgesic, antidepressant, anxiolytic, anticonvulsant, antiepileptic, neuroprotective, antibacterial, antiviral, cytotoxic, and antitumor effects as well as cardiovascular and cerebrovascular system improvements. More than 800 compounds have been isolated or identified from *Valeriana*, including iridoids, lignans, flavonoids, sesquiterpenoids, alkaloids, and essential oils. Constituents with neuroprotective, anti-inflammatory, cytotoxic, and sedative activities were also identified. However, at present, the developed drugs from *Valeriana* are far from sufficient. We further discussed the pharmacological effects, effective constituents, and mechanisms directly related to the traditional clinical applications of *Valeriana*, revealing that only several species and their essential oils were well developed to treat insomnia. To effectively promote the utilization of resources, more *Valeriana* species as well as their different medicinal parts should be the focus of future related studies. Clinical studies should be performed based on the traditional efficacies of *Valeriana* to facilitate their use in treating diseases of nervous, cardiovascular, and digestive systems, inflammation, and gynecology. Future studies should also focus on developing effective fractions or active compounds of *Valeriana* into new drugs to treat diseases associated with neurodegeneration, cardiovascular, and cerebrovascular, inflammation and tumors. Our review will promote the development and utilization of potential drugs in *Valeriana* and avoid wasting their medicinal resources.

## 1 Introduction

*Valeriana* L*.* is a group of perennial herbs, belonging to the family *Caprifoliaceae*, of which roots and rhizomes are used as medicines. Their roots and rhizomes give off special aroma and a slightly bitter taste (Zhu, 1984; Xi et al., 2002). At present, more than 200 species of *Valeriana* have been found all over the world (Du and Wu, 2006). Most wild or cultivated species are distributed in Germany, Holland, France, Belgium, Eastern Europe, India, Japan, Mexico, China and the United States (Ortiz et al., 1999; Raal et al., 2008; Sharma et al., 2012). However, only a few species have been widely reported in medicinal studies or clinical applications. For example, *Valeriana officinalis* L (*V. officinalis*) has been used in Europe and the United States for a long time to treat mild and moderate insomnia (Huang et al., 2004). There are 17 species and two varieties of *Valeriana* in China, most of which are distributed in regions ranging from northeast to southwest (Chinese Academy of Sciences, 2004). Among them, species of *V. officinalis*, *Valeriana amurensis* P. Smirn. ex Kom. (*V. amurensis*), *Valeriana jatamansi* Jones (*V. jatamansi*), *Valeriana hardwickii* Wall. (*V. hardwickii*), *Valeriana alternifolia* Bunge (*V. alternifolia*), and *Valeriana fauriei* Briq. (*V. fauriei*) have been used as the Chinese materia medica of Rhizoma et Radix Valerianae (RERV) (Ming and Guo, 1993; Castillo et al., 2000). These species were subjected to more studies due to their abundant resources (Huang et al., 2004; Zhang et al., 2006). In contrast, *V. officinalis*, *Valeriana wallichii* DC. (*V. wallichii*), and *Valeriana edulis* Nutt. (*V. edulis*) are the main medicinal species used in other countries or regions (Zuo et al., 2010; Huynh et al., 2013).

As resources of RERV, the roots and rhizomes of *Valeriana* have a long history in treating nervous system diseases in China (Tan et al., 2007). *V. jatamansi* was recorded in the *Compendium of Materia Medica* written by Li Shizhen in Ming Dynasty of China (Li, 1991). It is mainly used to treat neurasthenia, insomnia, hysteria, emotional disorders, palpitation, abdominal pain, lumbocrural pain, rheumatism pain, and dysmenorrhea. It is most commonly used for the treatment of insomnia (Duan et al., 2008; Zhou et al., 2008). The people of ancient Greece and Rome used the underground parts of *Valeriana* as a sedative approximately 1,000 years ago (Houghton, 1988). In recent years, an increasing number of pharmacological investigations on the effects of *Valeriana* have been reported, revealing the sedative, hypnotic (Valle-Mojica et al., 2011), antispasmodic, analgesic (Wei et al., 2016), antidepressant, anxiolytic (Andreatini et al., 2010), anticonvulsant, antiepileptic (Eadie, 2010), neuroprotective (Malva, 2004), antibacterial, antiviral (Rawat et al., 2017), cytotoxic, and antitumor effects (Yang et al., 2017) as well as cardiovascular and cerebrovascular system improvement (Xue et al., 2005; Chen et al., 2015). Chemical studies on *Valeriana* have identified constituents of iridoids (Wang F. F. et al., 2019), lignans (Zuo et al., 2018), flavonoids (Jugran et al., 2016), sesquiterpenes (Wang et al., 2009b), alkaloids (Torssell et al., 1967), essential oils (Rawat et al., 2017), and others. Although the efficacies, effective constituents and therapeutic mechanisms of *Valeriana* have been studied extensively and deeply, at present, only the sedative effect of *Valeriana* has been developed to treat insomnia clinically (Shinjyo et al., 2020). Obviously, the potential of *Valeriana* were not well utilized, resulting in a large waste of its resources. To promote the development and utilization of *Valeriana*, this review systematically summarizes the traditional clinical applications, bioactivities, and effective constituents of *Valeriana* as well as their relevance.

**FIGURE 1:**
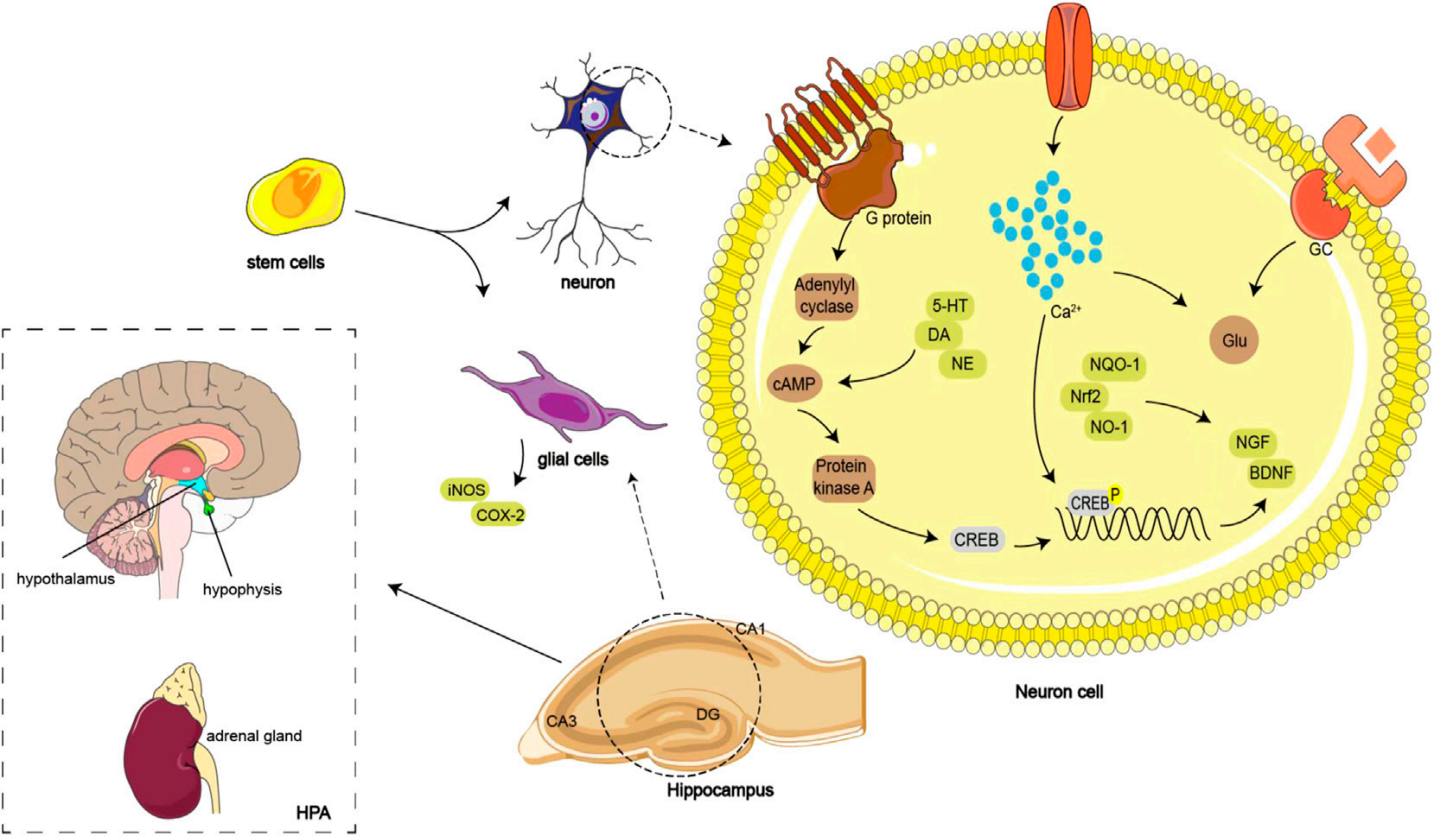
Mechanisms of antidepressant effect of *Valeriana*: promoting the level of 5-HT, the proliferation of hippocampal neurons, and the expression of phosphorylated cAMP responsive element-binding protein. Inhibiting serum corticosterone and glutamate levels, reducing glucocorticoid elevation, and stabilizes the HPA.

**FIGURE 2:**
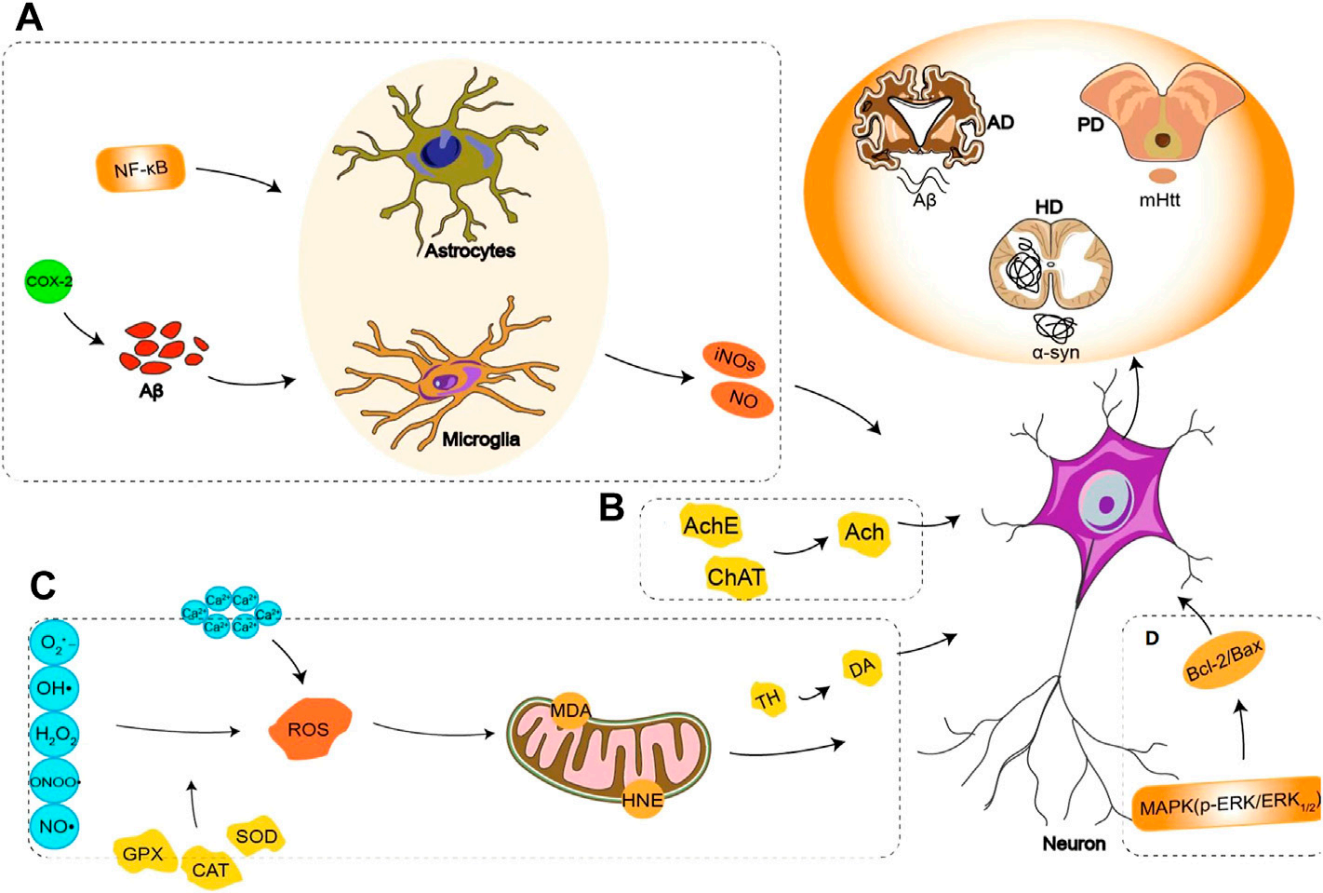
Signal pathways related to neuroprotective effect of *Valeriana*: **(A)** anti-inflammatory, inhibiting the activation of caspase-3, decreasing the activity of COX-2 and microglia and astrocytes, reducing the overexpression of iNOS and the inflammatory injury to cortical and hippocampal neurons. **(B)** Enhancing the activity of ChAT and increasing the level of Ach in the cerebral cortex and hippocampus. **(C)** Antioxidant, increasing the activities of SOD and GSH-Px and reducing the level of lipid peroxide MDA, upregulating the levels of DA and TH, increasing the number of midbrain tyrosine hydroxylase positive cells, enhancing antioxidant activity. **(D)** Anti-apoptotic, activating the p-ERK and Bcl-2 signaling pathways and inhibiting the Bax pathway to protect the brain neurons from Aβ1-42 induced apoptosis.

**FIGURE 3:**
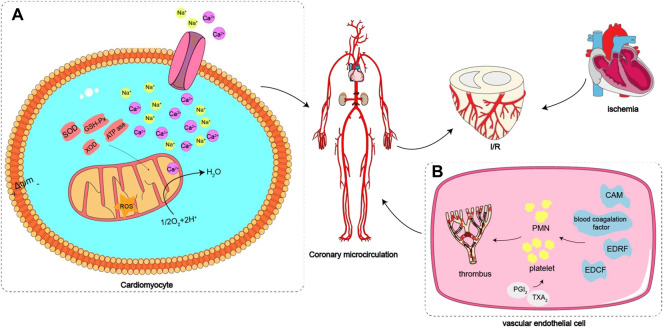
Protective effect of *Valeriana* on myocardial ischemia–reperfusion injury: **(A)** SOD, GSHPx, and ATPase activities were increased in cardiomyocytes, and Ca^2+^ levels in cardiomyocytes were remarkably reduced. **(B)** Inhibiting a variety of CAMs, blood coagulation factor and EDRF, increasing EDCF and the value of PGI2/TXA2 and inhibiting platelet aggregation to improve coronary microcirculation.

**FIGURE 4:**
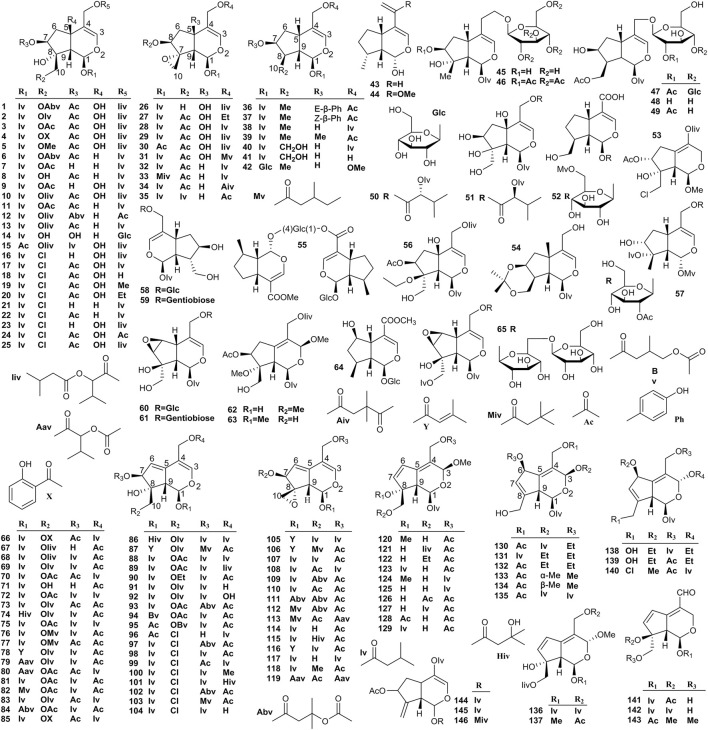
Structures of monoethenoid (**1**–**65**) and diene (**66**–**146**) iridoids from the genus *Valeriana*.

**FIGURE 5:**
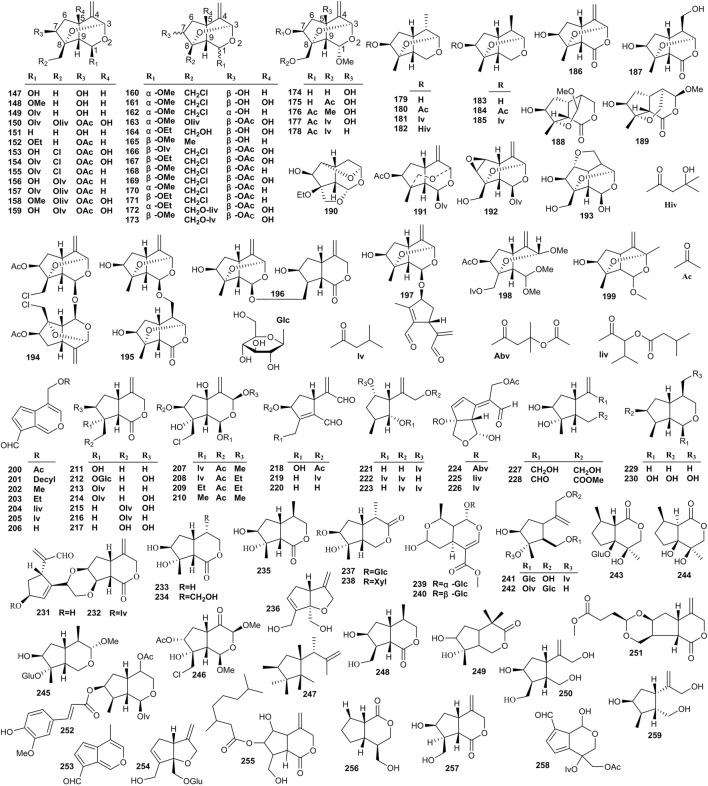
Structures of oxygen-bridge (**147–199**) and other (**200–259**) iridoids from the genus *Valeriana*.

**FIGURE 6:**
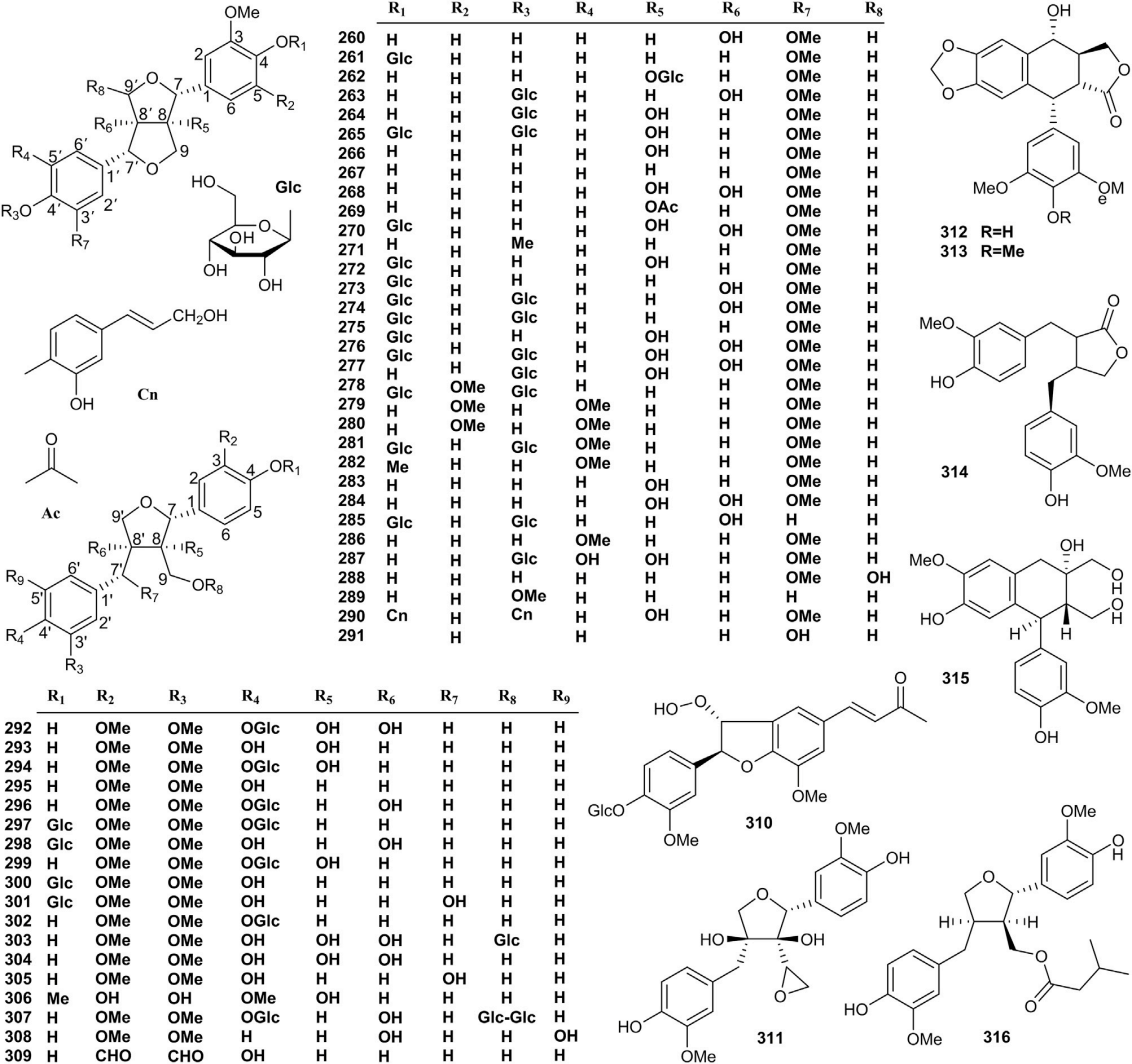
Structures of lignans from the genus *Valeriana*.

**FIGURE 7:**
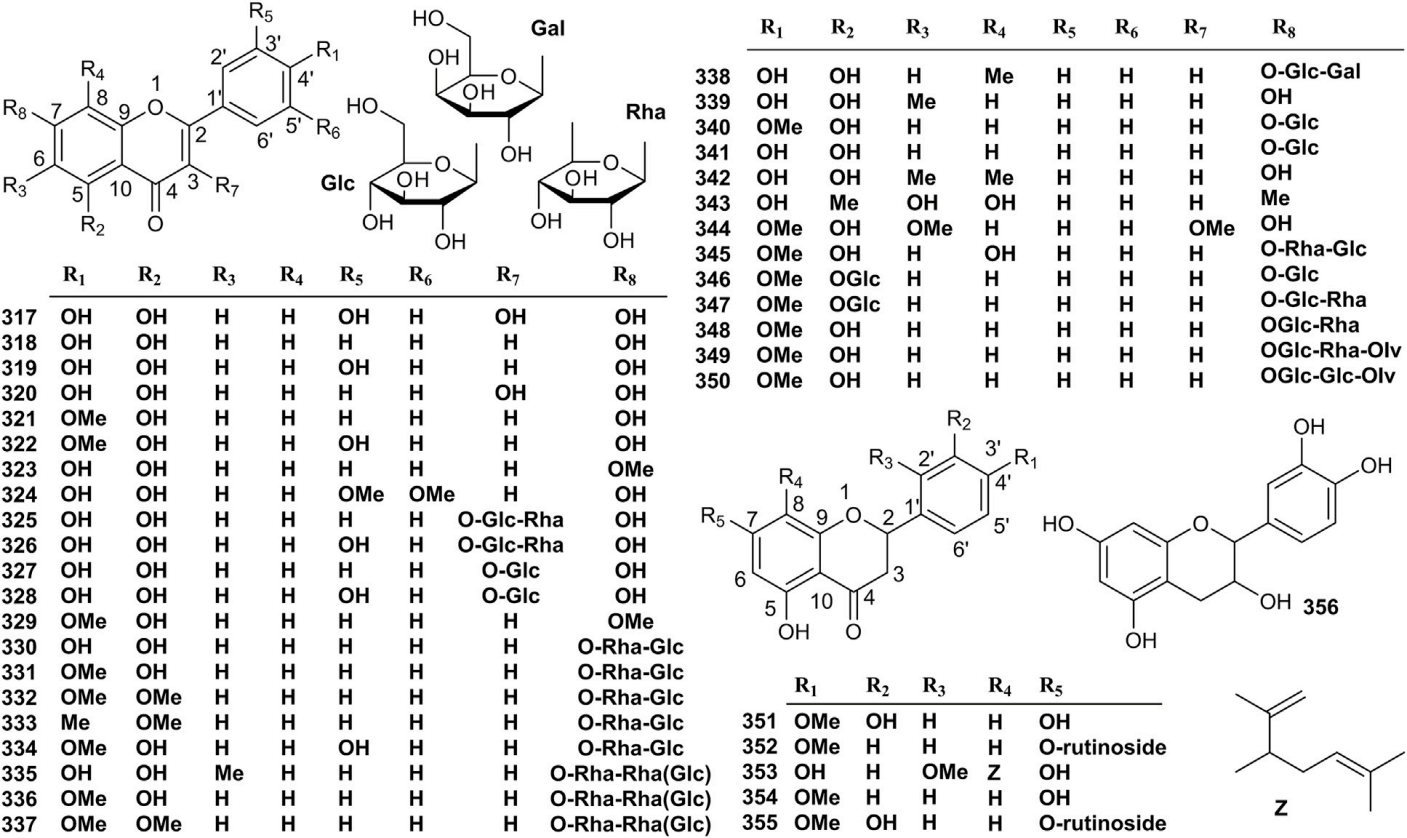
Structures of flavonoids from the genus *Valeriana*.

**FIGURE 8:**
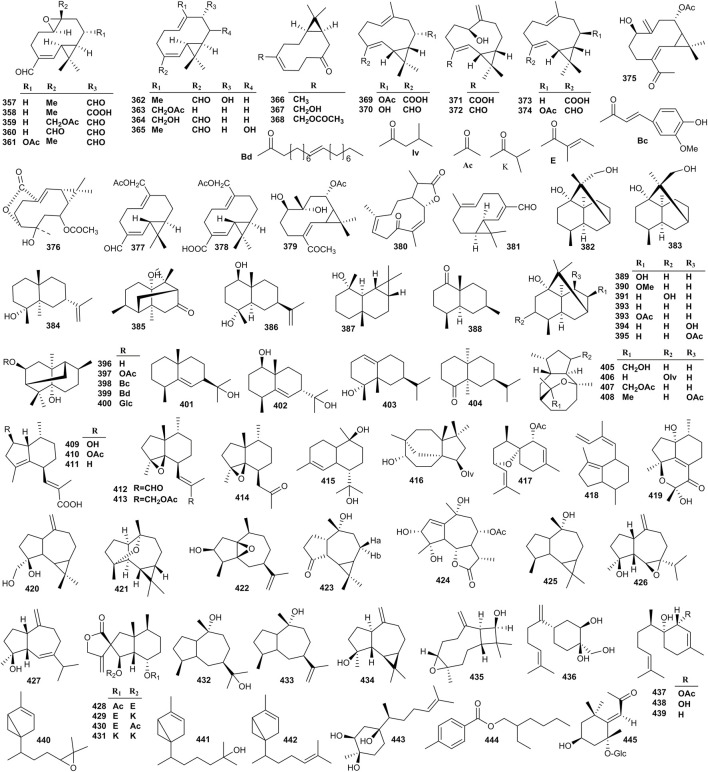
Structures of sesquiterpenoids from the genus *Valeriana*.

In this review, previous reports on *Valeriana* were searched by retrieving electronic databases of Science Direct, Web of Science, PubMed, Embase, China National Knowledge Infrastructure, Wanfang Database, China Biomedical Literature Database, VIP database, and Chinese Scientific Journals Database, as well as some books. Studies on *Valeriana* published between 1965 and 2022 were retrieved using search term “*Valeriana.*” The following terms were used in a combination for further search, which include clinical application, pharmacological effect, bioactivity, phytochemistry, active constituent, component, compound, natural product, traditional medicine, and traditional Chinese medicine. Relevant literatures were collected and irrelevant ones were removed, and then they were sorted out. The structures of compounds isolated from *Valeriana* were drawn with the software of ChemDraw 18.0 and shown in Figures 4–8. The detailed information on pharmacological studies of *Valeriana* is summarized in Table 1. The pharmacological effects and effective constituents related to traditional uses of *Valeriana* are summarized in Table 2. The detailed information on compounds and their activities is illustrated in Supplementary Tables S1–S5. The mechanisms for pharmacological effects of antidepressant, neuroprotective, and cardiovascular system improvement are elucidated in Figures 1–3, respectively. The quality of all the included studies was assessed in accordance with the best practice in research, overcoming common challenges in phytopharmacological research (Heinrich et al., 2020).

## 2 Traditional clinical applications

From ancient China to present China, the roots and rhizomes from species of *V. officinalis*, *V. amurensis*, *V. jatamansi*, *V. hardwickii*, *V. alternifolia*, and *V. fauriei* were used as the traditional Chinese medicines (TCM) of RERV (Ming and Guo, 1993; Castillo et al., 2000). RERV was used to treat anxiety, palpitations, insomnia, manic-depressive psychosis, rheumatic arthralgia, abdominal distension and pain, and dysmenorrhea (Duan et al., 2008). In modern clinical practice, RERV is mainly used as a sedative, and occasionally for the treatment of arrhythmias and spasmolysis (Oshima et al., 1995; Pakseresht et al., 2011). For instance, ethanol or aqueous extracts of RERV were prepared into capsules, tablets, tinctures, liniments, and other preparations for treating neurasthenia and insomnia. The valtrate from RERV has also been used as a sedative (Zhou and Chen, 2011). RERV combined with the fruit of *Humulus lupulus* L. (Cannabaceae) can be made into tablets. The oral administration of two tablets can reduce the arousal caused by caffeine, whereas oral administration of six tablets can inhibit arousal completely (Zhou and Chen, 2011). As a medicine for insomnia, RERV is commonly used in the clinics in combination with *Melissa officinalis* L. (Lamiaceae), *Schisandrae chinensis* (Turcz.) Baill (Schisandraceae), *Ziziphus jujuba* var. spinosa (Bunge) H.H. Hu ex H.F. Chow (Rhamnaceae) semen, and other sedative and hypnotic TCMs (Huang et al., 2007). To treat arrhythmias, RERV was also combined with extracts of *Adonis vernalis* L (Ranunculaceae) and *Crataegus* L. (Rosaceae), camphor and sodium bromide to generate a complex phytomedicine (Alexander et al., 2014). In addition, RERV was made into chewing gum to treat anxiety, syrup for the treatment of insomnia in adults and children, or combined with decaffeinated coffee tea for sedation (Yuan, 1992).

*V. jatamansi* has a long history of medicinal use in Mongols, Tibetan, Miao, Uygur, and other ethnic minorities in China. In addition to treating insomnia, the water decoction of *V. jatamansi* can be used for treating urticaria, hepatitis, mosquito bites, and headaches caused by cold (Huang et al., 2006; Guan et al., 2021). Other studies have also revealed the therapeutic effects of *V. jatamansi* on treating peptic ulcers, anemia, abdominal distention, ephidrosis, irritability, and hyperactivity (Xu, 2006). The *Dictionary of Traditional Chinese Medicine* recorded the efficacies of *V. jatamansi*, which was used to treat abdominal distension and pain, vomiting and diarrhea, pulmonary edema, menoxenia, and tuberculosis cough (Miao et al., 2017). According to the *Pharmacopoeia of the People’s Republic of China* (2020 Edition), the roots and rhizomes of *V. jatamansi* were used to treat abdominal distension and pain, indigestion, diarrhea and dysentery, rheumatism arthralgia, soreness and weakness of waist and knees, and insomnia (Chinese Pharmacopoeia Committee of People’s Repulic of China, 2020). In contrast, *TCM Resources of China* and *Northeast Medicinal Plants* and *Local Medicines of Heilongjiang* reported that *V. amurensis* was primarily used for treating nervous system diseases such as insomnia, neurasthenia, anxiety, hysteria, and epilepsy (Zhu, 1989; Zeng and Zeng, 1994; Yu et al., 1995). As early as ancient Greece and Rome, *V. officinalis* had been widely used as a mild sedative in Europe and was finally recorded in the *European Pharmacopoeia* in 1983. To date, *V. officinalis* and its extracts are widely used to treat mild and moderate insomnia in European and American countries (Zhao et al., 2011; Shinjyo et al., 2020). Preparations of *V. officinalis* have been accepted by the pharmacopoeias of more than 20 countries, including the Netherlands, Germany, and the United States (Wang R. J. et al., 2016). In 1985, the German Commission E monograph suggested preparing *V. officinalis* into tincture or infusion for relieving restlessness and nervous disturbance of sleep and defined its effects as calming and sleep-inducing (Bisset et al., 2004). As a mild sedative, *V. officinalis* was used in at least 25 sedative and hypnotic products in the United Kingdom and 400 other similar products in Germany (Houghton, 1999). Compared with plant raw materials and specific constituents of *V. officinalis*, extracts were more commonly used in these products. In addition, a double-blind experiment of 100 female students showed that *V. officinalis* effectively alleviated the pain of patients with dysmenorrhea (Mirabi et al., 2011). The essential oil of *V. officinalis* effectively treated coronary heart disease by significantly improving the symptoms of angina pectoris and myocardial ischemia in patients (Yang and Wang, 1994). In 2011, *V. hardwickii* was reported as a useful medicine, given its antiepileptic, sedative, diuretic, emmenagogue, spasmolytic, and antidiarrheal effects, but more other details were not found in Bashir et al. (2011).

## 3 Bioactivities

### 3.1 Sedative and hypnotic

Both the aqueous and ethanol extracts from *Valeriana* were effective in inducing sleep and sedative. These extracts alleviated anxiety without causing drowsiness (Bisset et al., 2004). Normal mice were administered different doses of *V. jatamansi* aqueous extract by intraperitoneal and gavage, separately, and activity changes at 30 and 60 min after administration were recorded by observing the activity time and the number of forelimb lifting in 2 min. As a result, the aqueous extract of *V. jatamansi* at doses of 2.78 (intraperitoneal) and 55.6 g/kg (gavage) significantly inhibited the autonomic activity of mice (Cao and Hong, 1994). Further studies revealed that the essential oil and iridoids fractions were responsible for the sedative effect of *V. jatamansi* (Wagner et al., 1980; Hendriks et al., 1981). *V. officinalis* aqueous extract inhibits the excitement of the cerebral cortex to reduce reflex excitability and smooth muscle spasm (Nam et al., 2013). The essential oil was determined as the effective fraction of *V. officinalis* that inhibits the autonomic activities of mice and strengthening the inhibitory effect of sodium pentobarbital and chloral hydrate on the central nervous system (CNS) (Xu et al., 1997). The mechanism of sedative and hypnotic effects of *V. officinalis* is potentially associated with increased expression of interleukin-1β (IL-1β) and tumor necrosis factor-*α* (TNF-α) (Zhang J. P. et al., 2010). Other sedative and hypnotic components of *V. officinalis* include valerianone, flavonoids, and valeric acid. These compounds could act synergistically on γ-aminobutyric acid (GABA) receptors in the cerebral cortex, promote GABA release, and inhibit the combination of GABA with receptors to regulate the function of the CNS (Ortiz et al., 1999; Marder et al., 2003). The 70% ethanol extract of *V. officinalis* reduced the sleeping latency and increased the δ-wave activity of non-rapid eye movement, demonstrating that *V. officinalis* extract could not only induce sleep but also improve sleep quality (Shinomiya et al., 2005; Tokunaga et al., 2007). In terms of promoting sleep, the petroleum ether extract of *V. amurensis* roots and rhizomes showed a similar effect as *V. officinalis* but markedly increased the levels of the neurotransmitters GABA and 5-hydroxytryptamine (5-HT) in mouse brain tissue. These results indicate that the improving sleep effect of *V. amurensis* was due to regulation of the levels of neurotransmitters in the brain (Chen J. S. et al., 2013). The detailed information of sedative and hypnotic effects for *Valeriana* is summarized in Table 1.

### 3.2 Antispasmodic and analgesic

The essential oil of *Valeriana* not only showed effectiveness in calming and helping sleep but also exhibited significant antispasmodic and analgesic effects (Wang X. et al., 2019), such as the ethanol extract of *V. wallichii*. In the ileum test of guinea pigs, *V. wallichii* exhibited a more powerful antispasmodic effect than papaverine (Wagner and Jurcic, 1979). Similarly, methanol aqueous extract from *V. hardwickii* rhizome relaxed the spontaneous contractions on an isolated rabbit jejunum. The antispasmodic effect of *V. hardwickii* was verified to be related to its iridoids, which alleviated the contracture of smooth muscle cells (Gu et al., 2004; Gilani et al., 2005, 2007). In contrast, the antispasmodic effect of chloroform and aqueous extracts from *V. jatamansi* was verified by experiments of the rabbit jejunum, rabbit aorta, and guinea pig ileum, which might be attributed to the activation of K^+^-ATP channels (Chen et al., 2012). The analgesic effect of the *V. jatamansi* aqueous extract was evaluated with a hot plate, radiation hot stimulation, and double forearm experiments. As a result, the aqueous extract of *V. jatamansi* displayed a noticeable analgesic effect and demonstrated that strong polar constituents were the effective components (Mao et al., 2008). The peripheral analgesic effect of essential oil from *V. jatamansi* was demonstrated through acetic acid-induced writhing and tail flicking experiments in mice, and the effect was attributed to the essential oil inhibiting the prostaglandin synthesis (Sah et al., 2010a).

### 3.3 Antidepressant and anxiolytic

The aqueous extract of *V. officinalis* was capable of improving the behavioral activities of depressive model rats by promoting the level of 5-HT, the proliferation of hippocampal neurons, and the expression of phosphorylated cyclic adenosine monophosphate (cAMP) responsive element-binding protein (Tang et al., 2008a; Tang et al., 2008b; Zhou et al., 2010). The antidepressant effect of *V. officinalis* aqueous extract was also reflected in its improvement of the depressive behavior of ovalbumin-sensitized rats (Hosseini et al., 2014). Further study confirmed that the antidepressant effect of *V. officinalis* aqueous extract was due to promoting the proliferation of neural stem cells and reducing the production of caspase-3 positive neurons (Qin et al., 2009). Total iridoids of *V. jatamansi* effectively improved the sucrose preference, immobility time, and depression-like behaviors of the depressive mouse model induced by chronic unpredictable mild stress, as well as the levels of 5-HT, norepinephrine (NE), substance P, and corticotropin-releasing factor (CRF) expression in the hippocampus and colon. Therefore, the antidepressant effect of total iridoids from *V. jatamansi* was involved in the brain–gut axis. Further analysis of the metabolic markers in depressive mice revealed that the antidepressive effect of the total iridoids from *V. jatamansi* was related to the metabolic pathways of the tricarboxylic acid cycle, the synthesis of neurotransmitters, and amino acid metabolism (Wang L. W. et al., 2019; Li et al., 2020a, 2020b). A dichloromethane extract from roots and rhizomes of *V. wallichii* at 40 g/kg markedly decreased the immobility period of depressive mice, and the effect was involved in the extract increasing the NE and dopamine levels in the forebrain (Sah et al., 2011b). The same experiment was performed on the essential oil from the roots and rhizomes of *V. wallichii*. However, *V. wallichii* essential oil could not only increase the level of norepine in depressive mice but could also remarkably increas the level of serotonin in the forebrain, which was attributed to the inhibition of the NO signaling pathway (Sah et al., 2011c). Another study showed that the ethanol extract of *V. fauriei* roots could reduce the immobility time of chronic restraint stress-induced depressive mice by downregulating the level of corticosterone in serum. A more in-depth study found that the ethanol extract of *V. fauriei* improved the expression of C-Fos, p-p38, cyclooxygenase-2 (COX-2), and iNOS and microglial activation in the prefrontal cortex, hippocampus, and amygdala of depressive mice. In addition, the ethanol extract of *V. fauriei* strengthened the stimulation of nuclear factor erythroid 2-related factor 2 pathways and upregulated the expression of brain-derived neurotrophic factor (BDNF). Therefore, the antidepressant effect of *V. fauriei* was associated with anti-inflammatory and antioxidant activities (Choi et al., 2018). The mechanisms of the antidepressant effect of *Valeriana* are shown in Figure 1.

In the plus maze test, the 50% ethanol extract of *V. officinalis* roots improved the anxiety behavior of rats. Specifically, rats administered the 50% ethanol extract of *V. officinalis* roots, which spent more time on the open arms. Valerenic acid present in the 50% ethanol extract was the primary anxiolytic constituent (Murphy et al., 2010). Similarly, in the dark/light preference tank test, the 50% ethanol extract of *V. officinalis* roots increased the residence time of zebrafish on the white side, which was attributed to its interaction with metabotropic glutamate receptors of zebrafish (Valle-Mojica and Ortíz, 2012). The 95% ethanol extract of *V. jatamansi* markedly increased the percentages of anxiety model rats in open arm testing by reducing blood β-endorphin and corticosterone levels. Therefore, the anxiolytic effect of *V. jatamansi* was due to the regulation of hypothalamic pituitary adrenal axis dysfunction (Yan et al., 2010; Zhao et al., 2021). The detailed information of antidepressant and anxiolytic effects for *Valeriana* is summarized in Table 1.

### 3.4 Anticonvulsant and antiepileptic

At present, it is generally believed that the anticonvulsant and antiepileptic effects of *Valeriana* are closely related to its regulation of GABA levels in the brain (Zhang et al., 2014). The aqueous extract of *V. officinalis* roots not only increased the sleeping time of pelltobarbitalum natricum-treated mice but also reduced the times of forelimb lifting, increased the seizure threshold, and prolonged the seizure latency of convulsions induced by pentylenetetrazole (PTZ). PTZ is a GABA receptor antagonist that interacts with GABA receptor to induce convulsions. Therefore, the anticonvulsant effect of *V. officinalis* is due to an increase in the levels of GABA neurotransmitters in convulsion mice (Wu et al., 2005; Nouri and Abad, 2011). Another similar study further confirmed that the aqueous extract of *V. officinalis* inhibited the absorption of [^3^H] GABA and promoted its release, which led to an increase in GABA concentration in synapses (Santos et al., 1994). The aqueous extract of *V. jatamansi* enhanced the sedative and hypnotic effects of pelltobarbitalum natricum on mice, which led to inhibition of spontaneous activity and writhing in mice. In addition to prolonging the latency period of seizures induced by picrotoxin, the aqueous extract of *V. jatamansi* resisted the convulsions induced by thiosemicarbazide (TSZ). TSZ is an inhibitor of GABA synthetase (glutamic acid decarboxylase), which leads to a decrease in GABA levels in the brain to induce convulsions. As a GABA receptor antagonist, picrotoxin interacts with the GABA receptor to induce convulsions. Therefore, the anticonvulsant effect of aqueous extract from *V. jatamansi* might be associated with the increase in GABA levels in the convulsion mouse model (Cao and Hong, 1994).

The essential oil from *V. officinalis* gradually alleviated the convulsive state of epileptic rats caused by PTZ. Moreover, the increased levels of GABA and decreased levels of glutamate in the hippocampus of epileptic rats indicated that the antiepileptic effect of the essential oil from *V. officinalis* was attributed to regulating the balance of excitatory and inhibitory amino acids in the brains of epileptic rats (Wu et al., 2008). Total iridoids of *V. officinalis* roots and rhizomes reduced the number of seizures of PTZ-induced epileptic rats, reduced the seizure time, and noticeably reduced the expression of the GABA uptake transporter GAT-1 in the hippocampus of epileptic rats. Therefore, the antiepileptic effect of total iridoids of *V. officinalis* might be related to enhancing the effect of GABA by inhibiting GAT-1 activity (Luo et al., 2004, 2005). The aqueous extract of *V. officinalis* roots reduced the discharge duration and duration of stage 5 seizures, prolonged latency to the onset of bilateral forelimb clonuses, and reduced seizure activity in an amygdala-kindled temporal lobe epilepsy rat model induced by 8-cyclopenthyl-1,3-dimethylxanthine. Moreover, the anticonvulsant effect of *V. officinalis* was decreased by 8-cyclopenthyl-1,3-dimethylxanthine, which is a selective adenosine A_1_ receptor antagonist. Therefore, the anticonvulsant effect of *V. officinalis* might be due to the activation of the adenosine system (Rezvani et al., 2010). The detailed information of anticonvulsant and antiepileptic effects for *Valeriana* is summarized in Table 1.

### 3.5 Neuroprotective

*Valeriana* can be used to treat nervous system diseases, such as PD, Alzheimer’s disease (AD), and Huntington’s disease, which are mostly related to brain neuron injuries or apoptosis. Therefore, some scholars have carried out studies on the neuroprotective effect of *Valeriana* in recent years. The ethanol extract of *V. officinalis* significantly improved the learning, memory, and autonomous activities of AD model rats. On the one hand, *V. officinalis* increased the level of catalase and total antioxidative capacity (T-AOC) in serum and reduced the activity of acetylcholinesterase. On the other hand, *V. officinalis* increased the activities of superoxide dismutase (SOD) and glutathione peroxidase (GSH-Px) and reduced the level of lipid peroxide malondialdehyde (MDA). Therefore, the neuroprotective effect of *V. officinalis* should be involved in improving the function of the cholinergic nervous system and antioxidation (Yoo et al., 2015; Zhang and Zuo, 2018). The neuroprotective effect of the 70% ethanol extract from *V. officinalis* at 10 ng/ml–100 μg/ml was evaluated on Aβ_25–35_-induced hippocampal neuronal toxicity in rats, and the decrease in neuronal cell viability and number in the brain was prevented. The mechanisms involved inhibiting excess influx of Ca^2+^ following neuronal injury and partially inhibited ascorbate/iron-induced peroxidation (Malva et al., 2004). In addition, experiments on *Drosophila melanogaster* showed a protective effect of the aqueous extract from *V. officinalis* roots at 10 mg/ml on 500 mM rotenone-induced neuronal toxicity, and the restored expression of SOD and catalase mRNA confirmed that *V. officinalis* could represent a potential therapeutic strategy for neurodegenerative diseases, including PD (Sudati et al., 2013). Iridoids from *V. jatamansi* showed a significant protective effect against MPP^+^-induced SH-SY5Y cell death (Xu et al., 2012c). Using a 1-methyl-4-phenyl-1,2,3,6-tetrahydropyridine (PMTP) induced mouse PD model to evaluate the neuroprotective effect of *V. wallichii*, the results showed that the 50% methanol extract of *V. wallichii* upregulated the levels of dopamine and tyrosine hydroxylase, increased the number of midbrain tyrosine hydroxylase positive cells, enhanced antioxidant activity, reduced reactive oxygen species (ROS), lipid peroxidation (LPO), and inflammatory cytokines (Subhashree et al., 2015). The 50% ethanol eluted fraction (in macroporous adsorption resin column chromatography) from a 95% ethanol extract of *V. amurensis* roots and rhizomes could effectively inhibit the overexpression of β-APP- and Aβ_1–40_-positive cells in brain neurons of the AD rat model and prevent β-APP and Aβ_1–40_ aggregation in the brain. This fraction reduced the apoptosis or injury of neurons in the brain by inhibiting the activation of caspase-3, decreasing the activity of COX-2 and microglia and astrocytes and reducing the overexpression of inducible nitric oxide synthase (iNOS) and the inflammatory injury to cortical and hippocampal neurons to improve spatial exploration memory in AD rats (Zhang Z. L. et al., 2010; Janaína et al., 2018). The neuroprotective mechanism of *V. amurensis* was also examined in an AD mouse model established by intrahippocampal injection of Aβ_1–42_ in our previous study. The results showed that the 50% ethanol extract of *V. amurensis* roots and rhizomes remarkably improved cognitive function in AD mice by enhancing the activity of choline acetyltransferase and increasing the level of acetylcholine in the cerebral cortex and hippocampus of mice. The extract of *V. amurensis* also activated the p-ERK and Bcl-2 signaling pathways and inhibited the Bax pathway to protect brain neurons from Aβ_1–42_-induced apoptosis (Wang et al., 2013). According to our previous study, the lignans in *V. amurensis* might be responsible for the neuroprotective activity (Wang et al., 2021). Other experiments demonstrated that valeric acid in *V. wallichii* essential oil was similar to the structure of the neurotransmitter GABA, which could noticeably improve experimental dementia (Vishwakarma et al., 2016). In addition, *V. officinalis* tincture effectively reduced the volume of cerebral infarction, the density of C-Fos- and C-Jun-positive cells in the hippocampus and the degree of neuronal damage in the cerebral cortex in rats with experimental reversible middle cerebral artery occlusion. Therefore, the effect of *V. officinalis* on focal cerebral ischemia was associated with the inhibition of the expression of C-Fos and C-Jun in the hippocampus (Wang et al., 2004). The signaling pathways related to the neuroprotective effect of *Valeriana* are shown in Figure 2, and the detailed information of neuroprotective effects for *Valeriana* is summarized in Table 1.

### 3.6 Cardiovascular and cerebrovascular system improvement

The effects of *Valeriana* on the cardiovascular and cerebrovascular systems include myocardial protection, antiarrhythmia, and anticerebral ischemia**-**reperfusion injury. *V. officinalis* aqueous extract noticeably alleviated myocardial spasm in an isolated cardiac ischemia–reperfusion rat model, which resulted in more regular, powerful, and smooth myocardium contraction and relaxation. Further study revealed that *V. officinalis* reduced the activity of lactic dehydrogenase, phosphochain kinase (CK), and the levels of MDA. In contrast, SOD, GSHPx, and adenosine triphosphatase activities were increased in cardiomyocytes, and Ca^2+^ levels in cardiomyocytes were remarkably reduced. Therefore, the protective effect of *V. officinalis* aqueous extract on myocardial ischemia-reperfusion injury was closely related to the decrease in Ca^2+^ concentration in cardiomyocytes and antilipid peroxidation (Yang et al., 2012). Another study showed that the protective effect of *V. officinalis* on myocardial ischemia–reperfusion injury might occur through inhibiting xanthine oxidase, reducing the production of free radicals, increasing the value of prostacyclin 2/thromboxane A_2_, inhibiting platelet aggregation, improving coronary microcirculation, decreasing TNF-α production, and reducing aseptic inflammation in the reperfusion area to alleviate myocardial ischemia–reperfusion injury (Yin et al., 2000). The ethanol extract of *V. officinalis* significantly slowed the heart rate and reduced the blood pressure and the partial pressure ratio of arteriovenous blood oxygen in anaesthetized cats, confirming the effects of reducing myocardial oxygen consumption and expanding coronary vessels (Zhang et al., 1982). The protective effect of *Valeriana* on myocardial ischemia–reperfusion injury is shown in Figure 3. The aqueous and n-butanol extracts of *V. officinalis* remarkably delayed the occurrence time of ventricular extrasystole and ventricular fibrillation (VF) induced by aconitine in rats and reduced the incidence of VF. In contrast, the essential oil and ethyl acetate extracts of *V. officinalis* significantly reduced the incidence of VF induced by chloroform in mice. The arrhythmia induced by aconitine in rats is caused by exciting the myocardium directly, opening the myocardial Na^+^ channel, and accelerating the Na^+^ influx of cardiomyocytes. Chloroform-induced VF in mice is related to the induction of adrenal medulla to secrete adrenaline to activate *β* receptors. Therefore, the antiarrhythmic effect of *V. officinalis* was associated with the inhibition of Na^+^ influx and blocking of cardiomyocyte *β* receptors (Wen et al., 2009). The essential oil from *V. officinalis* increased the uptake of technetium-99 m ethyl cysteinate dimer in mouse brain cells, brain radiation count and brain blood ratio to improve the microcirculation perfusion of mice brain tissue. *V. officinalis* essential oil also exhibited an antagonistic effect on acute cerebral ischemia caused by NE and significantly improved the blood supply insufficiency of brain cells. The mechanism might involve relieving arterial spasm, increasing cerebral blood flow, inhibiting platelet aggregation, and improving microcirculation (Li et al., 2004).

The essential oil of *V. officinalis* inhibited the contraction of rat vascular smooth muscle cells induced by angiotensin II, which confirmed the effect of dilating blood vessels to reduce blood pressure. The effect could not be blocked by the NOS inhibitor N′-nitro-L-arginine-methylesterhydrochloride, indicating that the inhibitory effect of the essential oil of *V. officinalis* on vascular smooth muscle cell contraction was not due to endogenous NO (Yang et al., 2002). The aqueous extract from *V. officinalis* accelerated the peak systolic velocity of the basilar artery and increased the diameter of the basilar artery after subarachnoid hemorrhage in rabbits, which dilated the spastic cerebral artery and reduced cerebral vasospasm effectively. The effect might be involved in reducing the damage to vascular endothelial cells by scavenging free radicals, inhibiting inflammation, preventing platelet, and leukocyte aggregation and adhesion, and inhibiting the immune response (Luo et al., 2001). The essential oil of *V. officinalis* shortened the duration of arrhythmia in rats caused by BaCl_2_, reduced its incidence, antagonized arrhythmia in mice induced by CaCl_2_, and reduced the number of mouse deaths, suggesting that *V. officinalis* plays an antiarrhythmic role as a Ca^2+^ antagonist (Chen and Yu, 1990). The detailed information of cardiovascular and cerebrovascular system improvements for *Valeriana* is summarized in Table 1.

**TABLE 1:** Summary of pharmacology effects for *Valeriana*.

Bioactivity	Source and extract	Model used	Positive drug and dose (administration)	Dose (administration) and duration	Minimum effective concentration	Molecular mechanism or outcome
Sedative and hypnotic	*V. jatamansi* roots and rhizomes, aqueous extract	*In vivo* normal mice	―	13.9, 27.8, and 55.6 g/kg (i.g.)	13.9 g/kg	Activity time, the number of forelimb lifting **↓**
	*V. officinalis* roots and rhizomes, aqueous extract	*In vitro* normal mice	Diazepam, 4 mg/kg (i.g.)	1, 3, and 5 g/kg (i.g.), 1 week	1 g/kg	IL-1β and TNF-α**↑**
	*V. amurensis* roots and rhizomes, petroleum ether extract	*In vivo* pentobarbital sodium induced sleep in mice	Diazepam, 2.5 mg/kg (i.g.)	7.5, 15, and 30 g/kg (i.g.), 2 weeks	7.5 g/kg	GABA and 5-HT **↑**
Antidepressant and anxiolytic	*V. officinalis* roots and rhizomes, aqueous extract	*In vivo*, CMS rats	Fluoxetine, 2.2 mg/kg (i.g.)	100, 200, and 400 mg/kg (i.g.), 3 weeks	100 mg/kg	5-HT, cell in the hippocampus **↑**
	*V. jatamansi* roots and rhizomes, total iridoids	*In vivo*, CUMS rats	Fluoxetine, 2.6 mg/kg (i.g.)	5.7, 11.4, and 22.9 mg/kg (i.g.), 1 week	5.7 mg/kg	5-HT and NE **↑;** SP and CRF **↓**
	*V. wallichii* roots and rhizomes, dichloromethane extract	*In vivo*, FST mice	Imipramine, 10 mg/kg (i.g.)	10, 20, and 40 mg/kg (i.g.), 2 weeks	20 mg/kg	NE and DA **↑**
	*V. fauriei* roots and rhizomes, ethanol extract	*In vivo*, CRS mice	―	100 and 200 mg/kg (i.g.), 2 weeks	200 mg/kg	C-Fos, p-p38, COX-2, iNOS, Nrf2, and BDNF **↑**
Anticonvulsant and antiepileptic	*V. officinalis* roots and rhizomes, aqueous extract	*In vivo*, PTZ mice	Diazepam, 5 mg/kg (i.g.)	9 g/kg (i.g.), 2 weeks	―	GABA, the threshold of PTZ seizure **↑**
	*V. jatamansi* roots and rhizomes, aqueous extract	*In vivo*, TSZ mice	Diazepam, 5 mg/kg (i.p.)	2.75, 5.5, and 11.0 g/kg (i.p.), 2 h	11.0 g/kg	GABA **↑**
	*V. officinalis* roots and rhizomes, essential oil	*In vivo*, PTZ rats	Sodium valproate, 30 mg/kg (i.p.)	30 mg/kg (i.p.), 2 weeks	30 mg/kg	GABA **↑;** Glu **↓**
	*V. officinalis* roots and rhizomes, total iridoids	*In vivo*, PTZ rats	―	0.5, 1, and 1.5 g/kg (i.g.), 4 weeks	0.5 g/kg	GABA **↑;** GAT-1 **↓**
	*V. officinalis* roots and rhizomes, aqueous extract	*In vivo*, TLC rats	―	200, 500, and 800 mg/kg (i.p.)	500 mg/kg	Latency to the onset of bilateral forelimb clonuse**s↑;** discharge duration, duration of stage five seizures **↓**
Neuroprotective	*V. officinalis* roots and rhizomes, ethanol extract	*In vivo*, AD rats	―	25 and 100 mg/kg (i.g.), 3 weeks	25 mg/kg	T-AOC, SOD, GSH-Px **↑;** MDA **↓**

	*V. wallichii* roots and rhizomes, 50% methanol extract	*In vivo*, PD mice	―	50, 100, and 200 mg/kg (i.g.), 3 weeks	100 mg/kg	DA, TH^+^ cell count, antioxidant activity **↑;** ROS, LPO, and GSH **↓**
	*V. amurensis* roots and rhizomes, the 50% ethanol eluted fraction from a 95% ethanol extract	*In vitro*, SDAD rats	*Jiannao* capsule 2.41 g/kg (i.g.)	0.26 and 0.52 g/kg (i.g.), 1 week	0.26 g/kg	Caspase-3, COX-2, microglia, and astrocytes, iNOS **↓**
	*V. amurensis* roots and rhizomes, 50% ethanol extract	*In vivo,* AD mice	Piracetam 0.5 g/kg; aricept 2 mg/kg (i.p.)	0.2, 0.4, and 0.8 g/kg (i.g.), 2 weeks	0.4 g/kg	ACh, ChAT, p-ERK, and Bcl-2 **↑;** Bax **↓**
Cardiovascular and cerebrovascular system improvements	*V. officinalis* roots and rhizomes, aqueous extract	*In vitro,* I/R rats	―	25, 50, and 100 mg/kg (i.p.), 2 h	25 mg/kg	SOD, ATP-ase, GSHPx **↑;** LDH, CK, MDA, and Ca^2+^ in cardiomyocytes **↓**
	*V. officinalis* roots and rhizomes, aqueous extract	*In vitro,* I/R rabbits	―	100 mg/kg (i.p.), 2.5 h	―	PGI_2_/TXA_2_ and coronary microcirculation **↑;** platelet aggregation, TNF-α, and aseptic inflammation **↓**
	*V. officinalis*, roots and rhizomes, essential oil and ethyl acetate extracts	*In vivo* arrhythmic rats	Propranolol, 40 mg/kg (i.p.)	50, 25, and 12.5 g/kg (i.g.), 3 h	25 g/kg	Na^+^ influx of cardiomyocytes and cardiomyocyte *β* receptors **↓**
	*V. officinalis* roots and rhizomes, essential oil	*In vivo* acute cerebral ischemia, mice	Ligustrazine, 25 mg/kg (i.g.)	200 and 300 mg/kg (i.g.), 1 h	200 mg/kg	Cerebral blood flow and microcirculation **↑;** platelet aggregation **↓**

	*V. officinalis* roots and rhizomes, aqueous extract	*In vivo,* SAH rabbits	Nimotop, 6 mg/kg (i.g.)	500 mg/kg (i.g.), 5 days	―	Free radicals, inflammation, platelet and leukocyte aggregation and adhesion, and immune response **↓**
	*V. officinalis* roots and rhizomes, essential oil	*In vivo* CaCl_2_ induced arrhythmic rats	Propranolol, 20 mg/kg (s.c.)	400 and 500 mg/kg (s.c.), 30 min	500 mg/kg	Ca^2+^ concentration **↓**

### 3.7 Antibacterial and antiviral

The total alkaloids and essential oil of *V. officinalis* showed remarkable antibacterial effects and were particularly effective against Gram-positive bacteria. *V. officinalis* essential oil exhibited broad-spectrum antibacterial activity. The minimum inhibitory concentration (MIC) values ranged from 62.5 to 400 μg/ml, whereas the IC_50_ values ranged from 36.93 to 374.72 μg/ml. *V. officinalis* essential oil also showed inhibitory activity against fungi. For example, *V. officinalis* essential oil exhibited moderate inhibitory activity against the growth of *Candida albicans* and inhibited *magnaporthe oryzae* spore germination (Wang Y. F. et al., 2010). The essential oil of *V. jatamansi* whole plants exhibited potential antibacterial activity against *Pseudomonas aeruginosa, Bacillus pumilus, Escherichia coli, Staphylococcus aureus, Candida albicans*, *and Staphylococcus epidermidis* (Agnihotri et al., 2011). The 50% ethanol extract of *V. jatamansi* showed powerful antibacterial activity against the pathogens *Micrococcus luteus*, *Escherichia coli*, *Escherichia coli mutans*, *Salmonella abony*, *Lactobacillus plantarum*, and *Staphylococcus epidermidis* and exhibited antibacterial activity against multidrug-resistant strains of *Staphylococcus aureus* and *Pseudomonas aeruginosa*. The antibacterial components of the ethanol extract were confirmed to be alkaloids (Babu et al., 2015). Both chloroform and n-hexane extracts of *V. wallichii* leaves showed noticeable antibacterial activity against *Staphylococcus aureus* and *Bacillus subtilis*, and the latter also exhibited antibacterial activity against *Bacillus subtilis* and *Microsporum canis*. Chloroform and aqueous extracts from *V. wallichii* leaves were efficient inhibitors of *Microsporum canis* and *Aspergillus flavus* (Khuda et L., 2012). In addition, the methanol extract of *V. wallichii* rhizomes inhibited HCV (Ganta et al., 2017). As a Rev-transport inhibitor, valtrate from *V*. *fauriei* showed a potential anti-HIV effect, which inhibited the P-24 production of HIV-1 virus but without any toxicity to host MT-4 cells (Murakami et al., 2002).

### 3.8 Cytotoxic and antitumor

Studies on the cytotoxic or antitumor activities of *Valeriana* have focused on their iridoid constituents. Valtrate, didrovaltrate, and baldrinal in *V. wallichi* exhibited powerful cytotoxicity to liver cancer cells. Among them, valtrate with the strongest cytotoxicity, and its activity was twice as toxic as didrovaltrate and eight times as toxic as baldrinal. Didrovaltrate had a more rapidly toxic effect on hepatoma cells. The dose-effect relationship study showed that the hepatoma cells died after 2 h of exposure to 66 μg/ml didrovaltrate, and all hepatoma cells died 5 h later (Bounthanh et al., 1981). Another study showed that didrovaltrate significantly inhibited the proliferation of Kreb’s II ascites cancer cells at a concentration of 100 mg/kg (Hude et al., 1986).

*V. jatamansi* iridoid compounds didrovaltrate acetoxyhydrin, volvaltrate B, isovaleroxyhydroxy-dihydrovaltrate, 10-acetylpatrinoside, jatamanvaltrate Z1-Z3, valtrate, and acevaltrate exhibited moderate cytotoxicity against the metastatic prostate cancer (PC-3M), hepatoma (Bel7402), lung adenocarcinoma (A549), and colon cancer (HCT-8) cell lines with IC_50_ values ranging from 1.0 to 8.5 μM (Lin et al., 2014; Xie et al., 2019). Valejatanin A displayed powerful cytotoxicity against cancer cell lines, including human colon carcinoma HT29, human leukemia K562 and mouse melanoma B16 with IC_50_ values of 22.17, 15.26, and 3.53 μg/ml, respectively. Compounds 8-acetoxypatchouli alcohol and valerol A showed moderate activities against mice melanoma B16 cell lines with IC_50_ values of 31.43 and 30.78 μg/ml, respectively (Quan L. Q. et al., 2019). Another study showed that 8,9-dihydro-7-hydroxy-dolichodial and valeridoid F were cytotoxic to three types of human glioma stem cells and inhibited their growth effectively. The IC_50_ values of 8,9-dihydro-7-hydroxy-dolichodial and valeridoid F against GSC-3 were 35.84 and 42.45 μM, respectively. The respective IC_50_ values against GSC-12 were 36.67 and 41.40 μM. The respective IC_50_ values against GSC-18 were 30.19 and 47.55 μM (Quan L. Q. et al., 2020). Iridoids of valtrate, didrovaltrate, and baldrinal could induce apoptosis of MKN-45 gastric cancer cells, which might be related to increased expression of caspase-3 and caspase-9 in MKN-45 gastric cancer cells. Further studies showed that iridoids of valtrate, didrovaltrate, and baldrinal upregulated P53 protein expression and downregulated survivin protein expression in MKN-45 gastric cancer cells (Ye et al., 2004, 2007). The valepotriate in *V. wallichii* exhibited strong toxic effects on untransformed mouse early hematopoietic progenitor cells (CFU-GM and CFU-EOS) and human peripheral blood T-lymphocytes (Tortarolo et al., 1982). The inhibitory rate of valepotriate at concentrations of 100–150 mg/kg on transplanted tumor S180 in mice was 58%–68%. In addition, the survival time of Ehrlich ascites cancer mice was prolonged by 62%–66%, and the formation of erythrocyte rosettes in mice was also increased. Histopathological observation showed that valepotriate induced flake necrosis in the center of the tumor mass, which was surrounded by a large number of lymphocytes and macrophages. Therefore, valepotriate not only exhibited a significant antitumor effect but also enhanced the immune function of mice (Zhang S. Q. et al., 2010). In addition, the total flavonoids of *V. jatamansi* remarkably reduced the average tumor weight of liver cancer H22 mice, and the tumor inhibition rate was between 25% and 31%. These effects were associated with the inhibition of the JAK/STAT signaling pathway (Yan et al., 2011).

### 3.9 Others

In addition to the aforementioned pharmacological effects, other studies also reported the hepatoprotection of *V. officinalis* (Prasad et al., 2010), anti-inflammatory effects of *V. wallichii* flavonoids and tannins (Subhan et al., 2007), antioxidant effects of *V. jatamansi* flavonoids and tannins (Thusoo et al., 2014), inhibition of acute edema by *V. jatamansi* essential oil (Agnihotri et al., 2011), anticholinesterase effects of *V. jatamansi* iridoids and sesquiterpenoids (Dong et al., 2015b), and regulation of gastrointestinal disorder of *V. jatamansi* iridoids (Jugran, et al., 2019).

## 4 Phytochemistry

### 4.1 Iridoids

At present, 259 iridoids have been identified in *Valeriana*. Iridoids are acetal derivatives of iridoidium, which are mainly divided into monoethenoids, dienes, and oxygen bridges (Wang R. J. et al., 2016). The substitution of various acyl groups on the mother nuclear structure led to the structural diversity of iridoids, such as acetyl, isovaleryl, acetoxy isovaleryl, isovaleroxylisovaleroxyl, methyisovaleroxyl, and hydroxylisovaleroxyl (Liu, 2020). Generally, the structures of monoethenoid iridoids contain only one double bond located between C-3 and C-4 (Lin et al., 2009), that is, compounds **1**–**65** (Figure 4). The structures of diene iridoids contain two double bonds located between C-3 and C-4 and between C-5 and C-6, but some are located between C-4 and C-5 and between C-6 and C-7 (Xu et al., 2012a; Lin et al., 2014). The diene iridoids include compounds **66**–**146** (Figure 4).

The structures of oxygen-bridge iridoids contain an ether bond between C-3 and C-8 or between C-3 and C-10, and dioxygen bridge cage fragments exist in a minority of oxygen-bridge iridoids (Lin et al., 2010a). The oxygen-bridge iridoids include compounds **147**–**199** (Figure 5). In addition to the aforementioned three types of iridoids, other types of iridoids were also isolated from *Valeriana*. These compounds with lactone fragments formed a double bond out of the ring between C-4 and C-11. A free hydroxyl substitution typically occurs in these compounds, and relatively few acyl substituents are noted. Such compounds are formed when the A ring (five-membered ring) or B ring (six-membered ring) in the structure is split, and the substitution of hydroxyl, methoxy, ester and other groups occurs (Liu, 2020). Other iridoids include compounds **200–259** (Figure 5).

Iridoids are the main components with cytotoxic activities in *Valeriana*. Most compounds of iridoids were separated from the whole plants or roots and rhizomes of *V. jatamansi*, *V. officinalis*, *V. amurensis*, *V. dioscoridis*, and *V. sorbifolia*, such as jatamanvaltrate A-H (**1**–**8**), didrovaltrate acetoxyhydrin (**11**) (Wang et al., 2009a; Lin et al., 2009; Yu et al., 2010; Liu et al., 2021), chlorovaltrate E-K (**16**–**22**) (Lin et al., 2013), jatamanvaltrate L (**26**) and M (**27**), 5-hydroxydidrovaltrate (**28**), and didrovaltrate (**32**) (Thies, 1968b; Lin et al., 2009). Among them the diene iridoids showed more powerful cytotoxic activity on some tumor cell lines; for example, compounds, such as chlorovaltrate (**98**), rupesin B (**99**), valtrate (**107**), and acevaltrate (**109**) showed cytotoxic activity against metastatic prostate cancer, lung adenocarcinoma, hepatoma, and colon cancer cell lines (Lin et al., 2009; 2010b, 2013).

Thies et al. (1968a) isolated and identified valtrate (**107**, also known as valepotriate) from *Valeriana* for the first time and preliminarily demonstrated its sedative activity (Zhang et al., 2014). Valtrate mainly exists in the roots and rhizomes of *V. jatamansi* and the whole plants of *V. officinalis*. Other compounds with sedative activity were isolated from the aerial parts or roots and rhizomes of *V. jatamansi* and *V. sorbifolia*, including isovaltrate (**108**), baldrinal (**200**), and decyl baldrinal (**201**) (Thies, 1968b; Xu et al., 2007; Su, 2016). Some iridoids from *Valeriana* also exhibit neuroprotective activities. For example, jatamanvaltrate G (**7**) and H (**8**), valeriotriate B (**9**), and valeriandoid C (**156**) exhibited moderate neuroprotective effect against the neuronal SH-SY5Y cell model induced by 1-methyl-4-phenylpyridinium (MPP^+^) (Xu et al., 2011a; 2012b). Suspensolide F (**14**), jatadoid B (**24**), patrinoside (**58**), and kanokoside A (**60**) displayed significant neuroprotective effects against PC12 cell injury induced by Aβ_25–35_ (Wang et al., 2012a; Wan et al., 2016). Patrinoside-aglucone (**41**) and stenopterin B (**54**) showed promoting effects on NGF-induced neurite outgrowth in PC12 cells (Dong et al., 2015a). In addition, (4β,8β)-8-methoxy-3-methoxy-10-methylene-2,9-dioxatricyclo [4.3.1.0] decan-4-ol (**165**) (Quan L. Q. et al., 2019), isopatrinide (**252**), and vibutinal (**253**) showed neuroprotective effects against PC12 cell death induced by CoCl_2_ (Tan et al., 2016; Wang et al., 2017).

In addition, a study showed that jatadomin A (**198**), B (**158**), C (**178**), D (**53**), E (**246**), and jatamanvaltrate Q (**89**) exhibited anti-inflammatory activities by inhibiting nitric oxide (NO) release in murine microglial BV-2 cells induced by lipopolysaccharide (LPS) (Wang H. M. et al., 2020). Similarly, the anti-inflammatory activities of jatamanvaltrate B (**2**), E (**5**), and W (**123**), valeriotetrate C (**15**), 10-isovaleroxy-valtrathydrin (**69**), and patriscadoid I (**124**) were confirmed in RAW 264.7 cells stimulated with LPS (Liu et al., 2021). The details are shown in Supplementary Table S1 of the supplementary material.

### 4.2 Lignans

In recent years, more than 57 lignans have been isolated from *Valeriana*, which are divided into types of furofurans (**260–291**), tetrahydrofurans (**292–309, 311**, and **316**), and others (**310** and **312–315**). The structures of lignans are shown in Figure 6. Lignans are the main components responsible for the neuroprotective effect of *Valeriana*. Specifically, furofurans lignans of pinoresinol-8-O-*β*-D-glucopyranoside (**262**), pinoresinol-4,4′-di-O-*β*-D-glucoside (**265**), 8-hydroxypinoresinol (**266**), pinoresinol (**267**), prinsepiol (**268**), prinsepiol-4-O-*β*-D-glucopyranoside (**270**), 8-hydroxypinoresinol-4,4′-di-O-*β*-D-glucopyranoside (**274**), 8,8′-di-hydroxyl-pinoresinol-4,4′-di-O-*β*-D-glucopyranoside (**277**), syringaresinol-4,4′-di-O-*β*-D-glucopyranoside (**279**), (+)-medioresinol-4,4′-di-O-*β*-D-glucopyranoside (**282)**, tetrahydrofurans lignans of olivil-4′-O-*β*-D-glucopyranoside (**296**), lariciresinol-4,4′-di-O-*β*-D-glucopyranoside (**297**), olivil-4-O-*β*-D-glucopyranoside (**298**), 8-hydroxylariciresinol-4′-O-*β*-D-glucopyranoside (**299**), lariciresinol-4-O-*β*-D-glucopyranoside (**300**), neoarctin A (**301**), lariciresinol-4′-O-*β*-D-glucopyranoside (**302**), and massoniresinol-3a-O-*β*-D-glucopyranoside (**303**) exhibited significant protective effects against amyloid-beta (Aβ)-induced neurotoxicity in PC12 cells (Wang et al., 2012a; Wang et al., 2014 C. F.; Kırmızıbekmeza et al., 2018). Compounds **266** and **268** showed powerful antioxidant activity, and **266** also exhibited an appreciable vasodilation activity (Piccinelli et al., 2004). 4′-O-*β*-D-glucosyl-9-O-(6″-deoxysaccharosyl) olivil (**307**) was demonstrated to be a partial agonist of A1 adenosine receptors in rats and humans (Schumacher et al., 2002), and the inhibitory effect of (−)-matairesinol (**314**) on NO production in RAW 264.7 macrophages induced by LPS suggested that **314** exhibited obvious anti-inflammatory effect (Xie et al., 2019), respectively. By contrast, 4′-demethylpodophyllotoxin (**312**) and podophyllotoxin (**313**) showed significant cytotoxic activity, so it is not surprising that **313** and its derivatives are extensively applied as anticancer drugs (Glaser et al., 2015).

The activities of other lignans, including 8′-hydroxypinoresinol (**260**), pinoresinol-4-O-*β*-D-glucopyranoside (**261**), 8′-hydroxypinoresinol-4′-O-*β*-D-glucopyranoside (**263**) (Schumacher et al., 2002; Wang et al., 2012b; Quan L. Q. et al., 2020), and (+)-1-acetoxypinoresinol (**269**) (Fan et al., 2020), have not been determined in a phytochemical study of *Valeriana*. The details are shown in Supplementary Table S2 of the supplementary material.

### 4.3 Flavonoids

In recent years, 40 flavonoids have been isolated from *Valeriana*. The structures of **317**–**356** are shown in Figure 7. Compound 2*S* (-)-hesperidin (**351**) showed sedative and sleep enhancing activities, whereas 6-methylapigenin (**339**) exhibited anxiolytic and sleep-enhancing properties (Marder et al., 2003). In addition, some studies have proven that acacetin (**321**) and hesperidin (**350**) are the effective constituents with neuroprotective activity both *in vitro* and *in vivo* in Parkinson’s disease (PD) models (Kim et al., 2012; Tamilselvam et al., 2013; Antunes et al., 2014). The activities of other flavonoids, including quercetin (**317**), apigenin (**318**), luteolin (**319**), kaempferol (**320**), diosmetin (**322**), genkwanin (**323**), tricin (**324**) (Wang J. H. et al., 2010; Zhao et al., 2011; Chai et al., 2015), kaempferol-3-O-*β*-rutinoside (**325**), rutin (**326**), kaempferol-3-O-*β*-D-glucopyranoside (**327**), and quercetin-3-O-*β*-D-glucopyranoside (**328**) Tang et al. (2003) have not determined in phytochemical studies of *Valeriana*. The details are shown in Supplementary Table S3 of the supplementary material.

### 4.4 Sesquiterpenoids

Eighty-nine sesquiterpenoids (**357–445**) have been isolated from *Valeriana*. The structures of sesquiterpenoids are shown in Figure 8. Sesquiterpenoids can enhance the activity of nerve growth factor (NGF). For example, compounds madolin A (**357**), madolin B (**358**), volvalerenal A (**359**), B (**360**), F (**363**), G (**364**), kissoone B (**367**), kissoone C (**368**), heishuixiecaoline B (**374**), 1*β*-hydroxyl-8α-acetoxyl-11,11-dimethyl-4-formyl-bicyclogermacren-*E*-4(5),10(14)-diene (**375**), volvalerenic acid D (**378**), isobicyclogermacrenal (**381**), and 4*β*,8a*β*-dimethyl-6*β*-isopropenyl-3,4,4aα, 5,6,7,8,8a-octahydronaphthalen-1(2H)-one (**388**) remarkably promoted the effect on the neurite outgrowth of PC12 cells induced by NGF (Guo et al., 2006; Chen H. W. et al., 2013; Dong et al., 2019). Volvalerenal C (**361**) and heishuixiecaoline A (**370**), C (**372**), and B (**374**) showed protective effects on PC12 cells against Aβ_25–35_ induced toxicity (Glaser et al., 2015). In addition, volvalerenal D (**362**), kissoone B (**367**), and C (**368**), maaliol (**387**), 8-acetoxypatchoulol (**393**), 15-hydroxyspathulenol (**420**), and caryophyllenol A (**435**) prolonged *Drosophila melanogaster* total sleeping time and showed a significant sedative effect (Wu et al., 2014; Dong et al., 2015a). The activities of other sesquiterpenoids had not been determined in the phytochemistry study of *Valeriana*, including isovolvalerenal D (**365**), kissoone A (**366**) (Guo et al., 2006; Wu et al., 2014), and volvalerenic acid A (**369**), C (**371**), B (**373**) (Liu et al., 2012). The details are shown in Supplementary Table S4 of the supplementary material.

### 4.5 Essential oil

Essential oils are the main active constituents of *Valeriana*. Essential oil levels generally range from 0.5% to 3%. In some species, these values are up to 6%–8%. In addition, 0.03% essential oil has been reported in individual species (Wang et al., 2009a). To date, more than 385 essential oil constituents have been analyzed and identified from *Valeriana*, including monoterpenes and sesquiterpenes (Lokar and Moneghini, 1989). Among them, borneol (**446**), bornyl acetate (**447**), and bornyl isovalerate (**448**) are the main constituents of monoterpenes. There are many types of sesquiterpenoids in essential oil of *Valeriana*, such as valeric acid (**449**) and valerenal (**450**) (Chen et al., 2000; Zhou and Huang, 2008a); however, lower sesquiterpenoid levels are noted. Other constituents in the essential oil of *Valeriana* include *α*-pinene (**460**), *β*-pinene (**461**), and phellandrene (**463**) (Taherpour et al., 2010). The essential oil compounds of *Valeriana* are shown in Supplementary Table S5 of the supplementary material.

Essential oil is the main component with sedative activity in *Valeriana* demonstrate significantly prolong sleeping time of mice induced by a hypnotic dose of pentobarbital sodium (Ding, 2012). Essential oil also showed potential antibacterial effects against *Staphylococcus aureus*, *Pseudomonas aeruginosa*, *Bacillus pumilus*, *Candida albicans*, *Escherichia coli*, and *Staphylococcus epidermidis* (Agnihotri et al., 2011). Essential oil also exhibited significant anti-inflammatory and antioxidant effects (Pandian and Nagarajan, 2015; Dyayiya et al., 2016).

### 4.6 Alkaloids

The content of total alkaloids in *Valeriana* is relatively low. Alkaloids are believed as the constituents with antibacterial effect (Babu et al., 2015; Li and Li, 2019). At present, only 13 alkaloids have been isolated from the roots and rhizomes of *Valeriana*, including volvalerine A (Wang P. C. et al., 2016), norphoebine, nantenine, nordelporphine, oxoaporphine, phoebine (Inouye et al., 1974), crystalline (Wang, 2007), valerianine (Franck et al., 1970), actindine (Yin et al., 2006), naphtyridylmethylketone (Thies, 1968a), valerine A, valerine B, and valerine (Wang J. H. et al., 2010). In recent years, there have been relatively few studies on the alkaloids of *Valeriana* due to their low content; thus, these compounds are unlikely to contribute to the therapeutic effect of *Valeriana* (Thies, 1968a).

### 4.7 Others

Except for the constituents mentioned earlier, some other compounds were also isolated, namely, caffeic acid, *p*-coumaric acid, gallic acid (Jugran et al., 2019), isoferulic acid (Wang S. J. et al., 2014), decursitin A, decursitin B, decursidin, 3′(*S*)-acetoxy-4′(*R*)-angeloyloxy-3′,4′-dihydroxanthyletin, dibutyl phthalate, phenanthrene, hydroxybenzoic acid, chlorogenic acid, benzoic acid, oleic acid, (-)-bornyl caffeate, tannin (Wang et al., 2009a, Wang et al., 2014 S. J.; Jugran et al., 2019; Wang S. L. et al., 2020), zansiumloside A (Wan et al., 2016), linoleic acid, palmitic acid, nonadecyl alcohol, *β*-carotene (Wang et al., 2009a, Wang et al., 2014 S. J.; Jugran et al., 2019; Wang S. L. et al., 2020), epoxylathyrol 3,5-dibutyrate (Thies, 1968a and *β*-sitosterol (Wang S. L. et al., 2020).

## 5 Conclusion and perspectives

There are more than 200 species of *Valeriana*, and only the species of *V. officinalis*, *V. jatamansi*, and *V. amurensis* had been systematically studied compared with other species. Most studies on the medicinal parts of *Valeriana* have focused on their roots and rhizomes or whole plants, and few studies have focused on the potential medicinal parts of leaves, seeds and flowers. To effectively promote the utilization of *Valeriana* resources, other common species, such as *V. hardwickii*, *V. alternifolia*, and *V. fauriei* should also be the focus of related studies, and these studies should include their different medicinal parts. The traditional efficacies of roots and rhizomes from *Valeriana* were calming fright and tranquilizing mind, promoting Qi and blood, activating blood circulation and regulating menstruation, dispelling wind and eliminating dampness, regulating Qi-flowing to relieve pain, promoting digestion, and checking diarrhea. The corresponding clinical applications were to treat nervous system diseases of insomnia, anxiety, hysteria, epilepsy, neurasthenia and manic-depressive psychosis, cardiovascular system diseases of palpitation, arrhythmias, coronary heart disease and pulmonary edema, pain and inflammation of rheumatism, rheumatic arthralgia, hepatitis, abdominal distension and pain, gynecological diseases of anemia, menoxenia and dysmenorrhea, dyspepsia of diarrhea, indigestion, and diarrhea and dysentery. As the scientific evidence, the pharmacological effects and effective constituents related to traditional uses of *Valeriana* are summarized in Table 2. At present, except for treating insomnia, other traditional clinical applications of *Valeriana* have not been conducted in modern clinical practice. Therefore, more clinical studies should be performed based on the traditional efficacies or uses of *Valeriana* so that they can be utilized in the treatment of diseases of the nervous system, cardiovascular system, gynecology, dyspepsia, and digestive system. In pharmacological studies, experiments assessing the sedative, hypnotic, antispasmodic, analgesic, antidepressant, anxiolytic, anticonvulsant, antiepileptic, neuroprotective, antibacterial, antiviral, cytotoxic, and antitumor effects as well as cardiovascular and cerebrovascular system improvements, were performed on the extracts or components of *Valeriana* roots and rhizomes. The phytochemistry of some *Valeriana* has been deeply investigated and more than 800 compounds have been isolated or identified, including 259 iridoids, 57 lignans, 40 flavonoids, 89 sesquiterpenoids, 13 alkaloids, and 385 essential oils. *In vitro* activity screening experiments found that lignans exhibited anti-inflammatory and neuroprotective effects, iridoids demonstrated anti-inflammatory and cytotoxic effects, flavonoids exhibited sedative and anti-inflammatory effects, sesquiterpenoids displayed sedative and neuroprotective effects, and essential oils demonstrated sedative effects. However, in fact, most effective fractions and active compounds of *Valeriana* have not been investigated in-depth studies related to drugs development. Only essential oil with the sedative effect of has been developed to treat insomnia clinically. Therefore, future studies should focus on developing effective fractions or active compounds of *Valeriana* into new drugs to treat diseases associated with neurodegeneration, cardiovascular and cerebrovascular, inflammation, and tumors. In addition, a large amount of polysaccharides in the water decoction of *Valeriana* has never been studied. In fact, recent studies on plant polysaccharides showed their extensive pharmacological activities and their medicinal values are worthy of development and utilization.

**TABLE 2:** Pharmacological effects and effective constituents related to traditional uses of *Valeriana*.

Traditional efficacies	Traditional clinical applications	Pharmacological effects	Effective constituents
Calming fright and tranquilizing mind	Nervous system: insomnia; anxiety; hysteria; psychosis epilepsy; neurasthenia; and manic-depressive	Sedative and hypnotic	Iridoids; essential oil; and flavonoids
		Antidepressant and anxiolytic	Iridoids; essential oil; and sesquiterpenoids
		Anticonvulsant and antiepileptic	Iridoids and essential oil
		Neuroprotective	Lignans; essential oil; sesquiterpenoids; and iridoids
Promoting Qi and blood and activating blood circulation regulating menstruation	Cardiovascular system: palpitation; coronary heart disease; pulmonary edema; and arrhythmias	Cardiovascular and cerebrovascular system improvements	Essential oil
	Gynecology: anemia; menoxenia; Dysmenorrhea	Antispasmodic and analgesic	Iridoids and essential oil
Dispelling wind and eliminating dampness and regulating Qi-flowing to relieve pain	Pain and inflammation: rheumatic arthralgia; hepatitis; abdominal distension and pain; traumatic injuries; gingivitis; pericoronitis and dental caries; toothache; soreness and weakness of waist and knees	Antispasmodic and analgesic	Iridoids and essential oil
Promoting digestion and checking diarrhea	Dyspepsia: indigestion; diarrhea and dysentery; and vomiting and diarrhea caused by heatstroke	Antibacterial and antiviral	Alkaloids and essential oil

Compared with other similar reports recently (Jugran, et al., 2019; Dhiman, et al., 2020; Orhan, I. E., 2021), we pointed out for the first time that *Valeriana* as a traditional Chinese medicine, its potential drugs is far from being effectively developed. In view of this problem, we systematically summarized the available medicinal species of *Valeriana*, revealing the relationship between their traditional applications, bioactivities, effective fractions, and active constituents, based on which the future study is clarified for their subsequent development and utilization. Overall, our review will promote the development and utilization of potential drugs in *Valeriana* and avoid wasting their medicinal resources.
