# Supplementary material for: The potential of Valeriana as a traditional Chinese medicine: traditional clinical applications, bioactivities, and phytochemistry
Source: Front Pharmacol. 2022 Sep 21;13:973138. doi: 10.3389/fphar.2022.973138 (PMC9534556; doi:10.3389/fphar.2022.973138)
Supplement: Supplementary file 1 [file Table1.DOCX]

Supplementary Material

**Table S1** Iridoids isolated from the genus *Valeriana* and their activities

| **No.** | **Compound names** | **Activities** | **Sources** | **Parts** | **References** |
| --- | --- | --- | --- | --- | --- |
|  | Jatamanvaltrate A | Cytotoxic | *Valeriana jatamansi* Jones (*V. jatamansi*) | Whole plants | Lin et al. (2009) |
|  | Jatamanvaltrate B | Cytotoxic; Anti-inflammatory | *V. jatamansi*; *Valeriana officinalis* L. (*V. officinalis*) | Whole plants; Roots and rhizomes | (Lin et al., 2009; Liu et al., 2021; Wang et al., 2009a) |
|  | Jatamanvaltrate C | Cytotoxic | *V. jatamansi*; *V. officinalis* | Whole plants | (Lin et al., 2009; Wang et al., 2009a) |
|  | Jatamanvaltrate D | Cytotoxic | *V. jatamansi* | Whole plants | Lin et al. (2009) |
|  | Jatamanvaltrate E | Cytotoxic; Anti-inflammatory | *V. jatamansi* | Whole plants;  Roots and rhizomes | (Lin et al., 2009; Liu et al., 2021) |
|  | Jatamanvaltrate F | Cytotoxic | *V. jatamansi* | Whole plants | Lin et al. (2009) |
|  | Jatamanvaltrate G | Cytotoxic; Neuroprotective | *V. jatamansi* | Whole plants; Roots | (Lin et al., 2009; Xu et al., 2011a) |
|  | Jatamanvaltrate H | Cytotoxic; Neuroprotective | *V. jatamansi* | Whole plants | (Lin et al., 2009; Yu et al., 2010) |
|  | Valeriotriate B | Cytotoxic | *V. jatamansi* | Whole plants; Roots | (Lin et al., 2009; Xu et al., 2011a) |
|  | Valeriotetrate A | Cytotoxic | *V. jatamansi* | Whole plants | (Lin et al., 2009; Yu et al., 2010) |
|  | Didrovaltrate acetoxyhydrin | Cytotoxic | *V. jatamansi* | Whole plants | Lin et al. (2009) |
|  | Valeriotetrate B |  | *Valeriana wallichii* DC. (*V. wallichii*) | Roots | Wang et al. (2008) |
|  | Valerjatadoid B |  | *V. jatamansi* | Roots and rhizomes | Yang et al. (2015) |
|  | Suspensolide F | Neuroprotective | *Valeriana amurensis* P. Smirn. ex Kom. (*V. amurensis*) | Roots and rhizomes | Wan et al. (2016) |
|  | Valeriotetrate C | Cytotoxic; Anti-inflammatory | *V. jatamansi*; *V. officinalis* | Roots and rhizomes | (Liu et al., 2021; Wang et al., 2008; Lin et al., 2010b) |
|  | Chlorovaltrate E | Cytotoxic | *V. jatamansi* | Whole plants | Lin et al. (2013) |
|  | Chlorovaltrate F | Cytotoxic | *V. jatamansi* | Whole plants | Lin et al. (2013) |
|  | Chlorovaltrate G | Cytotoxic | *V. jatamansi* | Whole plants | Lin et al. (2013) |
|  | Chlorovaltrate H | Cytotoxic | *V. jatamansi* | Whole plants | Lin et al. (2013) |
|  | Chlorovaltrate I | Cytotoxic | *V. jatamansi* | Whole plants | Lin et al. (2013) |
|  | Chlorovaltrate J | Cytotoxic | *V. jatamansi* | Whole plants | Lin et al. (2013) |
|  | Chlorovaltrate K | Cytotoxic | *V. jatamansi* | Whole plants | Lin et al. (2013) |
|  | Jatamandoid A | Neuroprotective | *V. jatamansi* | Roots | Xu et al. (2011a) |
|  | Jatadoid B | Neuroprotective | *V. jatamansi* | Roots | Xu et al. (2012c) |
|  | Volvaltrate B | Cytotoxic | *V. officinalis* ; *V. jatamansi* | Roots; Whole plants | (Wang et al., 2009a; Lin et al., 2010b, 2013) |
|  | Jatamanvaltrate L | Cytotoxic | *V. jatamansi* | Whole plants | Lin et al. (2009) |
|  | Jatamanvaltrate M | Cytotoxic | *V. jatamansi* | Whole plants | Lin et al. (2009) |
|  | 5-Hydroxydidrovaltrate | Cytotoxic | *V. jatamansi* | Whole plants | Lin et al. (2009) |
|  | Isovaleroxyhydroxy-dihydrovaltrate / Patriscadoid / IVHD-valtrate | Cytotoxic | *V. jatamansi*; *V. amurensis* | Whole plants;  Roots and rhizomes | (Lin et al. 2009; Janaína et al., 2018) |
|  | ΠHD-acevaltrate |  | *Valeriana polystachya* Sm. (*V. polystachya*)； | Roots and rhizomes | Janaína et al. (2018) |
|  | 11-Homohydroxldihydrovaltrate |  | *V. jatamansi* | Roots and rhizomes | Tang et al. (2002) |
|  | Didrovaltrate | Cytotoxic | *V. officinalis*; *V. jatamansi*;  *V. amurensis* | Whole plants; Rhizomes | (Lin et al. 2009; Thies, 1968b) |
|  | Homodidrovaltrate |  | *V. jatamansi* | Roots and rhizomes | (Liu, 2020; Bos et al., 2002; Thies, 1968a) |
|  | AHD-valtrate |  | *V. jatamansi* | Roots and rhizomes | (Liu, 2020; Bos et al., 2002) |
|  | Isodidrovaltrate |  | *V. jatamansi* | Roots and rhizomes | (Bos et al., 2002; Kucaba et al., 1980) |
|  | Jatamanvaltrate X | Cytotoxic | *V. jatamansi* | Whole plants | Lin et al. (2013) |
|  | Jatamanvaltrate Y | Cytotoxic | *V. jatamansi* | Whole plants | Lin et al. (2013) |
|  | Nardostaehin | Cytotoxic | *V. jatamansi* | Whole plants | Lin et al. (2013) |
|  | Valdiate |  | *V. officinalis* | Roots | Granicher et al. (1995) |
|  | Stenopterin A |  | *Valeriana stenoptera* Diels (*V. stenoptera*) | Whole plants | Dong et al. (2015a) |
|  | Patrinoside-aglucone | Neuroprotective | *V. stenoptera* | Whole plants | Dong et al. (2015a) |
|  | Loganin |  | *V. amurensis* | Roots and rhizomes | Wan et al. (2016) |
|  | 1-*epi*-Bosnarol | Cytotoxic | *Valeriana dioscoridis* Sibth. & Sm. (*V. dioscoridis*) | Roots | Kırmızıbekmeza et al. (2018) |
|  | 8-*epi*-Deoxyloganin aglycone | Cytotoxic | *V. dioscoridis* | Roots | Kırmızıbekmeza et al. (2018) |
|  | Valerosidate/Valerosidatum |  | *V. officinalis*; *V. wallichii* | Roots and rhizomes | (Inouye et al., 1974; Thies, 1970) |
|  | Valerosidatumpentaacetat |  | *V. officinalis*; *V. wallichii* | Roots and rhizomes | Inouye et al. (1974) |
|  | Dioscoridin B | Cytotoxic | *V. dioscoridis* | Roots | Kırmızıbekmeza et al. (2018) |
|  | 10-Acetylpatrinoside | Cytotoxic | *V. dioscoridis* | Roots | (Lin et al., 2009; Kırmızıbekmeza et al., 2018) |
|  | 10,2′-Diacetylpatrinoside | Cytotoxic | *V. dioscoridis* | Roots | Kırmızıbekmeza et al. (2018) |
|  | Volvaltrate C |  | *V. officinalis* | Roots | Amanzadeh et al. (2002) |
|  | Volvaltrate D |  | *V. officinalis* | Roots | Maurya et al. (2020) |
|  | Valerianoside A |  | *V. jatamansi* | Roots | Maurya et al. (2020) |
|  | Jatadomin D | Anti-inflammatory | *V. jatamansi* | Roots | Wang et al. (2020a) |
|  | Stenopterin B | Neuroprotective | *V. stenoptera* | Whole plants | Dong et al. (2015a) |
|  | Dioscoridin C | Cytotoxic | *V. dioscoridis* | Roots | Kırmızıbekmeza et al. (2018) |
|  | Valeriotriate A |  | *V. jatamansi* | Roots | Yu et al. (2010) |
|  | Valerialloside A |  | *V. jatamansi* | Roots | Maurya et al. (2020) |
|  | Patrinoside | Neuroprotective | *Valeriana fauriei* Briq. (*V. fauriei*); *V. amurensis* | Roots and rhizomes | (Nishiya et al., 1994; Wang et al., 2012a) |
|  | Kanokoside D |  | *V. fauriei* | Roots and rhizomes | Nishiya et al. (1994) |
|  | Kanokoside A | Neuroprotective | *V. fauriei*; *V. amurensis* | Roots and rhizomes | (Nishiya et al., 1994; Wang et al., 2012a) |
|  | Kanokoside C |  | *V. fauriei* | Roots and rhizomes | Nishiya et al., 1994 |
|  | Chlorovaltrate Y |  | *V. jatamansi* | Roots and rhizomes | Liu, (2020) |
|  | Valejatadoid B |  | *V. jatamansi* | Roots and rhizomes | Liu et al. (2021) |
|  | Dihydrocornin |  | *Valeriana glechomifolia* F.G.Mey. (*V. glechomifolia*) | Roots and aerial parts | Salles et al. (2000) |
|  | 10-Isovaleryl kanokoside C |  | *V. fauriei* | Roots and rhizomes | Guo et al. (2006) |
|  | Jatamanvaltrate I | Cytotoxic | *V. jatamansi* | Whole plants | Lin et al. (2009) |
|  | Jatamanvaltrate J | Cytotoxic | *V. jatamansi* | Whole plants | Lin et al. (2009) |
|  | Jatamanvaltrate K | Cytotoxic | *V. jatamansi* | Whole plants | Lin et al. (2009) |
|  | 10-Isovaleroxy-valtrathydrin | Anti-inflammatory | *V. jatamansi* | Roots and rhizomes | Liu et al. (2021) |
|  | Isovaltrate acetoxyhydrin |  | *V. jatamansi* | Roots and rhizomes | Liu, (2020) |
|  | Patriscabrin C |  | *V. jatamansi* | Roots and rhizomes | Liu, (2020) |
|  | Valtrate hydrin B_1_ |  | *Valeriana alliariifolia* var. *tiliifolia* (Troickij) V.E.Avet. (*V. alliariifolia*) | Aerial parts | Holzl et al. (1976) |
|  | Valtrate hydrin B_2_ |  | *V. alliariifolia* | Aerial parts | Holzl et al. (1976) |
|  | Valtrate hydrin B_3_ | Cytotoxic | *V. alliariifolia*; *Valeriana sorbifolia* Kunth (*V. sorbifolia*) | Aerial parts | Xu et al. (2007) |
|  | Valtrate hydrin B_4_ |  | *Valeriana alliariifolia* Vahl. | Aerial parts | Koch et al. (1985) |
|  | Valtrate hydrin B_5_ |  | *V. alliariifolia* | Aerial parts | Koch et al. (1985) |
|  | Valtrate hydrin B_6_ |  | *V. alliariifolia* | Aerial parts | Koch et al. (1985) |
|  | Valtrate hydrin B_7_ | Cytotoxic | *V. alliariifolia*; *V. sorbifolia* | Aerial parts | Koch et al. (1985) |
|  | Valtrate hydrin B_8_ |  | *V. alliariifolia* | Aerial parts | Holzl et al. (1976) |
|  | Acetoxydesiovaleroxy-1-*α*-acetoxy-isovaleroxy isovaltratehydrin |  | *Valeriana sisymbriifolia* Vahl. (*V. sisymbriifolia*) | Roots and rhizomes | Amanzadeh et al. (2002) |
|  | 10-Acetoxyvaltrathydrin | Cytotoxic | *V. jatamansi* | Whole plants | Lin et al. (2009) |
|  | Isovaltrate isovaleroyloxyhydrin |  | *V. officinalis*; *V. jatamansi* | Roots | Xu et al. (2012b) |
|  | 10-Acetoxy-1-homovaltrate hydrin |  | *V. jatamansi* | Roots and rhizomes | Tang et al. (2002) |
|  | 10-Acetoxy-1-acevaltratehydrin |  | *V. jatamansi* | Roots and rhizomes | Tang et al. (2002) |
|  | Valeriandiod F | Cytotoxic  Anti-inflammatory | *V. jatamansi* | Roots and rhizomes | (Liu et al., 2021; Xu et al., 2012) |
|  | Sorbifolivaltrate C | Cytotoxic | *V. sorbifolia* | Aerial parts | Xu et al. (2007) |
|  | Sorbifolivaltrate D | Cytotoxic | *V. sorbifolia* | Aerial parts | Xu et al. (2007) |
|  | Jatamanvaltrate P | Cytotoxic | *V. jatamansi* | Roots and rhizomes | (Yang et al., 2015; Maurya et al., 2020; Wang et al., 2014b) |
|  | Jatamanvaltrate Q | Anti-inflammatory | *V. jatamansi* | Roots and rhizomes | (Liu et al., 2021; Yang et al., 2015) |
|  | Valerjatadoid A |  | *V. jatamansi* | Roots and rhizomes | Yang et al. (2015) |
|  | Jatadoid D |  | *V. jatamansi* | Roots and rhizomes | Liu, (2020) |
|  | Valejatadoid F | Anti-inflammatory | *V. jatamansi* | Roots and rhizomes | Liu et al. (2021) |
|  | Jatamanvaltrate Z1 | Cytotoxic | *V. jatamansi* | Whole plants | Lin et al. (2017) |
|  | Jatamanvaltrate Z2 | Cytotoxic | *V. jatamansi* | Whole plants | Lin et al. (2017) |
|  | Jatamanvaltrate Z3 | Cytotoxic | *V. jatamansi* | Whole plants | Lin et al. (2017) |
|  | Valeriandoid A | Neuroprotective | *V. jatamansi* | Roots | Xu et al. (2012b) |
|  | Valeriandoid B |  | *V. jatamansi* | Roots | Xu et al. (2012b) |
|  | Chlorovaltrate | Cytotoxic | *V. jatamansi* | Roots and rhizomes | (Xu et al., 2012b; Wang et al., 2014b) |
|  | Rupesin B | Cytotoxic | *V. jatamansi* | Whole plants; Roots and Rhizomes | (Xu et al., 2012b; Wang et al., 2014b) |
|  | Valechlorine |  | *V. officinalis* | Roots | Popov et al. (1974) |
|  | Chlorovaltrate L | Cytotoxic | *V. jatamansi* | Whole plants | Lin et al. (2013) |
|  | Chlorovaltrate M | Cytotoxic | *V. jatamansi* | Whole plants | Lin et al. (2013) |
|  | Chlorovaltrate N | Cytotoxic | *V. jatamansi* | Whole plants | Lin et al. (2013) |
|  | Chlorovaltrate O | Cytotoxic | *V. jatamansi* | Whole plants | Lin et al. (2013) |
|  | Sorbifolivaltrate A | Cytotoxic | *V. sorbifolia* | Aerial parts | Xu et al. (2007) |
|  | Sorbifolivaltrate B | Cytotoxic | *V. sorbifolia* | Aerial parts | Xu et al. (2007) |
|  | Valtrate | Cytotoxic; Sedative | *V. officinalis*; *V. jatamansi* | Whole plants  Roots and Rhizomes | Lin et al. (2009) |
|  | Isovaltrate | Cytotoxic; Sedative | *V. officinalis*; *V. jatamansi*;  *V. sorbifolia* | Rhizomes; Aerial part | (Thies, 1968b; Xu et al., 2007) |
|  | Acevaltrate / acevaltratum | Cytotoxic; Sedative | *V. officinalis*; *V. jatamansi* | Whole plants;  Roots and Rhizomes | (Lin et al., 2009; Thies, 1968b) |
|  | Diavaltrate |  | *V. glechomifolia* | Roots and rhizomes | Salles et al. (2000) |
|  | 1-*β*-Acevaltrate |  | *V. jatamansi*; *V. alliariifolia* | Roots and rhizomes | Holzl et al. (1984) |
|  | 1-Homoacevaltrate |  | *V. jatamansi* | Roots and rhizomes | (Tang et al. 2002; Bos et al., 2002) |
|  | 1-Homoisoacevaltrate |  | *V. jatamansi* | Roots and rhizomes | Tang et al. (2002) |
|  | 7-*epi*-Deacetyl-isovaltrate |  | *V. officinalis* | Roots | Popov et al. (1974) |
|  | Hydroxylvaltrate |  | *V. officinalis*; *V. jatamansi* | Roots | (Bos et al., 2002; Popov et al., 1974) |
|  | Seneciovaltrate | Cytotoxic | *V. sorbifolia* | Aerial parts | Xu et al. (2007) |
|  | Deacetylisovaltrare |  | *V. officinalis* | Roots | Popov et al. (1974) |
|  | Valjatrate F |  | *V. jatamansi* | Roots  Aerial parts | Su, (2017) |
|  | 1-*α*-Aceisovaltrate |  | *V. sisymbriifolia* | Roots and rhizomes | Amanzadeh et al. (2002) |
|  | Jatamanvaltrate N | Neuroprotective | *V. officinalis* | Roots | Xu et al. (2012a) |
|  | Jatamanvaltrate O |  | *V. officinalis* | Roots | Xu et al. (2012a) |
|  | Jatamanvaltrate V | Cytotoxic | *V. jatamansi* | Whole plants | Lin et al. (2013) |
|  | Jatamanvaltrate W | Cytotoxic; Anti-inflammatory | *V. jatamansi* | Whole plants | (Lin et al., 2013; Liu et al., 2021) |
|  | Patriscadoid I | Anti-inflammatory | *V. jatamansi* | Roots and rhizomes | Liu et al. (2021) |
|  | Patriscadoid II |  | *V. jatamansi* | Roots and rhizomes | Liu et al. (2021) |
|  | Patriscadoid Ⅲ |  | *V. jatamansi* | Roots and rhizomes | Liu, (2020) |
|  | Patriscadoid Ⅳ |  | *V. jatamansi* | Roots and rhizomes | Liu, (2020) |
|  | Valejatadoid D | Anti-inflammatory | *V. jatamansi* | Roots and rhizomes | Liu et al. (2021) |
|  | Valejatadoid E | Anti-inflammatory | *V. jatamansi* | Roots and rhizomes | Liu et al. (2021) |
|  | Jatamanvaltrate Q | Cytotoxic | *V. jatamansi* | Whole plants | Lin et al. (2013) |
|  | Jatamanvaltrate R | Cytotoxic | *V. jatamansi* | Whole plants | (Lin et al., 2013; Dong et al., 2015b) |
|  | Jatamanvaltrate T | Cytotoxic | *V. jatamansi* | Whole plants | Lin et al. (2013) |
|  | Jatairidoid A | Neuroprotective | *V. jatamansi* | Roots | Xu et al. (2012b) |
|  | Jatairidoid B | Neuroprotective | *V. jatamansi* | Roots | Xu et al. (2012b) |
|  | Valejatadoid C / Jatadoid C |  | *V. jatamansi* | Roots and rhizomes | Liu et al. (2021) |
|  | Valeriandoid D |  | *V. jatamansi* | Roots | Xu et al. (2012d) |
|  | Valeriandoid E |  | *V. jatamansi* | Roots | (Wang et al., 2020a; Xu et al., 2012d) |
|  | Jatamanvaltrate S | Cytotoxic | *V. jatamansi* | Whole plants | (Lin et al., 2013; Dong et al., 2015b) |
|  | Jatamanvaltrate U | Cytotoxic | *V. jatamansi* | Whole plants | Lin et al. (2013) |
|  | Jatadoid A | Neuroprotective | *V. jatamansi* | Roots | Xu et al. (2012c) |
|  | Valjatrate E |  | *V. jatamansi* | Roots | Su, (2017) |
|  | Valjatrate G |  | *V. jatamansi* | Roots | Su, (2017) |
|  | Valjatrate H |  | *V. jatamansi* | Roots | Su, (2017) |
|  | Desoxidodidrovaltrate | Cytotoxic | *V. jatamansi* | Roots | (Xu et al., 2011a; Tan et al., 2019) |
|  | 8,11-Desoxididrovaltrate |  | *V. wallichii* | Roots and rhizomes | Bos et al. (2002) |
|  | 8,11-Desoxihomodidrovaltratr |  | *V. wallichii* | Roots and rhizomes | Bos et al. (2002) |
|  | Jatamanin D |  | *V. jatamansi* | Whole plants | Lin et al. (2010) |
|  | Rupesin E | Cytotoxic Antibacterial | *V. jatamansi* | Whole plants | Quan et al. (2019a) |
|  | 4-*β*-Hydroxy-8-*β*-methoxy-10-methylene-2,9-dioxatricyclo [4.3.1.0] pyran |  | *V. jatamansi* | Roots and rhizomes | Nishiya et al., 1994 |
|  | Chlorovaltrate Z |  | *V. jatamansi* | Roots and rhizomes | Liu, (2020) |
|  | Jatamanin C | Cytotoxic | *V. jatamansi* | Whole plants; Roots | Lin et al. (2010) |
|  | Valejatanin B | Cytotoxic | *V. jatamansi* | Roots | Liu et al. (2017) |
|  | 1,5-Dihydroxy-3,8-epoxyvalechlorine | Neuroprotective | *V. jatamansi*; *V. officinalis*;  *V. wallichii* | Roots and rhizomes | (Lin et al., 2010b; Xu et al., 2012b; Quan et al., 2019a) |
|  | Jatamanin O | Cytotoxic | *V. jatamansi* | Roots and rhizomes | (Yang et al., 2015; Lin et al., 2010; Li et al., 2013) |
|  | Jatamanin P | Cytotoxic | *V. jatamansi* | Roots and rhizomes | Li et al. (2013) |
|  | Valeriandoid C | Neuroprotective | *V. jatamansi* | Roots | Xu et al. (2012b) |
|  | Valejatadoid A |  | *V. jatamansi* | Roots and rhizomes | Liu et al. (2021) |
|  | Jatadomin B | Anti-inflammatory | *V. jatamansi* | Roots | Wang et al. (2020a) |
|  | Jatairidoid C | Neuroprotective | *V. jatamansi* | Roots | Xu et al. (2012b) |
|  | Chlorovaltrate P | Cytotoxic | *V. jatamansi* | Roots | Wang et al. (2017) |
|  | Chlorovaltrate Q | Cytotoxic | *V. jatamansi* | Roots | (Quan et al., 2019a; Wang et al., 2017) |
|  | Chlorovaltrate R | Cytotoxic | *V. jatamansi* | Roots | Wang et al. (2017) |
|  | Chlorovaltrate S | Cytotoxic | *V. jatamansi* | Roots | Wang et al. (2017) |
|  | Chlorovaltrate T | Cytotoxic | *V. jatamansi* | Roots | Wang et al. (2017) |
|  | (4*β*,8*β*)-8-Methoxy-3-methoxy-10-methylene-2,9-dioxatricyclo [4.3.1.0] decan-4-ol | Neuroprotective | *V. jatamansi* | Roots | (Quan et al., 2019a; Wang et al., 2017) |
|  | Chlorovaltrate A | Cytotoxic | *V. jatamansi* | Roots | Wang et al. (2017) |
|  | (1*R*,3*R*,5*R*,7*S*,8*R*,9*S*)-3,8-Epoxy-1-O-ethyl-5-hydroxyvalechlorine | Cytotoxic | *V. jatamansi* | Roots | (Quan et al., 2019a; Wang et al., 2017) |
|  | 8-Methoxy-4-acetoxy-3-chlormethyl-10-methylen-2,9-dioxa-tricyclo [4.3.1.0^3,7^]  decan | Cytotoxic | *V. jatamansi* | Roots | Wang et al. (2017) |
|  | (1*S*,3*R*,5*R*,7*S*,8*R*,9*S*)-3,8-Epoxy-1-O-ethyl-5-hydroxyvalechlorine | Neuroprotective | *V. jatamansi* | Roots | (Wang et al., 2020a, 2017) |
|  | (1*R*,3*R*,5*R*,7*S*,8*R*,9*S*)-3,8-Epoxy-1-O-methyl-5-hydroxyvalechlorine | Neuroprotective | *V. jatamansi* | Roots | (Wang et al., 2020a, 2017) |
|  | Chlorovaltrate U |  | *V. jatamansi* | Roots | Tan et al. (2019) |
|  | Chlorovaltrate V |  | *V. jatamansi* | Roots | Tan et al. (2019) |
|  | Chlorovaltrate W |  | *V. jatamansi* | Roots | Tan et al. (2019) |
|  | Jatamanin R | Cytotoxic | *V. jatamansi* | Roots and rhizomes | Quan et al. (2019a) |
|  | Jatamanin S | Cytotoxic | *V. jatamansi* | Roots and rhizomes | Quan et al. (2019a) |
|  | Jatamanin T | Cytotoxic | *V. jatamansi* | Roots and rhizomes | Quan et al. (2019a) |
|  | Jatamanin U | Cytotoxic | *V. jatamansi* | Roots and rhizomes | Quan et al. (2019a) |
|  | Jatadomin C | Anti-inflammatory | *V. jatamansi* | Roots | Wang et al. (2020a) |
|  | (3*S*,4*S*,5*S*,7*S*,8*S*,9*S*)-3,8-Ethoxy-7-dihydroxy-4,8-dimethylperhydrocyclopenta-[c]pyran | Cytotoxic | *V. jatamansi* | Roots | Lin et al. (2010) |
|  | (3*S*,4*S*,4a*S*,6*S*,7*S*,7a*R*)-4,7-Dimethyloctahydro-3,7-epoxycyclopenta[c]pyran-6-yl acetate |  | *V. jatamansi* | Roots | Liu et al. (2017) |
|  | Jatamanin W |  | *V. jatamansi* | Roots and rhizomes | Quan et al. (2019b) |
|  | Jatamanin X |  | *V. jatamansi* | Roots and rhizomes | Quan et al. (2019b) |
|  | (3*S*,4*R*,5*S*,7*S*,8*S*,9*S*)-3,8-Epoxy-7-hydroxy-4,8-dimethylperhydrocyclopenta[c]pyran | Cytotoxic | *V. jatamansi* | Roots | Lin et al. (2010) |
|  | Valejatanin C | Cytotoxic | *V. jatamansi* | Roots | Liu et al. (2017) |
|  | Jatamanin V |  | *V. jatamansi* | Roots and rhizomes | Quan et al. (2019b) |
|  | Jatamanin B |  | *V. jatamansi* | Whole plants; Roots | Lin et al. (2010) |
|  | Jatamanin E |  | *V. jatamansi*; *V. amurensis* | Whole plants; Roots and rhizomes | (Janaína et al., 2018; Lin et al., 2010) |
|  | Jatamanin H |  | *V. jatamansi* | Whole plants; Roots | (Lin et al., 2010; Li et al., 2013) |
|  | Jatamanin I |  | *V. jatamansi* | Whole plants; Roots | (Lin et al., 2010; Li et al., 2013) |
|  | Jatamanin N | Cytotoxic | *V. jatamansi* | Root and Rhizome | (Lin et al., 2010; Li et al., 2013) |
|  | Volvaltrate A |  | *V. officinalis*; *V. jatamansi* | Roots | (Wang et al., 2020a; Lin et al., 2010) |
|  | Stenopterin C |  | *V. stenoptera* | Whole plants | Dong et al. (2015a) |
|  | Stenopterin D |  | *V. stenoptera* | Whole plants | Dong et al. (2015a) |
|  | Valeridoid B |  | *V. jatamansi* | Roots and rhizomes | Quan et al. (2020b) |
|  | Valeridoid C |  | *V. jatamansi* | Roots and rhizomes | Quan et al. (2020b) |
|  | Valeridoid D |  | *V. jatamansi* | Roots and rhizomes | Quan et al. (2020b) |
|  | Polystachyn A |  | *V. polystachya* | Roots and rhizomes | Janaína et al. (2018) |
|  | Jatadomin A | Anti-inflammatory | *V. jatamansi* | Roots | Wang et al. (2020a) |
|  | 4-Hydroxy-8-methoxy-3-methyl-10-methylene-2,9-dioxatricyclo (4,3,1,0^3,7^) -decane |  | *V. jatamansi* | Whole plants | Jugran etval. (2019) |
|  | Baldrinal | Cytotoxic; Sedative | *V. officinalis*; *V. amurensis*; *V. jatamansi* | Roots and Rhizomes | (Thies, 1968b; Xu et al., 2012a; Jugran etval., 2019) |
|  | Decyl baldrinal | Sedative | *V. jatamansi* | Roots | Su, (2017) |
|  | 11-Methoxyviburtinal | Cytotoxic | *V. jatamansi* | Roots | (Xu et al., 2012a; Chen et al., 2005) |
|  | 11-Ethoxyviburtinal |  | *V. officinalis* | Aerial parts | Fan et al. (2020) |
|  | Valejatanin A | Cytotoxic  Antibacterial | *V. officinalis* | Aerial parts | Fan et al. (2020) |
|  | Homobaldrinal |  | *V. officinalis*; *V. jatamansi* | Roots | (Nishiya et al., 1994; Xu et al., 2012a) |
|  | Desacylbaldrinal | Cytotoxic | *V. jatamansi* | Roots | Tan et al. (2019) |
|  | Chlorovaltrate B |  | *V. wallichii* | Whole plants | Lin et al. (2013) |
|  | Chlorovaltrate C |  | *V. wallichii* | Whole plants | Lin et al. (2013) |
|  | Chlorovaltrate D |  | *V. wallichii* | Whole plants | Lin et al. (2013) |
|  | Chlorovaltrate X / Valejatadoid G |  | *V. jatamansi* | Roots | Liu et al. (2021) |
|  | Jatamanin A |  | *V. jatamansi*; *V. amurensis* | Whole plants; Roots | (Janaína et al., 2018; Li et al., 2013) |
|  | Xiecaoside B |  | *V. amurensis* | Roots and rhizomes | Wang et al. (2014a) |
|  | Valejatadoid H |  | *V. jatamansi* | Roots and rhizomes | Wang et al. (2021b) |
|  | (5*S*,7*S*,8*S*,9*S*)-7-Hydroxy-8-isovaleroyloxy-4,11-dihyronepetalactone |  | *V. officinalis* | Roots | Han et al. (2012) |
|  | (5*S*,7*S*,8*S*,9*S*)-7-Hydroxy-10-isovaleroyloxy-4,11-dihyronepetalactone |  | *V. officinalis* | Roots | Han et al. (2012) |
|  | (5*S*,8*S*,9*S*)-10-Isovaleroyloxy-*δ*-4,11-dihyronepetalactone |  | *V. officinalis* | Roots | Han et al. (2012) |
|  | 6-Hydroxy-7-(hydroxylmethyl)-4-methyl-enehexahydrocy-clopenta[c]pyran-1(3H)-one | Neuroprotective | *V. jatamansi*; *V. stenoptera* | Roots and rhizomes | (Wan et al. 2016; Quan et al., 2020b) |
|  | Jatamanin M |  | *V. jatamansi* | Whole plants;  Roots and rhizomes | Quan et al. (2020b) |
|  | Valeridoid A |  | *V. jatamansi* | Roots and rhizomes | Quan et al. (2020b) |
|  | 8,9-Diehydro-7-hydroxy-dolichodial | Anti-inflammatory; Cytotoxic | *V. jatamansi* | Roots and rhizomes | Quan et al. (2020b) |
|  | (5*S*,6*S*,8*S*,9*R*)-6-Isovaleroyloxy-4,11-1,3-diol |  | *V. officinalis* | Roots | Han et al. (2012) |
|  | (5*S*,6*S*,8*S*,9*R*)-1,3-Isovaleroxy-4,11-1,3-diol |  | *V. officinalis* | Roots | Han et al. (2012) |
|  | (5*S*,6*S*,8*S*,9*R*)-3-Isovaleroxy-6-isovaleroyloxy-4,11-1,3-diol | Cytotoxic | *V. officinalis* | Roots | Han et al. (2012) |
|  | Valtral A | Cytotoxic | *V. jatamansi* | Whole plants | Lin et al. (2015) |
|  | Valtral B | Cytotoxic | *V. jatamansi* | Whole plants | Lin et al. (2015) |
|  | Valtral C | Cytotoxic | *V. jatamansi* | Whole plants | Lin et al. (2015) |
|  | Jatamanin J |  | *V. jatamansi* | Whole plants; Roots | Lin et al. (2010) |
|  | Jatamanin L |  | *V. jatamansi* | Whole plants; Roots | Lin et al. (2010) |
|  | Jatamanin Q |  | *V. jatamansi* | Roots | Quan et al. (2019a) |
|  | 4,7-Dimethyloctahydrocyclopenta[c]pyran | Cytotoxic | *V. jatamansi* | Roots | Lin et al. (2010) |
|  | Valeridoid E |  | *V. jatamansi* | Roots and rhizomes | Quan et al. (2020b) |
|  | Valeridoid F | Cytotoxic | *V. jatamansi* | Roots and rhizomes | Quan et al. (2020b) |
|  | Jatamanin G |  | *V. jatamansi* | Whole plants; Roots | (Lin et al., 2010; Li et al., 2013) |
|  | Longiflorone |  | *V. jatamansi* | Whole plants; Roots | (Wang et al., 2009a; Lin et al., 2010) |
|  | Jatamanin F |  | *V. jatamansi* | Whole plants; Roots | (Lin et al., 2010; Li et al., 2013) |
|  | Jatamanin K |  | *V. jatamansi* | Whole plants; Roots | Lin et al. (2010) |
|  | Valeiridoside | Anxiolytic | *Valeriana procera* Kunth | Roots | Alfaro-Romero et al. (2021) |
|  | Patriscabroside Ⅰ |  | *V. amurensis* | Roots and rhizomes | Wang et al. (2014a) |
|  | *α*-Morroniside |  | *V. amurensis* | Roots and rhizomes | Wan et al. (2016) |
|  | *β*-Morroniside |  | *V. amurensis* | Roots and rhizomes | Wan et al. (2016) |
|  | 8-Methylvalepotriate |  | *V. wallichii* | Roots | Wan et al. (2016) |
|  | Patrinovalerosidate | Neuroprotective | *V. amurensis* | Roots and rhizomes | Wan et al. (2016) |
|  | Xiecaoside C |  | *V. amurensis* | Roots and rhizomes | Wang et al. (2014a) |
|  | Xiecaoline A |  | *V. amurensis* | Roots and rhizomes | Wang et al. (2014a) |
|  | Scabroside B |  | *V. amurensis* | Roots and rhizomes | Wang et al. (2014a) |
|  | Jatadomin E | Anti-inflammatory | *V. jatamansi* | Roots | Wang et al. (2020a) |
|  | Isovillosol |  | *V. polystachya* | Roots and rhizomes | Janaína et al. (2018) |
|  | (4*R*,5*R*,7*S*,8*S*,9*S*)-7-Hydroxy-8-hydroxymethyl-4-methyl perhydrocyclopenta |  | *Valeriana laxiflora* DC. (*V. laxiflora*) | Roots and rhizomes | Gu et al. (2004) |
|  | Patriscabrol |  | *V. jatamansi* | Whole plants | Lin et al. (2010) |
|  | Dioscoridin A | Cytotoxic | *V. dioscoridis* | Roots | Kırmızıbekmeza et al. (2018) |
|  | Amurensin A |  | *V. amurensis* | Whole plants | Xie et al. (2019) |
|  | Isopatrinioside | Neuroprotective | *V. jatamansi* | Roots | Tan et al. (2016) |
|  | Vibutinal | Neuroprotective | *V. jatamansi* | Roots | Tan et al. (2016) |
|  | Xiecaoside A |  | *V. amurensis* | Roots and rhizomes | Wang et al. (2014a) |
|  | Valjatrate I |  | *V. jatamansi* | Roots and steams | Su, (2017) |
|  | Villoside aglycone |  | *V. jatamansi* | Whole plants | Jugran etval. (2019) |
|  | 7-Hydroxy-8-(hydroxymethyl)-4methylenehexahydrocyclopenta[c]pyran-1(3H)-one |  | *V. amurensis* | Roots and rhizomes | (Janaína et al. (2018) |
|  | Valtroxal |  | *V. jatamansi* | Roots | Jugran etval. (2019) |
|  | Stenoptenri E |  | *V. stenoptera* | Whole plants | Dong et al. (2015a) |

**Table S2** Lignans isolated from the genus *Valeriana* and their activities

| **No.** | **Compound names** | **Activities** | **Resources** | **Parts** | **References** |
| --- | --- | --- | --- | --- | --- |
|  | 8′-Hydroxypinoresinol | Antitubercular | *V. officinalis*;  *V. jatamansi*;  *V. laxiflora* | Roots and rhizomes | (Lin et al., 2015; Schumacher et al., 2002; Quan et al., 2020a) |
|  | Pinoresinol-4-O-*β*-D-glucopyranoside |  | *V. officinalis*  *V. amurensis* | Roots and rhizomes | (Schumacher et al., 2002; Wang et al., 2012b) |
|  | Pinoresinol-8-O-*β*-D-glucopyranoside | Neuroprotective | *V. amurensis* | Roots and rhizomes | (Wang et al., 2012a, 2012b) |
|  | 8'-Hydroxypinoresinol-4'-O-*β*-D-glucopyranoside |  | *V. officinalis* | Roots | Schumacher et al. (2002) |
|  | 8-Hydroxypinoresinol-4'-O-*β*-D-glucoside | Neuroprotective | *V. officinalis*  *V. amurensis*;  *Valeriana prionophylla* Standl. (*V.* *prionophylla*) | Roots and rhizomes | (Schumacher et al., 2002; Lin et al., 2010b) |
|  | Pinoresinol-4,4'-di-O-*β*-D-glucoside | Neuroprotective | *V. officinalis*  *V. amurensis* | Roots | (Wang et al., 2012a; Schumacher et al., 2002) |
|  | 8-Hydroxypinoresinol | Neuroprotective  Antioxidant  Vasorelaxant | *V. amurensis*  *V.* *prionophylla* | Roots and rhizomes | (Wang et al., 2012a; Piccinelli et al., 2004) |
|  | Pinoresinol | Neuroprotective | *V. jatamansi*  *V. officinalis* | Roots;  Aerial parts | (Lin et al., 2010, Li et al., 2011) |
|  | Prinsepiol | Neuroprotective  Antioxidant | *V. officinalis*;  *V. jatamansi*;  *V. prionophylla* | Roots and rhizomes;  Aerial parts | (Piccinelli et al., 2004; Li et al., 2011; Zuo et al., 2017b)) |
|  | (+)-1-Acetoxypinoresinol |  | *V. officinalis* | Aerial parts | Fan et al. (2020) |
|  | Prinsepiol-4-O-*β*-D-glucopyranoside | Neuroprotective | *V. amurensis*  *V. prionophylla* | Roots and rhizomes | (Janaína et al., 2018; Piccinelli et al., 2004) |
|  | Pinoresinol monomethyl ether | Neuroprotective | *V. jatamansi* | Roots | Li et al. (2011) |
|  | 8-Hydroxypinoresinol-4-O-*β*-D-glucopyranoside | Neuroprotective  Antiarrhythmic | *V. amurensis*  *V. officinalis* | Roots and rhizomes | (Wang et al., 2012a, 2012b; Liu et al., 2021) |
|  | 8'-Hydroxypinoresinol-4-O-*β*-D-glucopyranoside |  | *V. officinalis* | Roots and rhizomes | Zuo et al. (2017b) |
|  | 8-Hydroxypinoresinol-4,4'-di-O-*β*-D-glucopyranoside | Neuroprotective | *V. amurensis* | Roots and rhizomes | Wang et al. (2012a) |
|  | 3'-Demethyl-pinoresinol-4,4'-O-*β*-D-di-glucopyranoside |  | *V. officinalis* | Roots and rhizomes | Zuo et al. (2017b) |
|  | 8,8'-di-Hydroxyl-pinoresinol-4-O-*β*-D-glucopyranoside |  | *V. officinalis* | Roots and rhizomes | Zuo et al. (2017b) |
|  | 8,8'-di-Hydroxyl-pinoresinol-4,4'-di-O-*β*-D-glucopyranoside | Neuroprotective | *V. amurensis* | Roots and rhizomes | Janaína et al. (2018) |
|  | Fraxireslnol-4'-O-*β*-D-glucopyranoside |  | *V. amurensis*  *V. prionophylla* | Roots and rhizomes | (Wang et al., 2012b; Piccinelli et al., 2004) |
|  | Syringaresinol-4,4'-di-O-*β*-D-glucopyranoside | Neuroprotective | *V. amurensis* | Roots and rhizomes | Janaína et al. (2018) |
|  | Syringaresinol |  | *V. jatamansi* | Roots and rhizomes | Quan et al. (2020a) |
|  | (+)-Medioresinol |  | *V. jatamansi* | Whole plants | Lin et al. (2010) |
|  | (+)-Medioresinol-4,4'-di-O-*β*-D-glucopyranoside | Neuroprotective | *V. jatamansi* | Roots and rhizomes | (Janaína et al. (2018) |
|  | (+)-Monomethylpinoresinol |  | *V. jatamansi* | Whole plants | Lin et al. (2010) |
|  | (+)-1,5-di-Hydroxy-2(*S*),6(*S*)-di(4-hydroxy-3-methoxyphenyl)-3,7-dioxabicyclo [3.3.0] octane |  | *V. jatamansi* | Roots and rhizomes | Quan et al. (2020a) |
|  | Dipsalignan E |  | *V. jatamansi* | Roots and rhizomes | Quan et al. (2020a) |
|  | 5'-Methoxyl-pinoresinol-4,4'-O-*β*-D-di-glucopyranoside |  | *V. officinalis* | Roots and rhizomes | Zuo et al. (2017b) |
|  | 5'-Hydroxylpinoresinol |  | *V. jatamansi* | Whole plants | Lin et al. (2010) |
|  | 8,9'-di-Hydroxyl-prinsepiol-4-O-*β*-D-glucopyranoside |  | *V. amurensis* | Roots and rhizomes | Wang et al. (2012b) |
|  | (+)-Demethoxypinoresinol |  | *V. jatamansi* | Roots and rhizomes | Quan et al. (2020a) |
|  | (+)-2-(3,4-di-Methoxyphenyl)-6-(3,4-dihydroxyphenyl)-2,7-dioxabicyclo [3,3,0] octane |  | *V. jatamansi* | Roots | Li et al. (2011) |
|  | 4,4'-di-Demethylconiferolyl-3'-demethyl-8,8'-dihydroxyl-pinoresinol |  | *V. officinalis* | Roots and rhizomes | Zuo et al. (2017b) |
|  | Massoniresinol-4'-O-*β*-D-glucoside |  | *V. officinalis* | Roots | Schumacher et al. (2002) |
|  | Berchemol |  | *V. jatamansi* | Whole plants | Lin et al. (2010) |
|  | Berchemol-4'-O-*β*-D-glucoside |  | *V. officinalis* | Roots | Schumacher et al. (2002) |
|  | Lariciresinol |  | *V. jatamansi* | Whole plants | Lin et al. (2010) |
|  | Olivil-4'-O-*β*-D-glucopyranoside | Neuroprotective | *V. amurensis* | Roots and rhizomes | Wang et al. (2012b) |
|  | Lariciresinol-4,4'-di-O-*β*-D-glucopyranoside | Neuroprotective | *V. amurensis* | Roots and rhizomes | Janaína et al. (2018) |
|  | Olivil-4-O-*β*-D-glucopyranoside | Neuroprotective | *V. amurensis* | Roots and rhizomes | Wang et al. (2012b) |
|  | 8-Hydroxylariciresinol-4'-O-*β*-D-glucopyranoside | Neuroprotective | *V. amurensis* | Roots and rhizomes | Wang et al. (2012b) |
|  | Lariciresinol-4-O-*β*-D-glucopyranoside | Neuroprotective | *V. amurensis* | Roots and rhizomes | Wang et al. (2012b) |
|  | Neoarctin A | Neuroprotective | *V. amurensis* | Roots and rhizomes | Wang et al. (2014a) |
|  | Lariciresinol-4'-O-*β*-D-glucopyranoside | Neuroprotective | *V. amurensis* | Roots and rhizomes | Wang et al. (2014a) |
|  | Massoniresinol-3a-O-*β*-D-glucopyranoside | Neuroprotective | *V. amurensis* | Roots and rhizomes | (Wang et al., 2014a; Xue et al., 2016) |
|  | Massoniresinol |  | *V. jatamansi* | Roots | Li et al. (2011) |
|  | 4,4′,9,7′-Tetrahydroxy-3,3′-dimethoxy-7, 9′-epoxylignan |  | *V. jatamansi* | Whole plants | Lin et al. (2010) |
|  | (7*α*H,8′*β*H)-3,3′,8*β*,9-Tetrahydroxy-4,4′-dimethoxy-7,9′-epoxylignan |  | *V. officinalis* | Aerial parts | Fan et al. (2020) |
|  | 4'-O-*β*-D-Glucosyl-9-O-(6''-deoxysaccha-rosyl) olivil |  | *V. officinalis* | Roots | Schumacher et al. (2002) |
|  | Ginkgool |  | *V. officinalis* | Aerial parts | Fan et al. (2020) |
|  | Lariciresinol C |  | *V. officinalis* | Aerial parts | Fan et al. (2020) |
|  | (7*S*,8*R*)-Dehydroconiferyl alcohol-8, 5′-dehydroconiferyl aldehyde-4-O-*β*-D- glucopy ranoside |  | *V. jatamansi* | Roots | Wang et al. (2021a) |
|  | (7*R*,8*S*,8′*R*,9*R*)-Tetrahydro-7-(4-hydroxy-3-methoxyphenyl)-8′-[(4′-hydroxy-3′-methoxyphenyl)methyl]-8-oxirane-8,8′-furandiol |  | *V. officinalis* | Aerial parts | Fan et al. (2020) |
|  | 4'-Demethylpodophyllotoxin | Cytotoxic | *V. wallichii* | Rhizomes | Glaser et al. (2015) |
|  | Podophyllotoxin | Cytotoxic | *V. wallichii* | Rhizomes | Glaser et al. (2015) |
|  | (−)-Matairesinol | Anti-inflammatory | *V. amurensis* | Whole plants | Xie et al. (2019) |
|  | (+)-Cycloolivil |  | *V. jatamansi*  *V. officinalis* | Roots and rhizomes | (Fan et al., 2020; Quan et al.; 2020a) |
|  | (+)-9′-Isovaleroxy-lariciresin | Cytotoxic  Anti-inflammatory | *V. jatamansi* | Roots | Quan et al. (2020a) |

**Table 3** Flavonoids isolated from the genus *Valeriana* and their activities

| **No.** | **Compound names** | **Activities** | **Resources** | **Parts** | **References** |
| --- | --- | --- | --- | --- | --- |
|  | Quercetin |  | *V. officinalis*;  *V. amurensis*;  *Valeriana hardwickii* Wall. (*V. hardwickii*) | Whole plants | (Zhao et al., 2011; Cai et al., 2015; Wang et al., 2010a) |
|  | Apigenin |  | *V. officinalis*  *V. amurensis*  *V. hardwickii* | Whole plants | (Glaser et al., 2015; Cai et al., 2015; Wang et al., 2010a) |
|  | Luteolin |  | *V. officinalis*  *V. amurensis* | Roots and rhizomes | (Glaser et al., 2015; Wang et al., 2010a) |
|  | Kaempferol |  | *V. officinalis*  *V. amurensis* | Roots and rhizomes | (Glaser et al., 2015; Wang et al., 2010a) |
|  | Acacetin | Neuroprotective | *V. officinalis*  *V. amurensis*  *V. hardwickii* | Whole plants | (Glaser et al., 2015; Cai et al., 2015; Wang et al., 2010a) |
|  | Diosmetin |  | *V. officinalis*  *V. amurensis* | Roots and rhizomes | (Glaser et al., 2015; Wang et al., 2010a) |
|  | Genkwanin |  | *V. hardwickii* | Whole plants | Cai et al. (2015) |
|  | Tricin |  | *V. hardwickii* | Whole plants | Cai et al. (2015) |
|  | Kaempferol-3-O-*β*-rutinoside |  | *V. jatamansi* | Roots and rhizomes | Tang et al. (2003) |
|  | Rutin |  | *V. jatamansi* | Roots and rhizomes | Tang et al. (2003) |
|  | Kaempferol-3-O-*β*-D-glucopyranoside |  | *V. jatamansi* | Roots and rhizomes | Tang et al. (2003) |
|  | Quercetin-3-O-*β*-D-glucopyranoside |  | *V. jatamansi* | Roots and rhizomes | Tang et al. (2003) |
|  | 5-Hydroxy-7,4'-dimethoxyflavone |  | *V. hardwickii* | Whole plants | Cai et al. (2015) |
|  | Apigenin-7-O-*α*-L-rhamnopyranosyl (1→6)-*β*-D-glucopyranoside |  | *V. officinalis* | Roots and rhizomes | Zuo et al. (2017a) |
|  | Acacetin-7-O-*α*-L-rhamnopyranosyl (1→6)-*β*-D-glucopyranoside |  | *V. officinalis* | Roots and rhizomes | Zuo et al. (2017a) |
|  | 5-Methoxyl-acacetin-7-O-*α*-L-rhamnopyranosyl (1→6)-*β*-D-glucopyranoside |  | *V. officinalis* | Roots and rhizomes | Zuo et al. (2017a) |
|  | 4'-Methyl-5-methoxyl-flavone-7-O-*α*-L-rhamnopyranosyl (1→6)-*β*-D-glucopyranoside |  | *V. officinalis* | Roots and rhizomes | Zuo et al. (2017a) |
|  | Diosmetin-7-O-*α*-L-rhamnopyranosyl (1→6)-*β*-D-glucopyranoside |  | *V. officinalis* | Roots and rhizomes | Zuo et al. (2017a) |
|  | 6-Methyl-apigenin-7-O-*α*-L-rhamnopyranosyl (1→6)- [*α*-L-rhamnopyranosyl (1→2)]-*β*-D-glucopyranoside |  | *V. officinalis* | Roots and rhizomes | Zuo et al. (2017a) |
|  | Acacetin-7-O-*α*-L-rhamnopyranosyl (1→6)- [*α*-L-rhamnopyranosyl (1→2)] -*β*-D-glucopyranoside |  | *V. officinalis* | Roots and rhizomes | Zuo et al. (2017a) |
|  | 5-Methoxyl-acacetin-7-O-*α*-L-rhamnopyranosyl (1→6)- [*α*-L-rhamnopyranosyl (1→2)] -*β*-D-glucopyranoside |  | *V. officinalis* | Roots and rhizomes | Zuo et al. (2017a) |
|  | 8-Methyl-apigenin-7-O-*β*-D-glucopyranosyl (1→2)-*β*-D-galactopyranoside |  | *V. officinalis* | Roots and rhizomes | Zuo et al. (2017a) |
|  | 6-Methylapigenin | Anxiolytic Anticonvulsant  Sedative | *V. officinalis*;  *V. wallichii*;  *V. hardwickii* | Roots and rhizomes | (Wang et al., 2010a; Marder et al., 2003; Fernández et al., 2004) |
|  | Acacetin-7-O-*β*-D-glucopyranoside |  | *V. jatamansi* | Roots and rhizomes | Tang et al. (2003) |
|  | Apigenin-7-O-*β*-D-glucopyranoside |  | *V. jatamansi* | Roots and rhizomes | Tang et al. (2003) |
|  | (-)-Farrerol |  | *V. hardwickii* | Whole plants | Cai et al. (2015) |
|  | Syzalterin |  | *V. hardwickii* | Whole plants | Cai et al. (2015) |
|  | 5,7-Trihydroxy-3,6,4'-trimethoxyflavanone |  | *V. officinalis* | Aerial parts  Roots | Wang et al. (2010a) |
|  | 8-Hydroxyl-didymin |  | *V. officinalis* | Roots and rhizomes | Zuo et al. (2017a) |
|  | Acacetin-7-O-*β*-sophoroside |  | *V. jatamansi* | Roots and rhizomes | (Wang et al., 2010a; Tang et al., 2003) |
|  | Acacetin-7-O-(6''-O-*α*-L-rhamnopyranosyl)-*β*-sophoroside |  | *V. jatamansi* | Roots and rhizomes | (Wang et al., 2010a; Tang et al., 2003) |
|  | Linarin / acacetin-7-O-rutinoside | Sedative and sleep-enhancing  Anticonvulsant | *V. officinalis*  *V. wallichii* | Roots and rhizomes | (Fernández et al., 2004; Thies, 1968) |
|  | Linarin-2-O-methylbutyrate |  | *V. wallichii* | Rhizomes | Glaser et al. (2015) |
|  | Hesperidin | Neuroprotective | *V. hardwickii* | Whole plants | Cai et al. (2015) |
|  | 2*S* (-)-Hesperidin | Sedative and sleep-enhancing  Anticonvulsant | *V. officinalis*  *V. wallichii* | Roots and rhizomes | (Marder et al., 2003; Fernández et al., 2004; Thies, 1968) |
|  | Leachianone A | Anti-inflammatory | *V. jatamansi* | Roots and rhizomes | Wang et al. (2021b) |
|  | Isosakuranetin |  | *V. hardwickii* | Whole plants | Cai et al. (2015) |
|  | Hesperetin-7-O-*β*-rutinoside |  | *V. wallichii* | Roots and rhizomes | Thies, (1968) |
|  | Catechin |  | *V. jatamansi* | Roots and rhizomes | Jugran etval. (2019) |
|  | Linarin-isovalerianate |  | *V. wallichii* | Roots and rhizomes | Thies, (1968) |

**Table 4** Sesquiterpenoids isolated from the genus *Valeriana* and activities

| **No.** | **Compound names** | **Activities** | **Resources** | **Parts** | **References** |
| --- | --- | --- | --- | --- | --- |
|  | Madolin A | Neuroprotective | *V. officinalis* | Roots | Wang et al. (2010c) |
|  | Madolin B | Neuroprotective | *V. officinalis* | Roots | Chen et al. (2013a) |
|  | Volvalerenal A | Neuroprotective | *V. officinalis* | Roots | (Wang et al., 2010; Chen et al., 2013a) |
|  | Volvalerenal B | Neuroprotective | *V. officinalis* | Roots | (Wang et al., 2010; Chen et al., 2013a) |
|  | Volvalerenal C | Neuroprotective | *V. amurensis*;  *V. officinalis* | Roots | (Wang et al., 2012a, 2010) |
|  | Volvalerenal D | Sedative | *V. officinalis* | Roots | Wang et al. (2010c) |
|  | Volvalerenal F | Neuroprotective | *V. officinalis* | Roots | Chen et al. (2013a) |
|  | Volvalerenal G | Neuroprotective | *V. officinalis* | Roots | Chen et al. (2013a) |
|  | Isovolvalerenal D |  | *V. amurensis* | Roots and rhizomes | Wu et al. (2014) |
|  | Kissoone A |  | *V. fauriei*  *V. amurensis* | Roots and rhizomes | (Wu et al., 2014; Guo et al., 2006) |
|  | Kissoone B | Sedative  Neuroprotective | *V. officinalis*  *V. fauriei*  *V. amurensis* | Roots and rhizomes | (Wang et al., 2010; Wu et al., 2014; Guo et al., 2006) |
|  | Kissoone C | Sedative  Neuroprotective | *V. officinalis*;.  *V. fauriei*;  *V. amurensis* | Roots and rhizomes | (Wang et al., 2010; Wu et al., 2014; Guo et al., 2006) |
|  | Volvalerenic acid A |  | *V. officinalis* | Roots | Wang et al. (2010c) |
|  | Heishuixiecaoline A | Neuroprotective | *V. amurensis* | Roots | Wang et al. (2012a) |
|  | Volvalerenic acid C |  | *V. officinalis* | Roots | Wang et al. (2010c) |
|  | Heishuixiecaoline C | Neuroprotective | *V. amurensis*; | Roots | Wang et al. (2012a) |
|  | Volvalerenic acid B |  | *V. officinalis* | Roots | Wang et al. (2010c) |
|  | Heishuixiecaoline B | Neuroprotective | *V. amurensis* | Roots | (Wang et al., 2012a; Chen et al., 2013a) |
|  | 1*β*-Hydroxyl-8*α*-acetoxyl-11,11-dimethyl-4-formyl-bicyclogermacren-*E*-4(5),10(14)-diene |  | *V. fauriei* | Roots | Liu et al. (2012) |
|  | Bicyclo[8,1,0]5*β*-hydroxyl-7*β*-1acetoxyl-5*α*,11,11′-trimethyl-*E*-1(10)-ene-4*α*,15-olide |  | *V. fauriei* | Roots | Liu et al. (2012) |
|  | Volvalerenal E |  | *V. officinalis* | Roots | Wang et al. (2010c) |
|  | Volvalerenic acid D | Neuroprotective | *V. officinalis* | Roots | Chen et al. (2013a) |
|  | 1*β*,10*α*-Dihydroxyl-8*α*-acetoxyl-10*β*,11,11-trimethyl-4-formyl-bicyclogermacren-*E*-4(5)-ene |  | *V. fauriei* | Roots | Liu et al. (2012) |
|  | 11*α*H-gemacra-1(10)*E*,4*Z*-diene-3-one-12,6*α*-olide |  | *V. fauriei* | Roots | Liu et al. (2012) |
|  | Isobicyclogermacrenal | Sedative  Neuroprotective | *V. officinalis* | Roots | Wang et al. (2010c) |
|  | 13-Hydroxypatchoulol A |  | *V. stenoptera* | Roots and rhizomes | Dong et al. (2015a) |
|  | 11-*epi*-13-Hydroxypatchoulol A |  | *V. stenoptera* | Roots and rhizomes | Dong et al. (2015a) |
|  | Isointermedeol |  | *V. amurensis* | Roots and rhizomes | Dong et al. (2019) |
|  | Valeriananoid A |  | *V. jatamansi* | Roots and rhizomes | Lin et al. (2015) |
|  | Cyperusol |  | *V. stenoptera* | Whole plants | Dong et al. (2015a) |
|  | Maaliol | Sedative  Neuroprotective | *V. amurensis* | Roots and rhizomes | Dong et al. (2019) |
|  | 4*β*,8a*β*-Dimethyl-6*β*-isopropenyl-3,4,4a*α*, 5,6,7,8,8*a*-octahydronaphthalen-1(2*H*)-one |  | *V. amurensis* | Roots and rhizomes | Dong et al. (2019) |
|  | 8-Hydroxy-patchouli alcohol |  | *V. jatamansi* | Roots | Liu et al. (2017) |
|  | 8-Acetoxypatchouli alcohol | Cytotoxic | *V. jatamansi* | Roots | Liu et al. (2017) |
|  | (3*R*)-3-Hydroxypatchoulol |  | *V. jatamansi* | Roots | Liu et al. (2017) |
|  | Patchouli alcohol |  | *V. stenoptera* | Whole plants | Dong et al. (2015a) |
|  | 8-Acetoxypatchoulol | Sedative  Neuroprotective | *V. stenoptera* | Whole plants | Dong et al. (2015a) |
|  | 9-Hydroxypatchoulol |  | *V. stenoptera* | Whole plants | Dong et al. (2015a) |
|  | 9-acetoxypatchoulol |  | *V. stenoptera* | Whole plants | Dong et al. (2015a) |
|  | Valeriananoid B |  | *V. jatamansi* | Roots and rhizomes | Lin et al. (2015) |
|  | Valeriananoid C | Anticholinesterase | *V. jatamansi* | Roots and rhizomes | Lin et al. (2015) |
|  | Valeriananoid D |  | *V. jatamansi* | Roots | Dong et al. (2015b) |
|  | Valeriananoid E |  | *V. jatamansi* | Roots | Dong et al. (2015b) |
|  | Valeriananoid F |  | *V. jatamansi* | Roots | Tan et al. (2016) |
|  | Eudesm-11-en-5*a*-ol |  | *V. amurensis* | Roots and rhizomes | Dong et al. (2019) |
|  | 2-Naphthalenemethanol |  | *V. amurensis* | Whole plants | Xie et al. (2019) |
|  | Eremophila-1(10)-en-4*α*-ol |  | *V. stenoptera* | Roots and rhizomes | Dong et al. (2015a) |
|  | Valeranone |  | *V. stenoptera* | Whole plants | Dong et al. (2015a) |
|  | Valerol A | Cytotoxic | *V. jatamansi* | Roots | (Liu et al., 2017; Dong et al., 2019) |
|  | *α*-Kessyl isovalerate |  | *V. amurensis* | Roots and rhizomes | Dong et al. (2019) |
|  | Valeracetate |  | *V. amurensis* | Roots and rhizomes | Jugran etval. (2019) |
|  | kessyl 3-acetate |  | *V. officinalis* | Roots | Wang et al. (2009a) |
|  | Hydroxyvalerenic acid |  | *V. jatamansi* | Roots and rhizomes | Jugran etval. (2019) |
|  | Acetoxyvalerenic acid |  | *V. jatamansi* | Roots and rhizomes | Jugran etval. (2019) |
|  | Valerenic acid |  | *V. jatamansi* | Roots and rhizomes | Jugran etval. (2019) |
|  | *E*-(-)-3,4-Epoxyvalerenal |  | *V. officinalis* | Roots | Wang et al. (2009a) |
|  | *E*-(-)-3,4-Epoxyvalerenyl acetate |  | *V. officinalis* | Roots | Wang et al. (2009a) |
|  | Mononorvalerenone |  | *V. officinalis* | Roots | Wang et al. (2009a) |
|  | 1-Naphthalenemethanol |  | *V. amurensis* | Whole plants | Xie et al. (2019) |
|  | Clovane-2*β*-isovaleroxy-9*α*-ol |  | *V. jatamansi* | Roots | Dong et al. (2015a) |
|  | 2*α*-acetoxy-1*α*,9*α*-oxidobisbol |  | *V. amurensis* | Roots and rhizomes | Dong et al. (2019) |
|  | Jatamansone |  | *V. jatamansi* | Roots and rhizomes | Jugran etval. (2019) |
|  | volvalerenone A |  | *V. officinalis* | Roots | Wang et al. (2010d) |
|  | 15-hydroxyspathulenol | Sedative | *V. amurensis* | Roots and rhizomes | Wu et al. (2014) |
|  | 4*α*,10*α*-epoxyaromadendrane |  | *V. officinalis* | Roots | Wang et al. (2009a) |
|  | 3*β*-Hydroxyl-*β*-(*cis*)-epoxide-*α*-guaiene |  | *V. stenoptera* | Roots and rhizomes | Dong et al. (2015a) |
|  | 1-Hydroxy-l,11,11-trimethyldecahydrocyclopropane azulene-10-one |  | *V. amurensis* | Roots and rhizomes | Wu et al. (2014) |
|  | Valerianin C |  | *V. fauriei* | Roots | Liu et al. (2012) |
|  | Globulol |  | *V. amurensis* | Roots and rhizomes | Wang et al. (2011) |
|  | Orientalol C |  | *V. officinalis* | Roots | Wang et al. (2011) |
|  | Anismol A |  | *V. officinalis* | Roots | Wang et al. (2011) |
|  | Valerilactones A | Neuroprotective | *V. jatamansi* | Roots | Jugran etval. (2019) |
|  | Valerilactones B | Neuroprotective | *V. jatamansi* | Roots | Jugran etval. (2019) |
|  | Bakkenollides B |  | *V. jatamansi* | Roots | Jugran etval. (2019) |
|  | Bakkenollides H | Neuroprotective | *V. jatamansi* | Roots | Jugran etval. (2019) |
|  | 11-Hydroxypogostol |  | *V. amurensis* | Roots and rhizomes | Dong et al. (2019) |
|  | Pogostol |  | *V. stenoptera* | Whole plants | Dong et al. (2015a) |
|  | Spatulenol |  | *V. officinalis* | Roots | Wang et al. (2011) |
|  | Caryophyllenol A | Sedative | *V. amurensis* | Roots and rhizomes | Wu et al. (2014) |
|  | Bisabola-7(14),10-dien-4*β*,5*β*,15-triol |  | *V. stenoptera* | Roots and rhizomes | Dong et al. (2015a) |
|  | (1*R*,2*R*,7*R*)-2-Acetoxyl-*β*-bisabolol |  | *V. fauriei* | Roots and rhizomes | Nishiya et al., 1994 |
|  | (1*R*,2*R*,7*R*)-2-Hydroxyl-*β*-bisabolol |  | *V. fauriei* | Roots and rhizomes | Nishiya et al., 1994 |
|  | *β*-Bisabolol |  | *V. amurensis* | Roots and rhizomes | Dong et al. (2019) |
|  | Epoxysesquithujene |  | *V. hardwickii* | Roots and rhizomes | Mathela et al. (2007) |
|  | Sesquithujenol |  | *V. hardwickii* | Roots and rhizomes | Mathela et al. (2007) |
|  | Sesquithujene |  | *V. hardwickii* | Roots and rhizomes | Mathela et al. (2007) |
|  | 1*S*,3*S*,4*S*,7*S*3,4-di-Hydroxy-bisabolol |  | *V. amurensis* | Whole plants | Xie et al. (2019) |
|  | 2-Ethylhexyl-4-hydroxybenzoate |  | *V. fauriei* | Roots | Liu et al. (2012) |
|  | Citroside A |  | *V. amurensis* | Roots and rhizomes | Wan et al. (2016) |

**Table S5.** Essential oil analyzed and identified from the genus *Valeriana*

| **No.** | **Compound names** | **Resources** | **Parts** | **References** |
| --- | --- | --- | --- | --- |
|  | Borneol | *V. officinalis*  *V. officinalis*  *V. amurensis*  *V. jatamansi*  *V. wallichii*  *V. hardwickii* | Roots and rhizomes | (Mathela et al., 2007; Zhou and Huang, 2008; Lunz and Stappen, 2021; Bos et al., 1997) |
|  | Bornyl acetate | *V. officinalis*  *V. officinalis*  *V. amurensis*  *V. wallichii*  *V. fauriei*  *V. hardwickii* | Roots and rhizomes | (Mathela et al., 2007; Zhou and Huang, 2008; Sati et al., 2005; Raal et al., 2008; Chung et al., 2012) |
|  | Bornyl isovalerate | *V. officinalis*  *V. amurensis*  *V. wallichii*  *V. fauriei* | Roots and rhizomes | (Mathela et al., 2007; Zhou and Huang, 2008; Bos et al., 1997; Chung et al., 2012) |
|  | Valeric acid | *V. officinalis*  *V. jatamansi*  *V. sisymbriifolia* | Roots and rhizomes | (Vishwakarma et al., 2016; Pirbalouti et al., 2015) |
|  | Z-*β*-Farnesene | *V. jatamansi* | Rhizomes | Mathela et al. (2009) |
|  | Valerenal | *V. officinalis* | Roots and rhizomes | (Lunz and Stappen, 2021; Raal et al., 2007) |
|  | Sesquiterpene alcohol C | *V. officinalis* | Roots and rhizomes | Lunz and Stappen, (2021) |
|  | allo-Aromadendrene | *V. officinalis*  *V. sisymbriifolia* | Roots and rhizomes  Aerial parts | (Lunz and Stappen, 2021; Raal et al., 2007; Javidnia et al., 2010) |
|  | Longiborneol acetate | *V. officinalis* | Roots and rhizomes | (Lunz and Stappen, 2021; Pavlović et al., 2004) |
|  | Valerianol | *V. officinalis* | Roots and rhizomes | (Raal et al., 2007; Pavlović et al., 2004; Maurya et al., 2021) |
|  | Hexanal | *V. wallichii* | Aerial parts  Roots and rhizomes | Sati et al. (2005) |
|  | *α*-Longipinene | *V. wallichii* | Aerial parts  Roots | (Sati et al., 2005; Mathela et al., 2005) |
|  | *β*-Longipinene | *V. jatamansi* | Roots | Verma et al. (2012) |
|  | Valerenolic acid | *V. officinalis* | Roots and rhizomes | Chen et al. (2000) |
|  | *α*-Pinene | *V. officinalis*  *V. amurensis*  *V. jatamansi*  *V. wallichii*  *V. hardwickii*  *V. sisymbriifolia*  *V. alliariifolia* | Roots and rhizomes  Aerial parts | (Sati et al., 2005; Taherpour et al., 2010; Lunz and Stappen, 2021; Pirbalouti et al., 2015; Ding et al., 2011) |
|  | *β*-Pinene | *V. officinalis*  *V. amurensis*  *V. jatamansi*  *V. wallichii*  *V. alliariifolia* | Roots and rhizomes | (Lunz and Stappen, 2021; Taherpour et al., 2010) |
|  | Phellandrene | *V. officinalis*  *V. alliariifolia* | Roots and rhizomes | (Taherpour et al., 2010; Bos et al., 2000) |
|  | Limonene | *V. officinalis*  *V. amurensis*  *V. wallichii*;  *V. sisymbriifolia*; *V. alliariifolia* | Aerial parts  Roots and rhizomes | (Lunz and Stappen, 2021; Sati et al., 2005; Taherpour et al., 2010; Bos et al., 2000) |
|  | *α*-Terpineol | *V. officinalis*  *V. amurensis*  *V. jatamansi*  *V. wallichii*  *V. sisymbriifolia*  *V. alliariifolia* | Aerial parts  Roots and rhizomes | (Bos et al., 1997; Javidnia et al., 2010; Taherpour et al., 2010) |
|  | *γ*-Terpinene | *V. officinalis*  *V. wallichii*  *V. alliariifolia* | Aerial parts  Roots and rhizomes | (Sati et al., 2005; Taherpour et al., 2010; Bos et al., 2000) |
|  | 7-Tetracyclo [6.2.1.0(3.8)0(3.9)] undecanol, 4,4,11,11-tetramethyl | *V. jatamansi* | Rhizomes | Pandian and Nagarajan, (2015) |
|  | Patchoulane | *V. jatamansi* | Rhizomes | Fokialakis et al. (2002) |
|  | Terpinolene | *V. officinalis*  *V. wallichii*  *V. alliariifolia*  *Valeriana italica* Lam. | Aerial parts  Roots and rhizomes | (Sati et al., 2005; Taherpour et al., 2010; Bos et al., 2000; Sundaresan et al., 2012) |
|  | *β*-Caryophyllene | *V. wallichii*  *V. sisymbriifolia*  *V. alliariifolia* | Aerial parts  Roots | (Sati et al., 2005; Javidnia et al., 2010; Taherpour et al., 2010) |
|  | *γ*-Selinene | *V. alliariifolia* | Aerial parts | Taherpour et al. (2010) |
|  | *α*-Santalene | *V. jatamansi*  *V. wallichii* | Aerial parts  Roots and rhizomes | (Sati et al., 2005; Thusoo et al., 2014; Irshad et al., 2012) |
|  | 𝛽-Vatirenene | *V. jatamansi* | Roots |  |
|  | Widdrene | *V. jatamansi* | Rhizomes | Pandian and Nagarajan, (2015) |
|  | 1,3,8-p-Methatrine | *V. jatamansi* | Whole plants | Pavlović et al. (2007) |
|  | Kessane | *V. officinalis*  *V. jatamansi*  *V. wallichii* | Aerial parts  Roots and rhizomes | (Lunz and Stappen, 2021; Raina and Negi, 2015; Lokar and Moneghini, 1989) |
|  | Elemol | *V. officinalis* | Aerial parts | (Raina and Negi, 2015; Singh et al., 2013) |
|  | Bicyclo [7.2.0] undec-4-ene, 4,11,11-trimethyl-8-methylene | *V. jatamansi* | Rhizomes | Pandian and Nagarajan, (2015) |
|  | 2-Pentylfuran | *V. officinalis* | Aerial parts | Raina and Negi, (2015) |
|  | n-Amylisovalerate | *V. jatamansi* | Whole plants | Pavlović et al. (2007) |
|  | Cyperene | *V. jatamansi* | Rhizomes | Pandian and Nagarajan, (2015) |
|  | Azulene | *V. jatamansi* | Roots and rhizomes | Alfaro-Romero et al. (2016) |
|  | trans-Sesquisabinenehydrate | *V. jatamansi* | Rhizomes | Mathela et al. (2009) |
|  | Anethole | *V. officinalis* | Roots | Dyayiya et al. (2016) |
|  | Hesperitinic acid | *V. officinalis* | Roots and rhizomes | Bos et al. (2000) |
|  | 2,5-Bornanediol | *V. officinalis* | Roots | Dyayiya et al. (2016) |
|  | Hinesol | *V. officinalis* | Roots and rhizomes  Aerial parts | (Bos et al. 2000; Raina and Negi, 2015) |
|  | Eudesma-3,7(11)-diene | *V. jatamansi* | Rhizomes | Pandian and Nagarajan, (2015) |
|  | 2-Hydroxycupelene | *V. jatamansi* | Whole plants | Pavlović et al. (2007) |
|  | Camphene | *V. officinalis*  *V. amurensis*  *V. wallichii*  *Valeriana capensis* Thunb. (*V. capensis*)  *Valeriana tuberosa* L. (*V. tuberosa*) | Roots and rhizomes | (Raal et al., 2007; Maurya et al., 2021; Sundaresan et al., 2012; Verma et al., 2011) |
|  | *α*-Copaen-11-ol | *V. jatamansi* | Rhizomes | Pandian and Nagarajan, (2015) |
|  | cis-Linalool oxide | *V. jatamansi* | Rhizomes | Pandian and Nagarajan, (2015) |
|  | Caryophyllene oxide | *V. jatamansi*  *V. tuberosa* | Roots | (Sundaresan et al., 2012; Verma et al., 2011) |
|  | Myrcene | *V. officinalis*  *V. jatamansi*  *V. wallichii*  *V. italica* | Aerial parts  Roots | (Sundaresan et al., 2012; Singh et al., 2013; Jugran et al., 2019) |
|  | Phytol | *V. tuberosa* | Aerial parts | Sundaresan et al. (2012) |
|  | Benzaldehyde | *V. officinalis* | Aerial parts | Raina and Negi, (2015) |
|  | *γ*-Curcumene | *V. jatamansi* | Rhizomes | Fokialakis et al. (2002) |
|  | 15-Acetoxyvaleranone | *V. officinalis*  *V. italica* | Roots and rhizomes | (Lunz and Stappen, 2021; Sundaresan et al., 2012) |
|  | Verticiol | *V. jatamansi* | Whole plants | Pavlović et al. (2007) |
|  | Neoclovene oxide | *V. jatamansi* | Rhizomes | Pandian and Nagarajan, (2015) |
|  | Hexadecanoic acid | *V. officinalis*  *V. tuberosa*  *V. italica* | Roots  Aerial parts | (Raal et al., 2008; Sundaresan et al., 2012) |
|  | *α*-Curcumene | *V. officinalis*;  *V. wallichii* | Roots | (Bos et al., 1997; Raal et al., 2008) |
|  | 1,1,4a-Trimethyl-5,6-dimethylenedecahydronaphthalene | *V. jatamansi* | Rhizomes | Pandian and Nagarajan, (2015) |
|  | Epiglobulol | *V. jatamansi* | Rhizomes | Pandian and Nagarajan, (2015) |
|  | Nonacosane | *V. jatamansi* | Rhizomes | Pandian and Nagarajan, (2015) |
|  | Camphor | *V. officinalis*  *V. amurensis*  *V. wallichii* | Roots and rhizomes | Lunz and Stappen, (2021)  Pavlović et al. (2004) |
|  | trans-*β*-Farnesene | *V. officinalis* | Roots and rhizomes | Raina and Negi, (2015) |
|  | Caryophyllene | *V. jatamansi* | Rhizomes | Pandian and Nagarajan, (2015) |
|  | Palustrol | *V. jatamansi* | Whole plants | Pavlović et al. (2007) |
|  | Valencene | *V. officinalis*  *V. capensis*  *V. eallichii* | Roots and rhizomes | (Lunz and Stappen, 2021; Mathela et al., 2005) |
|  | Guaiacol | *V. jatamansi* | Roots and rhizomes | Rawat et al. (2017) |
|  | Naphthalene | *V. tuberosa* | Aerial parts | Sundaresan et al. (2012) |
|  | Guaiol | *V. jatamansi*  *V. officinalis* | Roots and rhizomes | (Rawat et al., 2017; Chen et al., 2015) |
|  | Valeric acid, 4-pentadecyl ester | *V. jatamansi* | Rhizomes | Pandian and Nagarajan, (2015) |
|  | Myrtenol | *V. officinalis*  *V. officinalis* | Roots and rhizomes | (Bos etb al., 2000; Raina and Negi, 2015) |
|  | Aromadendrene oxide-(2) | *V. jatamansi* | Rhizomes | Pandian and Nagarajan, (2015) |
|  | cis-Sesquisabinene hydrate | *V. jatamansi* | Rhizomes | Mathela et al. (2009) |
|  | Sabinene | *V. officinalis*  *V. officinalis*  *V. amurensis*  *V. wallichii* | Roots and rhizomes | (Bos et al. 1997, 2000; Pavlović et al., 2004; Du et al., 2006) |
|  | Tetracosane | *V. jatamansi* | Rhizomes | Pandian and Nagarajan, (2015) |
|  | Ledol | *V. officinalis*  *V. officinalis* | Roots and rhizomes | (Raal et al., 2008; Du et al., 2006) |
|  | 2-(5-hydroxypent-2-ynyl)-3-oxocyclopentyl] thioacetic acid, s-t-butyl ester | *V. jatamansi* | Rhizomes | Pandian and Nagarajan, (2015) |
|  | Citronellol | *V. officinalis* | Roots and rhizomes | Pavlović et al. (2004) |
|  | *α*-Farnesene | *V. officinalis*  *V. officinalis*  *V. amurensis* | Roots and rhizomes | (Raal et al., 2008; Yu et al., 20011) |
|  | Viridiflorene | *V. officinalis* | Aerial parts | Raina and Negi, (2015) |
|  | Terpin-4-ol | *V. italica*  *V. tuberosa* | Roots  Aerial parts | Sundaresan et al. (2012) |
|  | Bornyl hexanoate | *V. wallichii* | Roots and rhizomes | Bos et al. (1997) |
|  | Phenethylisovalerate | *V. jatamansi* | Rhizomes | Fokialakis et al. (2002) |
|  | cis-2,6-Dimethyl-2,6-octadiene | *V. jatamansi* | Rhizomes | Fokialakis et al. (2002) |
|  | Isovaleric acid | *V. officinalis*  *V. officinalis*  *V. jatamansi*  *V. sisymbriifolia* | Roots and rhizomes  Aerial parts | (Raal et al., 2008; Pirbalouti et al., 2015; Maurya et al., 2021; Irshad et al., 2012) |
|  | Juniper camphor | *V. jatamansi* | Roots and rhizomes | Alfaro-Romero et al. (2016) |
|  | *α*-Himachalene | *V. jatamansi* | Whole plants | Pavlović et al. (2007) |
|  | Eremophilene | *V. jatamansi* | Rhizomes | Pandian and Nagarajan, (2015) |
|  | *α*-Curcumene | *V. jatamansi* | Rhizomes | Fokialakis et al. (2002) |
|  | epi-*β*-Santalene | *V. jatamansi* | Rhizomes | Mathela et al. (2009) |
|  | Propyl valerate | *V. jatamansi* | Rhizomes | Pandian and Nagarajan, (2015) |
|  | *α*-Muurolene | *V. jatamansi*  *V. italica* | Roots | (Sundaresan et al., 2012; Irshad et al, 2012) |
|  | Dehydro-aromadendrene | *V. jatamansi*  *V. wallichii* | Roots and rhizomes  Aerial parts | (Sati et al., 2005; Mathela et al., 2005; Irshad et al., 2012) |
|  | *α*-Elemol | *V. jatamansi* | Whole plants | Pavlović et al. (2007) |
|  | *β*-patchoulane | *V. jatamansi* | Rhizomes | Pandian and Nagarajan, (2015) |
|  | Butylatedhydroxy toluene | *V. jatamansi* | Rhizomes | Mathela et al. (2009) |
|  | *α*-Fenchene | *V. officinalis*  *V. italica* | Roots and rhizomes | (Pavlović et al., 2007; Maurya et al., 2021; Bos et al., 2000; Sundaresan et al., 2012) |
|  | 3-Iodomethyl-3,6,6-trimethyl-cyclohexene | *V. jatamansi* | Rhizomes | Pandian and Nagarajan, (2015) |
|  | Myrtle acetate | *V. officinalis*  *V. amurensis* | Aerial parts  Roots | (Raal et al., 2007; Raina and Negi, 2015) |
|  | *β*-Eurjunene | *V. jatamansi* | Whole plants | Pavlović et al. (2007) |
|  | Selinadiene alcohol | *V. officinalis* | Roots | Raal et al. (2008) |
|  | cis-Farnesol | *V. jatamansi* | Roots and rhizomes | Alfaro-Romero et al. (2016) |
|  | 1-Isopropenyl-3,3-dimethyl-5-(3-methyl-1-oxo-2-butenyl) cyclopentane | *V. jatamansi* | Rhizomes | Pandian and Nagarajan, (2015) |
|  | Isoquinolin-6,7-diol-1-carboxylic acid | *V. jatamansi* | Rhizomes | Pandian and Nagarajan, (2015) |
|  | Cycloheptane | *V. jatamansi* | Rhizomes | Pandian and Nagarajan, (2015) |
|  | trans-Pinocarvyl acetate | *V. officinalis* | Roots and rhizomes | (Raal et al., 2008; Pavlović et al., 2007) |
|  | *β*-Bisabolene | *V. sisymbriifolia*  *V. officinalis* | Aerial parts | (Javidnia et al., 2010; Singh et al., 2013) |
|  | *β*-Atlantone | *V. sisymbriifolia* | Aerial parts | Pirbalouti et al. (2015) |
|  | Eudesma-4(14),11-diene | *V. jatamansi* | Rhizomes | Pandian and Nagarajan, (2015) |
|  | cis-Adamantane-2-carboxylic acid | *V. jatamansi* | Roots and rhizomes | Pandian and Nagarajan, (2015) |
|  | Glyceryllinolenate | *V. jatamansi* | Rhizomes | Pandian and Nagarajan, (2015) |
|  | 14-Hydroxy-9-epi-(E)-caryophyllene | *V. sisymbriifolia* | Aerial parts | Pirbalouti et al. (2015) |
|  | *γ*-Eudesmol | *V. officinalis* | Aerial parts | Raina and Negi, (2015) |
|  | Methyl *β*-methylvalerate | *V. officinalis* | Roots | Dyayiya et al. (2016) |
|  | Isoestragol | *V. officinalis* | Roots | Dyayiya et al. (2016) |
|  | Bornyl butyrate | *V. jatamansi* | Rhizomes | Fokialakis et al. (2002) |
|  | *α*-Humulene | *V. officinalis*  *V. jatamansi*  *V. sisymbriifolia*  *V. tuberosa*  *V. italica* | Whole plants  Aerial parts  Roots | (Javidnia et al., 2010; Sundaresan et al., 2012; Agnihotri et al., 2011; Bhatt et al., 2012) |
|  | 2,6-Diamino-4-cyclohexyl-4H-thiopyran-3,5-dicarbonitrile | *V. jatamansi* | Rhizomes | Pandian and Nagarajan, (2015) |
|  | Methyl 9,12,15-octadecatrienoate | *V. jatamansi* | Rhizomes | Pandian and Nagarajan, (2015) |
|  | Nonanal | *V. italica* | Aerial parts | Sundaresan et al. (2012) |
|  | *α*-Eudesmol | *V. jatamansi* | Rhizomes | Fokialakis et al. (2002) |
|  | (*Z*)-*β*-Ocimene | *V. officinalis* | Aerial parts | Raina and Negi, (2015) |
|  | Pacifigoriadiene isomer A | *V. officinalis* | Roots | Raal et al. (2008) |
|  | (*E*)-*β*-Damascenone | *V. officinalis*;  *V. montana*;  *Valeriana braunii-blanquetii* (*V. braunii-blanquetii*);  *V. tuberosa* | Aerial parts | (Sundaresan et al., 2012; Raina and Negi, 2015) |
|  | Dodecanoic acid | *V. officinalis*  *V. braunii-blanquetii* | Aerial parts | Raina and Negi, (2015) |
|  | *β*-Humulene | *V. officinalis* | Roots and rhizomes | Wang et al. (2010) |
|  | Carotol | *V. jatamansi* | Whole plants | Agnihotri et al. (2011) |
|  | Ledane | *V. jatamansi* | Rhizomes | Pandian and Nagarajan, (2015) |
|  | Hexanoate | *V. wallichii* | Roots and rhizomes | Bos et al. (1997) |
|  | *γ*-Muurolene | *V. italica*  *V. jatamansi* | Roots and rhizomes | (Verma et al., 2012; Sundaresan et al., 2012) |
|  | Germacrene B | *V. jatamansi*  *V. officinalis* | Whole plants | (Pavlović et al., 2007; Agnihotri et al., 2011) |
|  | Palmitic acid | *V. jatamansi* | Rhizomes | Pandian and Nagarajan, (2015) |
|  | Germacrene D | *V. officinalis*  *V. italica* | Roots | (Raal et al., 2008; Sundaresan et al., 2012) |
|  | *δ*-3-Carene | *V. officinalis* | Aerial parts | Raina and Negi, (2015) |
|  | Zingiberene | *V. officinalis*  *V. tuberosa* | Roots  Aerial parts | (Raal et al., 2008; Sundaresan et al., 2012) |
|  | Pentadecane | *V. italica* | Aerial parts | Sundaresan et al. (2012) |
|  | Terpinen-4-ol | *V. officinalis* | Roots and rhizomes | Pavlović et al. (2007) |
|  | Megastigma-4,6(e),8(z)-triene | *V. jatamansi* | Rhizomes | Pandian and Nagarajan, (2015) |
|  | cis-*β*-Faresene | *V. jatamansi* | Whole plants | Agnihotri et al. (2011) |
|  | *δ*-Elemene | *V. officinalis*  *V. jatamansi*  *V. officinalis*  *V. wallichii* | Roots and rhizomes | Bos et al. (1997) |
|  | *γ*-Elemene | *V. wallichii* | Roots and rhizomes | Bos et al. (1997) |
|  | 3-Methylvaleric acid | *V. officinalis*  *V. officinalis*  *V. jatamansi*  *V. wallichii*  *V. sisymbriifolia* | Aerial parts  Roots and rhizomes | (Sati et al., 2005; Pirbalouti et al., 2015; Pandian and Nagarajan, 2015; Thusoo et al., 2014) |
|  | Methyl 10,13-octadecadiynoate | *V. jatamansi* | Rhizomes | Pandian and Nagarajan, (2015) |
|  | 4-Hydroxy | *V. jatamansi* | Roots and rhizomes | Pandian and Nagarajan, (2015) |
|  | Germacrene-D-4-ol | *V. italica* | Roots | Sundaresan et al. (2012) |
|  | *β*-Methasone valerate | *V. jatamansi* | Roots and rhizomes | Pandian and Nagarajan, (2015) |
|  | Methyl palmitate | *V. jatamansi* | Rhizomes | Fokialakis et al. (2002) |
|  | 10-epi-*γ*-Eudesmol | *V. officinalis* | Aerial parts | Raina and Negi, (2015) |
|  | Selin-11-en-4α-ol | *V. braunii-blanquetii* | Aerial parts | Raina and Negi, (2015) |
|  | Benzyl isovalerate | *V. jatamansi* | Roots and rhizomes | Pandian and Nagarajan, (2015) |
|  | 3-p-Menthene | *V. wallichii* | Aerial parts  Roots | Sati et al. (2005) |
|  | cis-Nerolidol | *V. wallichii* | Roots and rhizomes | Bos et al. (1997) |
|  | Methyl thymol | *V. wallichii*  *V. officinalis* | Aerial parts  Roots and rhizomes | (Sati et al., 2005; Bos et al., 2000) |
|  | Methyl carvacrol | *V. wallichii*  *V. officinalis* | Aerial parts  Roots and rhizomes | (Sati et al., 2005; Bos et al., 2000) |
|  | Eicosane | *V. jatamansi* | Rhizomes | Pandian and Nagarajan, (2015) |
|  | trans-α-Bergamotene | *V. officinalis* | Roots and rhizomes | Pavlović et al. (2007) |
|  | 14-Oxononadec-10-enoic acid, methyl ester | *V. jatamansi* | Rhizomes | Pandian and Nagarajan, (2015) |
|  | 6,7-Dimethoxy-2-tetralone | *V. jatamansi* | Rhizomes | Fokialakis et al. (2002) |
|  | Carvotanacetone | *V. wallichii* | Aerial parts  Roots and rhizomes | (Bos et al., 1997; Sati et al., 2005) |
|  | Thymyl acetate | *V. wallichii* | Roots and rhizomes | Bos et al. (1997) |
|  | Thymyl isovalerate | *V. wallichii* | Roots and rhizomes | Bos et al. (1997) |
|  | Nerolidol | *V. jatamansi* | Roots and rhizomes | (Pandian and Nagarajan, 2015; Chen et al., 2015) |
|  | Methyl thymol ether | *V. wallichii*  *V. jatamansi*  *V. italica*  *V. hardwickii* | Roots and rhizomes | (Mathela et al., 2007; Bos et al., 1997; Sundaresan et al., 2012) |
|  | Khusilic acid | *V. jatamansi* | Rhizomes | Pandian and Nagarajan, (2015) |
|  | *α*-Muurolol | *V. officinalis*  *V. braunii-blanquetii* | Aerial parts | Raina and Negi, (2015) |
|  | 2-Phenylethyl hexanoate | *V. wallichii* | Roots and rhizomes | Bos et al. (1997) |
|  | Copaene | *V. jatamansi* | Roots | Lopes et al. (2005) |
|  | *β*-Patchoulene | *V. officinalis*  *V. jatamansi*  *V. wallichii* | Roots and rhizomes | (Bos et al., 1997; Lokar et al., 1989) |
|  | Methyl eugenol | *V. tuberosa* | Aerial parts | Sundaresan et al. (2012) |
|  | Estra-1,3,5(10)-trien-17*β*-ol | *V. jatamansi* | Rhizomes | Pandian and Nagarajan, (2015) |
|  | Deoxysericealactone | *V. jatamansi* | Rhizomes | Fokialakis et al. (2002) |
|  | *α*-Tocopherol | *V. jatamansi* | Rhizomes | Pandian and Nagarajan, (2015) |
|  | *β*-Elemene | *V. jatamansi*  *V. officinalis*  *V. officinalis*  *V. wallichii* | Aerial parts  Roots and rhizomes | (Sati et al., 2005; Mathela et al., 2005; Wang et al., 2010) |
|  | *α*-Elemene | *V. officinalis* | Roots and rhizomes | Wang et al. (2010) |
|  | Ethyl 9,9-diformylnona-2,4,6,8-tetraenoate | *V. jatamansi* | Rhizomes | Fokialakis et al. (2002) |
|  | Thymol | *V. officinalis*  *V. italica* | Roots and rhizomes | (Bos et al., 2000; Sundaresan et al., 2012) |
|  | *γ*-Sitosterol | *V. jatamansi* | Rhizomes | Pandian and Nagarajan, (2015) |
|  | Cedren-13-ol, 8- | *V. jatamansi* | Rhizomes | Pandian and Nagarajan, (2015) |
|  | Selina-4,11-diene | *V. officinalis* | Roots and rhizomes | Wang et al. (2010) |
|  | Selinene isomer | *V. wallichii* | Roots and rhizomes | Bos et al. (1997) |
|  | *β*-Helmiscapene | *V. officinalis* | Roots and rhizomes | Wang et al. (2010) |
|  | 1,2-Dissoproylbenzene | *V. officinalis* | Roots and rhizomes | Wang et al. (2010) |
|  | Benzyl benzoate | *V. tuberosa*  *V. italica* | Aerial parts | Sundaresan et al. (2012) |
|  | Isovalerate | *V. wallichii* | Roots and rhizomes | Bos et al. (1997) |
|  | Decanal | *V. tuberosa* | Aerial parts | Sundaresan et al. (2012) |
|  | Chiapin B | *V. jatamansi* | Rhizomes | Pandian and Nagarajan, (2015) |
|  | *α*-Gurjunene | *V. officinalis*  *V. officinalis*  *V. amurensis*  *V. jatamansi*  *V. wallichii* | Roots  Aerial parts | (Lunz and Stappen, 2021; Javidnia et al., 2010) |
|  | *β*-Gurjunene | *V. officinalis*  *V. wallichii*  *V. sisymbriifolia* | Aerial parts  Roots and rhizomes | (Lunz and Stappen, 2021; Sati et al., 2005; Javidnia et al., 2010; Thusoo et al., 2014) |
|  | Murolan-3,9(11)-diene-10-peroxy | *V. jatamansi* | Rhizomes | Pandian and Nagarajan, (2015) |
|  | *γ*-Gurjunene | *V. officinalis*  *Valeriana edulis* Nutt. (*V. edulis*  ) | Roots | Dyayiya et al. (2016) |
|  | Artemiseole | *V. officinalis*;  *V. edulis*;  *V. capensis* | Roots | (Dyayiya et al., 2016; Rawat et al., 2017) |
|  | Cyclohexadecane | *V. jatamansi* | Rhizomes | Pandian and Nagarajan, (2015) |
|  | Citronellyl acetate | *V. officinalis* | Roots and rhizomes | Pavlović et al. (2007) |
|  | *γ*-Terpinene-7-al | *V. officinalis* | Aerial parts | Raina and Negi, (2015) |
|  | Tumerol | *V. wallichii* | Roots and rhizomes | Bos et al. (1997) |
|  | Methyl isovalerate | *V. officinalis*  *V. edulis* | Roots | Dyayiya et al. (2016) |
|  | Estragole | *V. officinalis*  *V. edulis* | Roots | Dyayiya et al. (2016) |
|  | Methyl (10E)-10-heptadecen-8-ynoate | *V. jatamansi* | Rhizomes | Pandian and Nagarajan, (2015) |
|  | Isospathulenol | *V. italica* | Roots | Sundaresan et al. (2012) |
|  | Octadecane | *V. tuberosa* | Aerial parts | Sundaresan et al. (2012) |
|  | Ethyl isovalerate | *V. officinalis*  *V. edulis* | Roots | Dyayiya et al. (2016) |
|  | *α*-Cubenene | *V. italica* | Roots | Sundaresan et al. (2012) |
|  | 2,6-Dimethyl-8-(tetrahydropyran-2-yloxy)-octa-2,6-dien-1-ol | *V. jatamansi* | Rhizomes | Pandian and Nagarajan, (2015) |
|  | Isopentyl isovalerate | *V. edulis*  *V. jatamansi* | Roots and rhizomes | (Dyayiya et al., 2016; Jugran et al., 2019) |
|  | epi-*α*-Cadinol | *V. officinalis*  *V. wallichii* | Roots  Aerial parts | (Lunz and Stappen, 2021; Sati et al., 2005) |
|  | 1,4-Dimethoxy benzene | *V. italica* | Aerial parts | Sundaresan et al. (2012) |
|  | trans-Caryophyllene | *V. officinalis*  *V. officinalis*  *V. jatamansi*  *V. alliariifolia*  *V. italica*  *V. tuberosa*  *V. hardwickii* | Roots and rhizomes  Aerial parts | (Mathela et al., 2007; Taherpou et al., 2010; Sundaresan et al., 2012; Chen et al., 2015) |
|  | 9-Methyl-Z, Z-10,12-hexadecadien-1-ol acetate | *V. jatamansi* | Rhizomes | Pandian and Nagarajan, (2015) |
|  | Squalene | *V. jatamansi* | Rhizomes | Pandian and Nagarajan, (2015) |
|  | Calarene | *V. anurensis*  *V. jatamansi*  *V. alliariifolia* | Aerial parts  Roots | (Taherpou et al., 2010; Lokar et al., 1989; Liu e al., 2013) |
|  | *α*-Guaiene | *V. officinalis*  *V. officinalis*  *V. jatamansi*  *V. wallichii* | Roots and rhizomes | (Raal et al., 2008; Thusoo et al., 2014; Irshad et al., 2012; Lokar et al., 1989; Dyayiya et al., 2016) |
|  | trans-*β*-Guaiene | *V. wallichii* | Aerial parts  Roots | Sati et al. (2005) |
|  | Bulnesol | *V. wallichii*  *V. officinalis* | Aerial parts  Roots and rhizomes | (Sati et al., 2005; Mathela et al., 2005; Raina and Negi, 2015) |
|  | (Z)-*γ*-Bisabolene | *V. wallichii* | Aerial parts  Roots | Sati et al. (2005) |
|  | 2-Phenylethyl isovaltrate | *V. jatamansi* | Roots and rhizomes | Bos et al. (1997) |
|  | Desmethoxy encecalin | *V. wallichii* | Aerial parts  Roots and rhizomes | (Sati et al., 2005; Mathela et al., 2005) |
|  | Dihydro-eudesmol | *V. wallichii* | Aerial parts  Roots and rhizomes | (Sati et al., 2005; Mathela et al., 2005) |
|  | Laurenene | *V. wallichii* | Aerial parts  Roots and rhizomes | (Sati et al., 2005; Mathela et al., 2005) |
|  | Xanthorrhizol | *V. jatamansi*  *V. wallichii* | Roots and rhizomes | Bos et al. (1997) |
|  | Xanthorrhizol isomer | *V. wallichii* | Roots and rhizomes | Bos et al. (1997) |
|  | Spathulenol | *V. officinalis*  *V. amurensis*  *V. jatamansi*  *V. sisymbriifolia*  *V. alliariifolia* | Roots and rhizomes | (Raal et al., 2008; Maurya et al., 2021; Samaneh et al., 2010; Lokar et al., 1989) |
|  | Clocortolone pivalate | *V. jatamansi* | Rhizomes | Pandian and Nagarajan, (2015) |
|  | trans-Isoliminene | *V. officinalis*  *V. sisymbriifolia*  *V. alliariifolia* | Roots and rhizomes | Samaneh et al. (2010) |
|  | Vulgarone B | *V. capensis*  *V. sisymbriifolia*  *V. alliariifolia* | Roots and rhizomes | (Samaneh et al., 2010; Rawat et al., 2017) |
|  | Globulol | *V. italica*  *V. officinalis* | Roots | (Raal et al., 2008; Sundaresan et al., 2012) |
|  | Patchoulol | *V. officinalis*  *V. jatamansi* | Roots | (Irshad et al., 2012; Wang et al., 2010) |
|  | Kessanyl acetate | *V. officinalis* | Roots and rhizomes | Bos et al. (2000) |
|  | Kessyl acetate | *V. officinalis*  *V. wallichii* | Roots and rhizomes | (Bos et al., 1997; Du et al., 2006) |
|  | Kessyl glycol | *V. officinalis* | Roots and rhizomes | Chen et al. (2000) |
|  | *α*-Kessyl alcohol | *V. officinalis*  *V. officinalis* | Roots and rhizomes | Wang et al. (2010) |
|  | Guaiane epoxide | *V. jatamansi* | Roots | Verma et al. (2011) |
|  | Tricyclene | *V. officinalis* | Roots and rhizomes | Bos et al. (2000) |
|  | C_15_H_26_ | *V. jatamansi* | Roots and rhizomes | Bos et al. (1997) |
|  | Linalool | *V. officinalis*  *V. amurensis*  *V. wallichii*  *V. italica*  *Valeriana minutiflora* Hand.-Mazz. (*V. minutiflora*) | Roots and rhizomes  Aerial parts | (Bos et al., 1997; Bos et al., 2000; Sundaresan et al., 2012; Fernández et al., 2015) |
|  | *β*-Ionone | *V. officinalis*;  *Valeriana montana* L. (*V. montana*) | Aerial parts | (Raina and Negi, 2015; Singh et al., 2013) |
|  | allo-Aromadendrene-epoxide | *V. sisymbriifolia* | Aerial patrs | Pirbalouti et al. (2015) |
|  | Cedrane | *V. amurensis*  *V. wallichii* | Roots and rhizomes | Bos et al. (1997) |
|  | (E)-*β*-Lonone | *V. officinalis* | Roots and rhizomes | Bos et al. (2000) |
|  | Eicosanoic acid | *V. tuberosa* | Aerial parts | Sundaresan et al. (2012) |
|  | Furfural | *V. italica*  *V. tuberosa* | Aerial parts | Sundaresan et al. (2012) |
|  | Benzenacetaldeyde | *V. italica*  *V. tuberosa* | Aerial parts | Sundaresan et al. (2012) |
|  | Faurinone | *V. officinalis* | Roots and rhizomes | Bos et al. (2000) |
|  | g-Muurolene | *V. jatamansi* | Roots and rhizomes | Jugran et al. (2019) |
|  | C_15_H_24_ | *V. jatamansi* | Roots and rhizomes | Bos et al. (1997) |
|  | Tetradecanoic acid | *V. tuberosa* | Aerial parts | Sundaresan et al. (2012) |
|  | Neophytadiene | *V. tuberosa*  *V. italica* | Aerial parts | Sundaresan et al. (2012) |
|  | C_15_H_24_O_2_ | *V. jatamansi* | Roots and rhizomes | Bos et al. (1997) |
|  | Heneicosane | *V. tuberosa*  *V. italica* | Aerial parts | Sundaresan et al. (2012) |
|  | *α*-Thujene | *V. officinalis* | Roots and rhizomes | (Fokialakis et al., 2002; Bos et al., 2000) |
|  | Myrtenyl isovalerate | *V. officinalis* | Roots and rhizomes | (Raal et al., 2007; Bos et al., 2000) |
|  | Sesquiterpene alcohol | *V. officinalis*  *V. fauriei* | Roots and rhizomes | (Chung et al., 2012; Raal et al., 2007) |
|  | *β*-Sesquiphellandrene | *V. fauriei* | Roots and rhizomes | Chung et al. (2012) |
|  | *γ*-Murolene | *V. officinalis* | Roots and rhizomes | Bos et al. (2000) |
|  | Cedrol | *V. fauriei* | Roots and rhizomes | Chung et al. (2012) |
|  | Linalyl isoacetate | *V. officinalis* | Roots | Raal et al. (2008) |
|  | *β*-Bisabolol | *V. officinalis*  *V. jatamansi* | Rhizomes | (Maurya et al., 2021; Bos et al., 2000; Pandian and Nagarajan, 2015) |
|  | *α*-Cedrene | *V. jatamansi* | Roots and rhizomes | Bos et al. (1997) |
|  | C_15_H_26_O | *V. jatamansi* | Roots and rhizomes | Bos et al. (1997) |
|  | Iso-Propyl ios-valerate | *V. officinalis*  *V. edulis* | Roots | Dyayiya et al. (2016) |
|  | Tricosane | *V. tuberosa*  *V. italica* | Aerial parts | Sundaresan et al. (2012) |
|  | Behenic acid | *V. edulis* | Roots and rhizomes | Chen et al. (2000) |
|  | trans-Valerenyl acetate | *V. officinalis* | Roots and rhizomes | (Lunz and Stappen, 2021; Bos et al., 2000) |
|  | Mirtenyl acetate | *V. officinalis*  *V. capensis* | Roots and rhizomes | (Lunz and Stappen, 2021; Rawat et al., 2017) |
|  | trans-Valerenyl isovaleratet | *V. officinalis* | Roots | Raal et al. (2008) |
|  | 4-Terpineol | *V. jatamansi* | Rhizomes | Pandian and Nagarajan, (2015) |
|  | a-Copaene | *V. jatamansi* | Roots and rhizomes | (Jugran et al., 2019; Liu e al., 2013) |
|  | Valerenyl hexanoate | *V. officinalis* | Roots and rhizomes | Bos et al. (2000) |
|  | *β*-Eudesmol | *V. officinalis*  *V. capensis* | Roots | (Raal et al., 2008; Rawat et al., 2017; Lopes et al.; 2005) |
|  | Geranyl valerate | *V. officinalis* | Roots | Raal et al. (2008) |
|  | Valerenol | *V. officinalis* | Roots | Lopes et al. (2005) |
|  | Aromadendrene | *V. officinalis*  *V. sisymbriifolia* | Roots  Aerial parts | (Raal et al., 2008; Javidnia et al., 2012) |
|  | Geranyl isovalerate | *V. officinalis* | Roots | Raal et al. (2008) |
|  | Nootkatone | *V. officinalis* | Roots and rhizomes | Chen et al. (2015) |
|  | Patchoulyl acetate | *V. wallichii* | Roots and rhizomes | Bos et al. (1997) |
|  | *β*-Cymene | *V. wallichii*  *V. hardwickii* | Aerial parts  Roots and rhizomes | (Mathela et al., 2007; Bos et al., 1997; Sati et al., 2005) |
|  | *α*-Terpinene | *V. jatamansi* | Roots and rhizomes | Jugran et al. (2019) |
|  | 1,8-Cineole | *V. wallichii*  *V. alliariifolia*  *V. officinalis* | Roots and rhizomes  Aerial parts | (Bos et al., 1997; Taherpou et al., 2010; Singh et al., 2013) |
|  | Geraniol | *V. jatamansi* | Rhizomes | Pandian and Nagarajan, (2015) |
|  | *β*-Acoradienol | *V. jatamansi* | Roots | Verma et al. (2011) |
|  | d-Cadinene | *V. jatamansi* | Roots and rhizomes | Jugran et al. (2019) |
|  | o-Cymene | *V. jatamansi* | Roots and rhizomes | Jugran et al. (2019) |
|  | p-Cymene | *V. officinalis*  *V. jatamansi*  *V. sisymbriifolia*  *V. alliariifolia* | Roots and rhizomes | (Raal et al., 2008; Samaneh et al., 2010) |
|  | Carene | *V. officinalis*  *V. officinalis* | Roots and rhizomes | (Chen et al., 2015; Yu et al., 2011) |
|  | Furfuryl 3-methylbutanoate | *V. jatamansi* | Rhizomes | Pandian and Nagarajan, (2015) |
|  | Mualiol | *V. jatamansi* | Roots and rhizomes | Ming et al. (1994) |
|  | Nelolidol | *V. jatamansi* | Roots and rhizomes | Ming et al. (1994) |
|  | trans-Sabinyl acetate | *V. officinalis* | Roots and rhizomes | Bos et al. (2000) |
|  | 1,2,6-Hexanetriol | *V. jatamansi* | Rhizomes | Pandian and Nagarajan, (2015) |
|  | *α*-Terpinyl acetate | *V. officinalis*  *V. fauriei* | Roots and rhizomes | (Chung et al., 2012; Bos et al., 2000; Yu et al., 2011) |
|  | Terpinen-1-ol | *V. officinalis*  *V. wallichii* | Roots and rhizomes | (Bos et al., 1997; Bos et al., 2000) |
|  | cis-*α*-Bisabolene | *V. sisymbriifolia* | Roots and rhizomes | Pirbalouti et al. (2015) |
|  | Docosane | *V. tuberosa*  *V. italica* | Aerial parts | Sundaresan et al. (2012) |
|  | Carvacrol methyl ether | *V. italica*  *V. wallichii*  *V. hardwickii* | Roots and rhizomes | (Mathela et al., 2009; Bos et al., 1997; Sundaresan et al., 2012) |
|  | a-Cadinene | *V. jatamansi* | Roots and rhizomes | Jugran et al. (2019) |
|  | Methyl thymohydroquinone | *V. hardwickii* | Roots and rhizomes | Mathela et al. (2007) |
|  | Cyclolongifolene oxide | *V. jatamansi* | Rhizomes | Pandian and Nagarajan, (2015) |
|  | *α*-Copaene | *V. jatamansi* | Rhizomes | Fokialakis et al. (2002) |
|  | Ledene oxide-(II) | *V. jatamansi* | Rhizomes | Pandian and Nagarajan, (2015) |
|  | 1,8-Cineol | *V. jatamansi* | Roots | Liu e al. (2013) |
|  | Carveol | *V. officinalis*  *V. capensis* | Roots | (Raal et al., 2008; Rawat et al., 2017) |
|  | Sabinol | *V. officinalis* | Roots and rhizomes | Chen et al. (2015) |
|  | Carvacryl acetate | *V. jatamansi* | Roots and rhizomes | Jugran et al. (2019) |
|  | Isobutyl valerate | *V. officinalis*  *V. edulis* | Roots and rhizomes | (Bos et al., 2000; Samaneh et al., 2010) |
|  | Eugenol | *V. officinalis*  *V. wallichii*  *V. tuberosa*  *V. minutiflora* | Roots and rhizomes  Aerial parts | (Sati et al., 2005; Bos et al., 2000), (Sundaresan et al., 2012; Fernández et al., 2015) |
|  | Eugenyl acetate | *V. officinalis* | Aerial parts | Singh et al. (2013) |
|  | Geranial | *V. minutiflora* | Aerial parts | Fernández et al. (2015) |
|  | Piperitone | *V. capensis* | Roots | Rawat et al. (2017) |
|  | p-Menth-l-en-9-al | *V. minutiflora* | Aerial parts | Fernández et al. (2015) |
|  | Santalol | *V. jatamansi* | Roots | Thusoo et al. (2014) |
|  | 2*α*,4a*β*,8a*β*-Decahydro-2-naphthalenol | *V. jatamansi* | Rhizomes | Pandian and Nagarajan, (2015) |
|  | Farnesol | *V. officinalis* | Roots | Lunz and Stappen, (2021) |
|  | *α*-Panasinsene | *V. officinalis* | Roots and rhizomes | (Lunz and Stappen, 2021; Wang et al., 2010) |
|  | Isoborneol | *V. officinalis* | Roots and rhizomes | Bos et al. (2000) |
|  | Longipinene | *V. jatamansi* | Roots and rhizomes | Jugran et al. (2019) |
|  | 2,6-Dimethoxy-p-cymene | *V. officinalis*  *V. wallichii* | Roots and rhizomes | (Raal et al., 2008; Bos et al., 2000) |
|  | Dehydroisolongifolene | *V. officinalis* | Roots | Raal et al. (2008) |
|  | Isoamyl isovalerate | *V. officinalis* | Roots and rhizomes | (Raal et al., 2008; Bos et al., 2000) |
|  | Turmerol | *V. jatamansi* | Roots and rhizomes | Bos et al. (1997) |
|  | C_15_H_26_O_2_ | *V. jatamansi* | Roots and rhizomes | Fokialakis et al. (2002) |
|  | Valerenal isomer | *V. jatamansi* | Roots and rhizomes | Fokialakis et al. (2002) |
|  | Curcuphenyl acetate | *V. jatamansi* | Roots and rhizomes | Bos et al. (1997) |
|  | C_17_H_24_O_2_ (Xanthorrhizyl acetate) | *V. jatamansi* | Roots and rhizomes | Fokialakis et al. (2002) |
|  | *α*-Kessyl acetate | *V. jatamansi* | Roots and rhizomes | Bos et al. (1997) |
|  | Dimethyl | *V. sisymbriifolia* | Roots and rhizomes | Fokialakis et al. (2002) |
|  | p-Cresol | *V. sisymbriifolia* | Roots and rhizomes | Pirbalouti et al. (2015) |
|  | 2,6-Dimethyl anisole | *V. sisymbriifolia* | Roots and rhizomes | Pirbalouti et al. (2015) |
|  | 2,5-Dimethoxy-p-cymene | *V. wallichii*  *V. officinalis* | Roots and rhizomes | (Bos et al., 1997; Pavlović et al., 2007) |
|  | Geranyl acetate | *V. wallichii* | Roots and rhizomes | Bos et al. (1997) |
|  | Humulene-oxide | *V. jatamansi* | Whole plants | Agnihotri et al. (2011) |
|  | *γ*-Patchoulene | *V. jatamansi*  *V. wallichii* | Roots and rhizomes | (Bos et al., 1997; Raal et al., 2008) |
|  | *α*-Calacorene | *V. officinalis* | Roots and rhizomes | Bos et al. (2000) |
|  | Methyl linoleate | *V. hardwickii* | Roots | Das et al. (2011) |
|  | *δ*-Guaiene | *V. jatamansi* | Roots | Bhatt et al. (2012) |
|  | Seychellene | *V. officinalis*  *V. jatamansi*  *V. wallichii*  *V. officinalis* | Roots and rhizomes | (Pandian and Nagarajan, 2015; Raina and Negi, 2015; Lokar et al., 1989; Bhatt et al., 2012) |
|  | *α*-Bulnesene | *V. jatamansi*  *V. officinalis* | Roots and rhizomes | (Raal et al., 2008; Jugran et al., 2019) |
|  | *γ*-Cadinene | *V. officinalis* | Roots | Raal et al. (2008) |
|  | *δ*-Cadinene | *V. wallichii*  *V. officinalis*  *V. jatamansi* | Roots and rhizomes | (Bos et al., 1997; Raal et al., 2008), Liu e al. (2013) |
|  | Viridiflorol | *V. officinalis*  *V. jatamansi*  *V. sisymbriifolia*  *V. italica* | Roots and rhizomes | (Raal et al., 2008; Pirbalouti ett al., 2015; Sundaresan et al., 2012; Thusoo et al., 2014; Lokar et al., 1989) |
|  | DL-limonene | *V. jatamansi* | Roots and rhizomes | Pandian and Nagarajan, (2015) |
|  | *α*-Patchoulene | *V. jatamansi* | Roots | Jugran et al. (2019) |
|  | I-Propyl 6,9,12-hexadecatrienoate | *V. jatamansi* | Rhizomes | Pandian and Nagarajan, (2015) |
|  | Aristolone | *V. officinalis*  *V. officinalis*  *V. jatamansi* | Roots and rhizomes | Jugran et al. (2019) |
|  | Eucalyptollin | *V. jatamansi* | Rhizomes | Fokialakis et al. (2002) |
|  | Isopatchoulane | *V. jatamansi* | Roots and rhizomes | Alfaro-Romero et al. (2016) |
|  | Drimenol | *V. wallichii* | Roots and rhizomes | Bos et al. (1997) |
|  | Isolongifolen-5-one | *V. jatamansi* | Rhizomes | Pandian and Nagarajan, (2015) |
|  | cis-*β*-Guaiene | *V. jatamansi* | Rhizomes | Pandian and Nagarajan, (2015) |
|  | Calarene-*β*-gurjunene | *V. jatamansi* | Roots and rhizomes | Thusoo et al. (2014) |
|  | trans-α-Bergomotene | *V. wallichii* | Aerial parts  Roots | Sati et al. (2005) |
|  | Cryptofauronyl acetate | *V. wallichii* | Roots and rhizomes | Bos et al. (1997) |
|  | Pacifigoriadiene isomer B | *V. officinalis* | Roots | Raal et al. (2008) |
|  | Humulene epoxide-Ⅱ | *V. jatamansi* | Roots | Agnihotri et al. (2011) |
|  | *γ*-Terpinene | *V. jatamansi* | Roots | Fokialakis et al. (2002) |
|  | epi-Bicyclosesquiphellandrene | *V. officinalis* | Roots | Raal et al. (2008) |
|  | *β*-Cubebene | *V. jatamansi* | Rhizomes | Mathela et al. (2009) |
|  | *α*-cis-Bergamotene | *V. jatamansi* | Rhizomes | Pandian and Nagarajan, (2015) |
|  | Curcuphenol isomer | *V. jatamansi* | Rhizomes | (Bos et al., 1997; Mathela et al., 2009) |
|  | Kanokonylacetate | *V. jatamansi* | Rhizomes | Mathela et al. (2009) |
|  | Baldrinal | *V. jatamansi* | Rhizomes | Pandian and Nagarajan, (2015) |
|  | Veridiflaral | *V. jatamansi* | Roots | Bhatt et al. (2012) |
|  | 4-Methoxy-8-pentyl-1-naphthoic acid | *V. jatamansi* | Rhizomes | Fokialakis et al. (2002) |
|  | Selina-3,7(11)-dien | *V. jatamansi* | Roots | Liu e al. (2013) |
|  | 4-Terpineal | *V. jatamansi* | Roots | Bhatt et al. (2012) |
|  | Carvacrol | *V. italica*  *V. capensis* | Roots | (Sundaresan et al., 2012; Rawat et al., 2017) |
|  | *δ*-Selinene | *V. officinalis*  *V. officinalis*  *V. jatamansi* | Aerial parts | Taherpour et al. (2010) |
|  | Phenylethyl alcohol | *V. jatamansi* | Rhizomes | Pandian and Nagarajan, (2015) |
|  | Menthone | *V. officinalis* | Roots | Raal et al. (2008) |
|  | Bornylvalerate | *V. jatamansi* | Rhizomes | Fokialakis et al. (2002) |
|  | 1-Heptatriacotanol | *V. jatamansi* | Rhizomes | Pandian and Nagarajan, (2015) |
|  | 7-epi-*α*-Selinen | *V. officinalis*  *V. jatamansi* | Roots and rhizomes | (Raal et al., 2008; Thusoo et al., 2014) |
|  | 6-(1-Hydroxymethylvinyl)-4,8a-dimethyl-3,5,6,7,8,8a-hexahydro-1h-naphthalen-2-one | *V. jatamansi* | Rhizomes | Pandian and Nagarajan, (2015) |
|  | Maaliol | *V. officinalis*  *V. jatamansi*  *V. wallichii* | Roots and rhizomes  Aerial parts | (Sati et al., 2005; Raal et al., 2008), (Maurya et al., 2021; Thusoo et al., 2014) |
|  | Ethyl valerate | *V. jatamansi* | Rhizomes | Pandian and Nagarajan, (2015) |
|  | (–)-*α*-Selinene | *V. jatamansi* | Roots and rhizomes | Pandian and Nagarajan, (2015) |
|  | Longifolenaldehyde | *V. jatamansi* | Roots and rhizomes | Alfaro-Romero et al. (2016) |
|  | *α*-Zingiberene | *V. jatamansi* | Rhizomes | Mathela et al. (2009) |
|  | Limonen-6-ol, pivalate | *V. jatamansi* | Rhizomes | Pandian and Nagarajan, (2015) |
|  | Patchouli alcohol | *V. officinalis*  *V. jatamansi*  *V. wallichii*  *V. edulis* | Roots and rhizomes  Aerial parts | (Raal et al., 2008; Thusoo et al., 2014; Dyayiya et al., 2016; Jugran et al., 2019) |
|  | Curcuphenol | *V. jatamansi* | Rhizomes | Mathela et al. (2009) |
|  | Beyerene | *V. capensis* | Roots | Rawat et al. (2017) |
|  | Ipsdienol | *V. capensis* | Roots | Rawat et al. (2017) |
|  | 3',8,8'-Trimethoxy-3-piperidyl-2,2'-binaphthalene-1,1',4,4'-tetrone | *V. jatamansi* | Rhizomes | Pandian and Nagarajan, (2015) |
|  | *α*-Alaskene | *V. capensis* | Roots | Rawat et al. (2017) |
|  | n-Hexyl isovalerate | *V. officinalis* | Roots | Fokialakis et al. (2002) |
|  | Isomenthyl acetate | *V. officinalis* | Roots | Raal et al. (2008) |
|  | Valencene ketone | *V. officinalis* | Roots | Fokialakis et al. (2002) |
|  | (E)‐*β*‐Ocimene | *V. officinalis* | Aerial parts | Raina and Negi, (2015) |

**References**

Agnihotri, S., Wakode, S., and Ali, M. (2011). Chemical composition, antibacterial and topical anti-inflammatory activity of *Valeriana jatamansi* Jones essential oil. *J*. *Essent*. *Oil Bear Pl*. *Jeop*. 14, 417–422. doi:10.1080/0972060X.2011.10643596

Alfaro-Romero, A., Balderas-López, J.L., Duarte-Lisci, G., and Navarrete, A. (2016). Root scent composition in *Valeriana officinalis* and *Valeriana edulis* ssp. procera analyzed by HS-SPME-GC-MS a. *J*. *Essent*. *Oil Bear Pl*. 19, 1821–1825.doi:10.1080/0972060x.2015.1107512

Alfaro-Romero, A., Balderas-López. J. L., Tavares-Carvalho, J. C., and Navarrete, A. (2021). Valeiridoside, an iridoid xyloside from *Valeriana procera* with anxiogenic effect in mice. *Rev*. *Bras*. *Pharmacogn*. 31, 85–90.doi:[10.1007/S43450-021-00140-X](https://schlr.cnki.net/Detail/doi/GARJ2021_1/SSJDBE9F1F46E4F0D530BC5F64A51E859029)

Amanzadeh, Z. Y., Ghassemi, D. N., Ebrahimi, S. S., and Pirali, H. M. (2002). Two new valepotriates from the roots of *Valeriana sisymbriifolia*. *Daru*. 10, 63–66.doi:

Bhatt, I. D., Dauthal, P., Rawat, S., Gaira, K. S., Jugran, A., Rawal, R. S., et al. (2012). Characterization of essential oil composition, phenolic content, and antioxidant properties in wild and planted individuals of *Valeriana jatamansi* Jones. *Sci*. *Hortic*. 136, 61–68.doi:10.1016/j.scienta.2011.12.032

Bos, R., Woerdenbag, H. J., Hendriks, H., Smit, H. F., Wikström, H. V., and Scheffer, J. C. (1997). Composition of the essential oil from roots and rhizomes of *Valeriana wallichii* DC. *Flavour*. *Fragr*. *J*. 12(2), 123–131.doi: 10.1002/(SICI)1099-1026(199703)12:2<123::AID-FFJ613>3.0.CO;2-4

Bos, R., Hendriks, H., Pras, N., Stojanova, A. S., and Georgiev, E. V. (2000). Essential oil composition of *Valeriana officinalis* ssp. collina cultivated in bulgaria. *J*. *Essent*. *Oil Res*. 12, 313–316.doi:10.1080/10412905.2000.9699524

Bos, R., Woerdenbag, H. J., and Pras, N. (2002). Determination of valepotriates. *J*. *Chromatogr*. *A*. 967, 131–146.doi:10.1016/S0021-9673(02)00036-5

Chen, H. W., Wei, B. J., He, X. H., Liu, Y., and Jie, W. (2015). Chemical components and cardiovascular activities of Valeriana spp. *Evid*. *Based Complement*. *Altern*. *Med*. 2015, 947619.doi:10.1155/2015/947619

Chen, L., Qin, L. P., and Zheng, H. C. (2000). Chemical constituents, plant resources and pharmacological activities of Valerian. *J*. *Pharm*. *Prac*. 18, 277–279.doi:

Chen, Y. G., Yu, L. L., Huang, R., Lv. Y. P., and Gui, S. H. (2005). 11-Methoxyviburtinal, a new iridoid from *Valeriana jatamansi*. *Arch*. *of Pharm*. *Res*. 28, 1161–1163.doi:[10.1007/BF02972980](https://doi.org/10.1007/bf02972980)

Chung, I. M., Kim, E. H., and Moon, H. I. (2012). Retracted: immunotoxicity activity of the major essential oils of *Valeriana fauriei* briq against Aedes aegypti L. *Immunopharm*. *Immunot*. 34(3), 107–110.doi:10.3109/08923973.2010.484839

Das, J., Mao, A. A., and Handique, P. J. (2011). Volatile constituents of *V. hardwickii* root oil from arunachal pradesh, eastern himalaya. *Rec*. *Nat*. *Prod*. 5, 70–73.doi:10.1055/s-0030-1250161

Ding, F., Fang, Y., Wen, L., and Liu, Y. W. (2011). Comparative study on sedative and hypnotic effects of volatile oil and water extract of *Valeriana officinalis*. *China Pharm*. 14, 1411–1413.doi:10.3969/j.issn.1008-049X.2011.10.003

Dong, F. W., Wu, Z. K., Yang, L., Zi, C. T., Yang, D., Ma, R. J., et al. (2015). Iridoids and sesquit-erpenoids of *Valeriana stenoptera* and their effects on NGF-induced neurite outgrowth in PC12 cells. *Phytochemistry*. 118, 51–60.doi:10.1016/j.phytochem.2015.08.015

Du, X. W., and Wu, J. K. (2006). Chemical and pharmacological progress of *Valeriana genera*. *World Phytomedicine*. 21, 10–14.doi:10.7501/j.issn.1674-5515.2006.1.036

Dyayiya, N. A., Oyemitan, I. A., Matewu, R., Oyedeji, O. O., Oluwafemi, S. O., Nkeh-Chungag, B. N., et al. (2016). Chemical analysis and biological potential of Valerian root as used by herbal practitioners in the Eastern Cape Province, South Africa. *Afr*. *J*. *Tradit*. *Complem*. 13, 114–122.doi:10.4314/ajtcam.v13i1.16

Fernández, S., Rondón, M., Rojas, J., Morales, A., and Rojas-Fermin, L. (2015). Comparison of the chemical composition of *Valeriana parviflora* essential oils collected in the Venezuelan Andes in two different seasons. *Nat*. *Prod*. *Commun*. 10, 657–659.doi:10.1007/978-3-319-10963-3_198

Fernández, S., Wasowski, C., Paladini, A. C., and Marder, M. (2004). Sedative and sleep-enhancing properties of linarin, a flavonoid-isolated from *Valeriana officinalis*. *Pharmacol*. *Biochem*. *Be*. 77, 399–404.doi:10.1016/j.pbb.2003.12.003

Fokialakis, N., Prokopios, M., and Mitaku, S. (2002). Essential oil constituents of *Valeriana italica* and *Valeriana tuberosa*. stereochemical and conformational study of 15-acetoxy valeranone. *Z*. *Naturforsch*. *C*. 57, 791–796.doi:10.1515/znc-2002-9-1006

Granicher, F., Christen, P., Kamalaprija, P., and Burger, U. (1995). An iridoid diester from *Valeriana officinalis* var. sambucifolia hairy roots. *Phytochemistry*. 38, 103–105.doi:10.1016/0031-9422(95)00532-3

Guo, Y. Q., Xu, J., Li, Y. H., Watanabe, R., Oshima, Y., Yamakuni, T., et al. (2006). Iridoids and sesquiterpenoids with NGF-potentiating activity from the rhizomes and roots of *Valeriana fauriei*. *Chem*. *Pharm*. *Bull*. 54, 123–125.doi:10.1002/chin.200632218

Han, Z. Z., Yan, Z. H., Liu, Q. X., Hu, X. Q., Ye, J., Li, H. L., et al. (2012). Acylated iridoids from the roots of *Valeriana officinalis* var. latifolia. *Planta Med*. 78, 1645–1650.doi:[10.1055/s-0032-1315214](https://doi.org/10.1055/s-0032-1315214)

Holzl, J., Chari, V. M., and Seligmann, O. (1976). Structure of 3 genuine valtrate hydrines from *Valeriana tiliaefolia*. *Tetrahedron Lett*. 17, 1171–1174.

Holzl, J., and Koch, U. (1984). The Compounds of *Valeriana alliariifolia*. *Planta Med*. 50, 458.doi:10.1055/s-2007-969771

Irshad, M., Aziz, S., Rehman, H. U., and Hussain, H. (2012). GC-MS analysis and antifungal activity of essential oils of Angelica glauca, Plectranthus rugosus and *Valeriana wallichii*. *J*. *Essent*. *Oil Bear Pl*. 15, 15–21.doi:[10.1080/0972060X.2012.10644014](https://schlr.cnki.net/Detail/doi/GARJ2012/STJD487AD663217236CAC15BDB5B34F542EC)

Javidnia, K., Miri, R., Kamalinejad, M., and Khazraei, H. (2010). Chemical composition of the volatile oil of aerial parts of *Valeriana sisymbriifolia* vahl. grown in iran. *Flavour*. *Frag*. *J*. 21, 516–518.doi:10.1002/ffj.1660

Kırmızıbekmeza, H., Kúszb, N., Bérdic, P., Zupkóc, I., and Hohmann, J. (2018). New iridoids from the roots of *V. dioscoridis* *Fitoterapia*.130, 73–78.doi:10.1016/j.fitote.2018.08.007

Koch, U., and Hoelzl, J. (1985). Constituents of *Valeriana alliariifolia*. 2.valepotriathydrines. *Planta Med*. 51, 172–173.

Kucaba, W., Thies, P. W., and Finner, E. (1980). Isodidrovaltratum, ein neues valepotriat aus Valeriana vaginata. *Phytochemistry*. 19, 575–577.doi:10.1016/0031-9422(80)87018-X

Li, Y. D., Li, R. T., and Li. H. Z. (2011). Study on the chemical compositions of *Valeriana Jatamansi*. *Yunnan J*. *Tradit*. *Chin*. *Med*. *Mater*. *Med*. 32, 80–81.doi: 10.16254/j.cnki.53-1120/r.2011.06.048.

Li, Y. D., Wu, Z. Y., Li, H. M., Li, H. Z., and Li, R. T. (2013). Iridoids from the Roots of *Valeriana jatamansi*. *Helv*. *Chim*. *Acta*. 96, 424–430.doi:10.1002/hlca.201100465

Lin, S., Chen, T., Fu, P., Ye, J., Yang, X. W., Shan, L., et al. (2015). Three decomposi-tion products of valepotriates from *Valeriana jatamansi* and their cytotoxic activity. *J*. Asian *Nat*. *Prod*. *Res*. 17, 455–461. doi:10.1080/10286020.2015.1041933

Lin, S., Chen, T., Liu, X. H., Shen, Y. H., and Wang, H. (2010). Iridoids and lignans from *Valeriana jatamansi*. *J*. *Nat*. *Prod*. 73, 632–638.doi:10.1021/np900795c

Lin, S., Fu, P., Chen, T., Ye, J., Su, Y. Q., Yang, X. W., et al. (2014). Minor valepotriates from *Valeriana jatamansi* and their cytotoxicity against metastatic prostate cancer cells. *Planta Med*. 81, 56–61.doi:10.1055/s-0034-1383369

Liu, X. C., Zhou, L. G., and Zhi, L. L. (2013). Identification of insecticidal constituents from the essential oil of *Valeriana jatamansi* Jones against liposcelis bostrychophila badonnel. *J*. *Chem*. 18, 5684–5696.doi:10.1155/2013/853912

Liu, X. X., Duan. X. Y., Fan. H., Wang, H. F., Jiang, X. G., Fang, Y., Tan, Q., et al. (2021). 8-hydroxypinoresinol-4-O-beta-D-glucoside from *Valeriana officinalis* L. is a novel Kv1.5 channel blocker. *J*. *Ethnopharmacol*. 276, 114168.doi: [10.1016/j.jep.2021.114168](https://doi.org/10.1016/j.jep.2021.114168)

Liu, Y. H., Wu, P. Q., Hu, Q. L., Pei, Y. J., Qi, F. M., Zhang, Z. X., et al. (2017). Cytotoxic and antibacterial activities of iridoids and sesquiterpenoids from *Valeriana jatamansi*. *Fitoterapia*. 123, 73–78.doi:[10.1016/j.fitote.2017.09.011](https://doi.org/10.1016/j.fitote.2017.09.011)

Lokar, L. C., and Moneghini, M. (1989). Geographical variation in the monoterpenes of *Valeriana officinalis* leaf. *Biochem*. *Syst*. *Ecol*. 17, 563–567.doi:10.1016/0305-1978(89)90100-2

Lopes, D., Strobl, H., and Kolodziejczyk, P. (2005). Influence of drying and distilling procedures on the chemical composition of Valerian oil (*Valeriana officinalis* L.). *J*. *Essent*. *Oil Bear Pl*. 8, 134–139.doi:10.1080/0972060X.2005.10643433

Lunz, K., and Stappen, I. (2021). back to the roots an overview of the chemica1 composition and bioactivity of se1ected root essentia1 oi1s. *Molecules*. 26(11), 3155.doi:[10.3390/molecules26113155](https://doi.org/10.3390/molecules26113155)

Mathela, C. S., Chanotiya, C. S., Sati, S., Sammal, S. S., and Wray, V. (2007). Epoxysesquithujene, a novel sesquiterpenoid from *Valeriana hardwickii* var. hardwickii. *Fitoterapia*. 78, 279–282.doi:[10.1016/j.fitote.2007.01.005](https://doi.org/10.1016/j.fitote.2007.01.005)

Mathela, C. S., Chanotiya, C. S., Sati, S., Sammal, S. S., and Wray, V. (2007). Epoxysesquithujene, a novel sesquiterpenoid from *Valeriana hardwickii* var. *Fitoterapia*. 78, 279-282.doi:10.1016/j.fitote.2007.01.005

Mathela, C. S., Padalia, R. C., and Chanotiya, C. S. (2009). Kanokonyl acetate-rich Indian Valerian from Northwestern Himalaya. *Nat*. *Prod*. *Commun*. 4, 1253–1256.doi:10.1002/mnfr.200800065

Mathela, C. S., Tiwari, M., Sammal, S. S., and Chanotiya, C. S. (2005). *Valeriana wallichii* DC, A new chemotype from northwestern himalaya. *J*. *Essent*. *Oil Res*. 17, 672–675.doi:[10.1080/10412905.2005.9699029](https://schlr.cnki.net/Detail/doi/GARJ0010_1/STJD225730522E32B80C4677E774A378E2E8)

Maurya, A. K., Kumar, A., Agnihotri, V. K. (2020). New iridoids from the roots of *Valeriana jatamansi* Jones. *Nat*. *prod*. *Res*. 9, 1–8.doi:[10.1080/14786419.2020.1858412](https://doi.org/10.1080/14786419.2020.1858412)

Maurya, A. K., Sharma, A., Kumar, K., Chander, R., Kumar, A., Kumar, D., et al. (2021). Comparative studies of essential oils composition and cytotoxic activity of *Valeriana jatamansi* Jones. *J*. *Essent*. *Oil Res*. 33, 584–591.doi:10.1080/10412905.2021.1966846

Ming, D. S., Guo, J. X., Shun, Q. S., Li, Y., Liu, H. L., and Wang, T. J. (1994). Determination of chemical constituents of the essential oil from four kinds of *Valeriana officialis* L. by GC-MS. *Chin*. *Tradit*. *Patent Med*. 16, 41–42.doi:CNKI:SUN:ZCYA.0.1994-01-025

Nishiya, K., Kimura, T., Takeya, K., and Itokawa, H. (1994). Sesquiterpenoids and iridoid glycosides from *Valeriana fauriei*. *Phytochemistry*. 36, 1547–1548.doi:10.1016/S0031-9422(00)89759-9

Pandian, D. S., and Nagarajan, N. S. (2015). Comparison of chemical composition and antioxidant potential of hydrodistilled oil and supercritical fluid CO_2_ extract of *Valeriana wallichi* DC. *J*. *Nat*. *Prod*. *Res*. 1, 25–30.doi: org/10.1016/j.indcrop.2012.01.014

Pavlović, M., Kovačević, N., Tzakou, O., and Couladis, M. (2007). Composition of the essential oils from the aerial parts of five wild growing *Valeriana* species. *J*. *Essent*. *Oil Res*. 19, 433–438.doi:[10.1080/10412905.2007.9699945](https://schlr.cnki.net/Detail/doi/GARJ0010_3/STJD72CBCF3A8036B47195EE82179B05BE16)

Pavlović, M., Kovačević, N., Tzakou, O., and Couladis, M. (2004). The essential oil of *Valeriana officinalis* L. s.l. growing wild in western serbia. *J*. *Essent*. *Oil Res*. 16, 397–399.doi: 10.1080/10412905.2004.9698753

Pirbalouti, A. G., Ghahfarokhi, B. B., Ghahfarokhi, S., and Malekpoor, F. (2015). Chemical composition of essential oils from the aerial parts and underground parts of Iranian *Valerian* collected from different natural habitats. *Ind*. *Crop*. *Prod*. 63, 147–151.doi:10.1016/j.indcrop.2014.10.017

Popov, S., Handjieva, N., and Marekov, N. (1974). A new valepotriate: 7-epi-deacetylisovaltrate from *Valeriana officinalis*. *Phytochemistry*. 1, 2815–2818.doi:10.1016/0031-9422(74)80247-5

Raal, A., Arak, E., Orav, A., Kailas, T., and Müürisepp, M. (2008). Variation in the composition of the essential oil of commercial *Valeriana officinalis* L. roots from different countries. *J*. *Essent*. *Oil Res*. 20, 524–529.doi:10.1080/10412905.2008.9700079

Raal, A., Orav, A., Arak, E., Kailas, T., and Müürisepp, M. (2007). Variation in the composition of the essential oil of *Valeriana officinalis* L. roots from estonia. *P*. *Est*. *Acad*. *Ences*. *Chem*. 56, 67–74.doi:10.1016/S1383-5866(02)00153-3

Raina, A. P., and Negi, K. S. (2015). Essential oil composition of *Valeriana jatamansi* Jones from Himalayan regions of India. *Indian J*. *Pharma*. *Sci*. 77, 218–222.doi:10.4103/0250-474X.156614

Rawat, S., Jugran, A. K., Bhatt, I. D., Rawal, R. S., and Dhar, U. 2017 (). Essential oil composition and antioxidant activity in *Valeriana jatamansi* jones: influence of seasons and growing sources. *J*. *Essent*. *Oil Res*. 29, 1–7.doi:10.1080/10412905.2016.1189856

Salles, L., Ls, A., Rech, S. B., Zanatta, N., and Poser, G. V. (2000). Constituents of *Valeriana glechomifolia* meyer. *Biocheml*. *Syst*. *Ecol*. 28, 907–910.doi:10.1016/S0305-1978(99)00124-6

Samaneh, E. T., Tayebeh, R., Hassan, E., and Vahid, N. (2010). Composition of essential oils in subterranean organs of three species of *Valeriana* L. *Nat*. *Prod. Res*. 24, 1834–1842.doi: 10.1080/14786419.2010.482051

Sati, S., Chanotiya, C. S., and Mathela, C. S. (2005). Comparative investigations on the leaf and root oils of *Valeriana wallichii* DC from northwestern himalaya. *J*. *Essent*. *Oil Res*. 17(4), 408–409.doi:[10.1080/10412905.2005.9698945](https://schlr.cnki.net/Detail/doi/GARJ0010_1/STJDFEBD780CA38797D62BE95DCFBBFC37F4)

Singh, S. K., Katoch, R., and Kapila, R. K. (2013). Chemotypic variation for essential oils in *Valeriana jatamansi* Jones populations from Himachal Pradesh. *J*. *Essent*. *Oil Res*. 25, 154–159.doi:10.1080/10412905.2013.767757

Sundaresan, V., Sahni, G., Verma, R. S., Padalia, R. C., Mehrotra, S., and Thul, S. T. (2012). Impact of geographic range on genetic and chemical diversity of Indian Valerian (*Valeriana jatamansi*) from northwestern himalaya. *Biochem*. *Genet*. 50, 797–808.doi:10.1007/s10528-012-9521-5

Taherpour, A. A., Maroofi, H., Bajelani, O., and Larijani, K. (2010). Chemical composition of the essential oil of *Valeriana alliariifolia* adams of iran. *Nat*. *Prod*. *Res*. 24, 973–978.doi:[10.1080/14786410902900010](https://doi.org/10.1080/14786410902900010)

Tan, Y. Z., Peng, C., Hu, C. J., Li, H. X., Li, W. B., He, J. L., et al. (2019). Iridoids from *Valeriana jatamansi* induce autophagy-associated cell death via the PDK1/Akt/mTOR pathway in HCT116 human colorectal carcinoma cells. *Bioorg*. *Chem*. 87, 136–141.doi:10.1016/j.bioorg.2019.03.020

Tang, Y. P., and Yu, B. (2002). Iridoids from the rhizomes and roots of *Valeriana jatamansi*. *J*. *Nat*. *Prod*. 65, 1949–1952.doi:10.1021/np0203335

Thies, P. W. (1968). Linarin-isovalerianat, ein bisher unbekanntes flavonoid aus *Valeriana wallichii* D. C. *Planta Med*. 16, 361–371.doi:10.1055/s-0028-1099922

Thies, P. W. (1970). Valerosidatum, ein iridoidesterglycosid aus valeriana-arten 7. Mitteilung über die Wirkstoffe des Baldrians. *Tetrahedron Lett*. 11, 2471–2474.doi:10.1016/S0040-4039(01)98258-9

Thusoo, S., Gupta, S., Sudan, R., Kour, J., and Bhagat, M. (2014). Antioxidant activity of essential oil and extracts of *Valeriana jatamansi* roots. *J*. *Biomed*. *Biotechnol*. 2014, 614187.doi:[10.1155/2014/614187](https://doi.org/10.1155/2014/614187)

Verma, R. S., Padalia, R. C., and Chauhan, A. (2012). Assessment of similarities and dissimilarities in the essential oils of patchouli and Indian Valerian. *J*. *Essent*. *Oil Res*. 24, 487–491.doi:10.1080/10412905.2012.705095

Verma, R. S., Verma, R. K., Padalia, R. C., Chauhan, A., Singh, A., and Singh, H. P. (2011). Chemical diversity in the essential oil of Indian Valerian (*Valeriana jatamansi* jones). *Chemistry & Biodiversity*. 8, 1921–1929.doi:10.1002/cbdv.201100059

Vishwakarma, S., Goyal, R., Gupta, V., and Dhar, K. L. (2016). Gabaergic effect of valeric acid from *Valeriana wallichii* in amelioration of ICV STZ induced dementia in rats. *Rev*. *Bras*. *Farmacogn*. 26, 484–489.doi: 10.1016/j.bjp.2016.02.008

Wang, J. H., Zhao, J. L., Liu, H., Zhou, L. G., Liu, Z. L., Wang, J. G., et al. (2010). Chemical analysis and biological activity of the essential oils of two valerianaceous species from China: nardostachys chinensis and *Valeriana officinalis*. *Molecules*. 15, 6411–6422.doi:10.3390/molecules15096411

Wang, P. C., Ran, X. H., Chen, R., Li, L. C., Xiong, S. S., Liu, Y. Q., et al. (2010d). Volvalerenone A, a new type of mononor sesquiterpenoid with an unprecedented 3,12-oxo bridge from *Valeriana officinalis*. *Tetrahedron Lett*. 51, 5451–5453.doi:[10.1016/j.tetlet.2010.08.023](https://schlr.cnki.net/Detail/doi/GARJ0010_6/SJES31BC52292B6FC98D07331D0CB4138A5C)

Wang, P. C., Ran, X. H., Chen, R., Luo, H. R., Liu, Y. Q., Zhou, J., et al. (2010c). Germacrane-type sesquiterpenoids from the roots of *Valeriana officinalis* var. latifolia. *J*. *Nat*. *Prod*. 73, 1563–1567.doi: 10.1021/np100452a

Wang, P. C., Ran, X. H., Chen, R., Luo, H. R., Ma, Q. Y., Liu, Y. Q., et al. (2011). Sesquiterpenoids and lignans from the roots of *Valeriana officinalis* L. *Chem*. *Biod*. 8, 1908–1913.doi: [10.1002/cbdv.201000247](https://doi.org/10.1002/cbdv.201000247)

Wang, R. J., Shi, S. J., Tan, Y. Z., Yao. L. C., and Zhu, L. X. (2021a). Chemical constituents from *Valeriana jatamansi*. *Biochem*. *Syst*. *Ecol*. 94, 104177.doi:10.1016/j.bse.2020.104177

Wang, Y., Shi, D. Q., Jiang, N., Rao, K. R., Zhang, S. X., Liu, D., et al. (2021b). A new acylated iridoid and other chemical constituents from *Valeriana jatamansi* and their biological activities. *Nat*. *Prod*. *Res*. 3, 1–8.doi:[10.1080/14786419.2021.1961255](https://doi.org/10.1080/14786419.2021.1961255)

Xu, J., Guo, Y., Jin, D. Q., Zhao, P., Guo, P., Yamakuni, T., et al. (2012d). Three new iridoids from the roots of *Valeriana jatamansi*. *J*. *Nat*. *Med*. 66, 653–657.doi:[10.1007/s11418-012-0631-5](https://schlr.cnki.net/Detail/doi/GARJ2012/SJPD120727226143)

Xue, J., Mi, Y. Y., Wang, Z. B., Sun, Y. C., Wu, Q., Wang, C. F., et al. (2016). Determination and pharmacokinetic study of four lignans in rat plasma after oral administration of an extract of *Valeriana amurensis* by ultra-high performance liquid chromatography with tandem mass spectrometry. *J*. *Sep*. *Sci*. 39, 1825–1833.doi:10.1002/jssc.201600038

Yang, B., Zhang, J. F., Song, H. Z., Gu, M. C., Zhao, H. J., and Xiong, Y. K. (2015). Two new iridoid esters from the root and rhizome of *Valeriana jatamansi* Jones. *Helv*. *Chim*. *Acta*. 98, 1225–1230.doi:10.1002/hlca.201400389

Yu, W., Yi, Y., and Yang, D. (2011). Analysis of chemical constituents of essential oil from cultured *Valeriana officinalis* L. *Chin*. *J*. *Spectro*. *Lab*. 28, 1672–1674.doi:10.1090/S0002-9939-2011-10775-5

Zhou, T., and Huang, B. K. (2008). Advancement on the chemical constituents and bioactivities of essential oil of *Valeriana officinlais* L. *Lishizhen Med*. *Mater*. *Med*. *Res*. 19(11), 2663–2664. doi: 10.3969/j.issn.1008-0805.2008.11.041

Zuo, Y. M., Xu, Y. L., Zhang, Z. L., Yan, H, and Liu, D. H. (2017a). Study on the chemical components of flavonoids of *Valeriana officinalis*. *Zhong Cao Yao*. 40, 1331–1334.doi:10.13863/j.issn1001-4454.2017.06.020

Zuo, Y. M., Yan, H., Zhang, L. Z., Xu, Y. L., and Wang, Y. F. (2017b). Chemical Components of Bisepoxylignans of *Valeriana officinalis*. *J*. *Chin*. *Med*. *Mater*. 40, 1607–1610.doi:10.13863/j.issn1001-4454.2017.07.023
